# Prdm5 Regulates Collagen Gene Transcription by Association with RNA Polymerase II in Developing Bone

**DOI:** 10.1371/journal.pgen.1002711

**Published:** 2012-05-10

**Authors:** Giorgio Giacomo Galli, Kristian Honnens de Lichtenberg, Matteo Carrara, Wolfgang Hans, Manuela Wuelling, Bettina Mentz, Hinke Arnolda Multhaupt, Cathrine Kolster Fog, Klaus Thorleif Jensen, Juri Rappsilber, Andrea Vortkamp, Les Coulton, Helmut Fuchs, Valérie Gailus-Durner, Martin Hrabě de Angelis, Raffaele Adolfo Calogero, John Robert Couchman, Anders Henrik Lund

**Affiliations:** 1 Biotech Research and Innovation Centre and Centre for Epigenetics, University of Copenhagen, Copenhagen, Denmark; 2 Molecular Biotechnology Center, Department of Clinical and Biological Sciences, University of Torino, Torino, Italy; 3 German Mouse Clinic, Institute of Experimental Genetics, Helmholtz Zentrum München, German Research Center for Environmental Health GmbH, Neuherberg, Germany; 4 Department of Developmental Biology, Center for Medical Biotechnology, University Duisburg-Essen, Essen, Germany; 5 Department of Biomedical Sciences and BRIC, University of Copenhagen, Copenhagen, Denmark; 6 Wellcome Trust Centre for Cell Biology, University of Edinburgh, Edinburgh, United Kingdom; 7 Academic Unit of Bone Biology, University of Sheffield Medical School, Sheffield, United Kingdom; 8 Chair of Experimental Genetics TUM, Freising-Weihenstephan, Germany; University of Washington, United States of America

## Abstract

PRDM family members are transcriptional regulators involved in tissue specific differentiation. PRDM5 has been reported to predominantly repress transcription, but a characterization of its molecular functions in a relevant biological context is lacking. We demonstrate here that *Prdm5* is highly expressed in developing bones; and, by genome-wide mapping of Prdm5 occupancy in pre-osteoblastic cells, we uncover a novel and unique role for Prdm5 in targeting all mouse collagen genes as well as several SLRP proteoglycan genes. In particular, we show that Prdm5 controls both Collagen I transcription and fibrillogenesis by binding inside the *Col1a1* gene body and maintaining RNA polymerase II occupancy. *In vivo*, Prdm5 loss results in delayed ossification involving a pronounced impairment in the assembly of fibrillar collagens. Collectively, our results define a novel role for Prdm5 in sustaining the transcriptional program necessary to the proper assembly of osteoblastic extracellular matrix.

## Author Summary

Bone provides the essential tensile strength of the skeletal system, constitutes an important storage for minerals, and hosts the initial differentiation stages of the hematopoietic system. In addition, bones are important endocrine organs affecting organismal metabolism. Consequently, many human diseases arise from defects in bone formation or homeostasis. Hence, deciphering the molecular mechanisms underlying bone formation is essential for understanding the basis of bone and extracellular matrix-associated diseases. Here, we provide a detailed characterization of the cellular and molecular functions of the transcription factor Prdm5 during murine bone formation *in vivo* and find that Prdm5 is expressed in skeletal structures during development and that its loss impacts the ossification process, leading to a decrease in bone mineral density. A genome-wide mapping of Prdm5 binding sites in pre-osteoblastic cells reveals an unprecedented role for a transcription factor in targeting virtually all members of the Collagen and SLRP gene families. Interestingly, Prdm5 predominantly binds exonic regions of collagen genes and associates with RNA Polymerase II to sustain Collagen I transcription.

## Introduction

PRDM proteins constitute a family of transcriptional regulators characterized by the presence of a N-terminal PR- domain that shares 20–30% similarity to the SET domain of histone methyltransferases and a variable number of zinc-finger domains typically involved in protein-DNA or protein-protein interaction [1]. Members of this family influence tissue specific differentiation as demonstrated for Prdm1 in lymphoid cell maturation [2], and Prdm16 in brown fat development [3]. Moreover, several members of the family are deregulated in pathological settings, most notably cancer, by acting either as oncogenes or tumor suppressors [1].

*PRDM5* localizes to human chromosome 4q26 and encodes, aside from the PR domain, 16 C_2_H_2_ zinc fingers. PRDM5 has previously been reported to lack intrinsic histone methyltransferase activity but to predominantly repress transcription by recruiting G9a and HDACs enzymes to target genes [4]. Furthermore, *PRDM5* has been indicated as a potential tumor suppressor in various cancers [5]–[7], but its role in mammalian development and normal physiology has not been addressed. In zebrafish, *Prdm5* loss induces morphogenic defects due to impairment of convergent extension movements at the gastrulation stage, likely resulting from deregulation of the WNT inhibitor *Dkk1* [8]. Recently, mutations in *PRDM5* were detected in Brittle Cornea Syndrome (BCS) [9], a connective tissues disease characterized by thinning of the cornea and a wide spectrum of additional symptoms including dermal and skeletal defects [10].

Bone is composed of a highly specialized, mineralized collagenous matrix that provides tensile strength to the skeletal system. Collagen I is the major component of osteoblasts matrix, composed of a heterotypic triple helix derived from Col1a1 and Col1a2 chains typically in a 2∶1 stoichiometric ratio [11], [12]. Approximately 40 collagen genes are annotated in mammalian genomes encoding around 28 proteins and, of these, type I collagen is part of the subfamily of fibrillar collagens [13]. Collagen chains are synthesized and assembled as triple helical procollagen molecules. Extracellular proteinase cleavage of N- and C-terminal telopeptides leads to mature tropocollagen that is further assembled into fibrils and fibers [14]. The latter process is regulated by other extracellular macromolecules including proteoglycans from the Small Leucine Repeat family (SLRP), such as Decorin and Fibromodulin [15], [16].

A number of transcription factors have been discovered as regulators of collagen I genes (reviewed in [17], [18]), such as Sp1 [19], Cebpβ [20] or members of the AP1 family [21]. Furthermore, a number of transcription factors are known to be key regulators of bone development, such as Runx2 [22], which controls the expression of a multitude of extracellular matrix (ECM) genes essential for both the chondrogenic and osteogenic programs [23].

We present here a novel molecular function for Prdm5 in sustaining transcription of key ECM genes. Prdm5 is highly expressed in the osteoblast region of developing bones *in vivo* and genome wide mapping of Prdm5 occupancy in osteoblastic cells identifies all collagens and a number of SLRP genes as direct targets for Prdm5. Interestingly, Prdm5 binds predominantly within the exonic regions of collagen genes and its presence dictates the amount of intragenic RNA polymerase II. Indeed, Prdm5 sustains transcription of Collagen I genes by maintaining RNA polymerase II occupancy throughout the *Col1a1* gene, while the binding to a distal enhancer element upstream of *Decorin* gene suggests a further role in chromatin organization. Osteoblasts lacking Prdm5 display decreased Collagen I and Decorin expression leading to reduced Collagen I fiber assembly *in vivo*. Downregulation of these key extracellular matrix genes likely participates in the delayed ossification and decreased bone mineral density observed in *Prdm5* mutant mice. Our data defines novel roles for Prdm5 as a transcriptional modulator of collagen genes by influencing RNA polymerase II occupancy, as well by binding to enhancer-like elements in osteoblastic cells.

## Results

### Prdm5 is expressed in developing bones of mouse embryos

**Figure 1:**
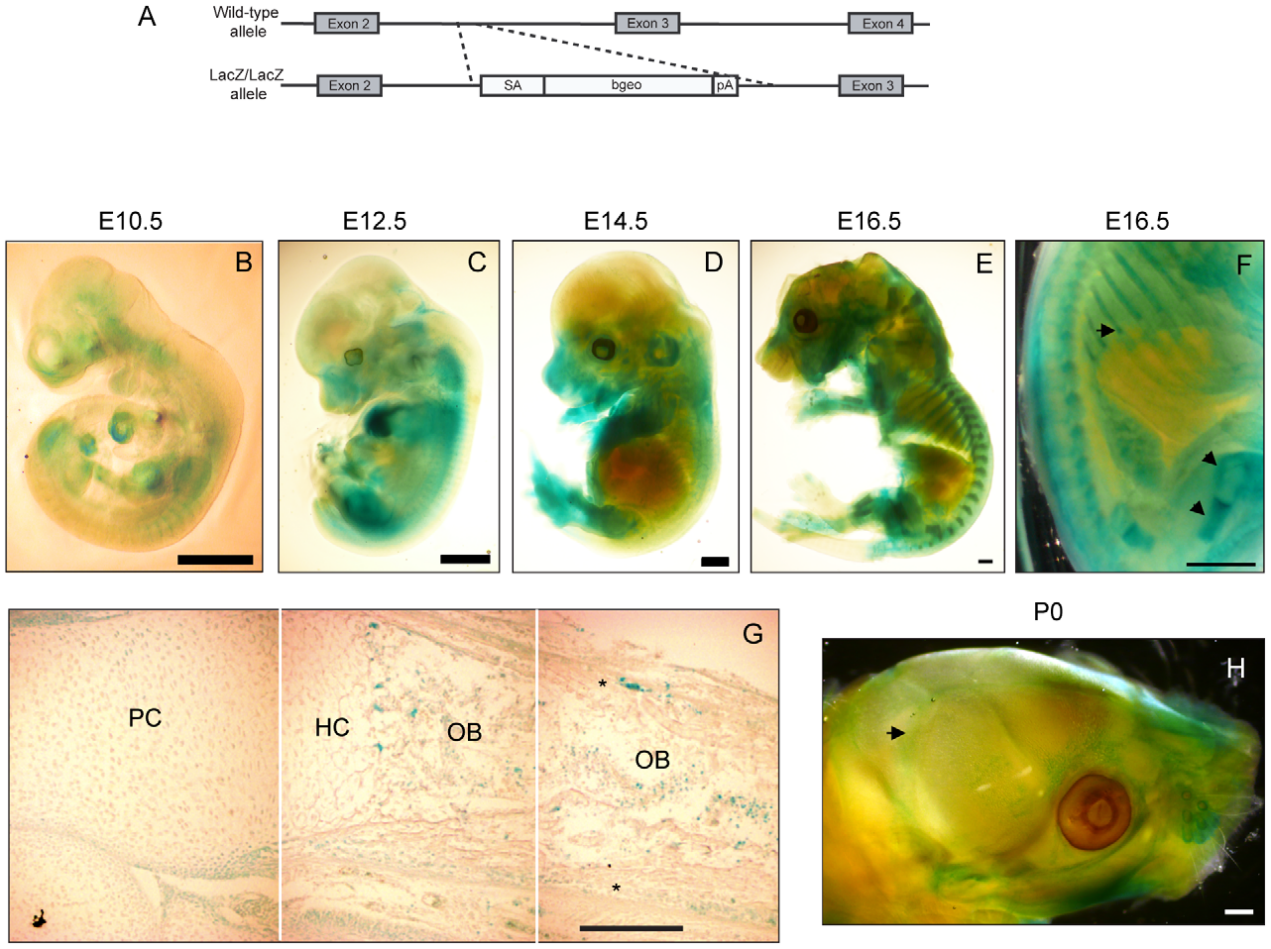
Prdm5 is expressed in osteoblast regions of developing bones. A) Scheme for the generation of the *Prdm5^LacZ/LacZ^* mouse strain. B–E) X-gal stainings of *Prdm5^LacZ/LacZ^* embryos at E10.5 (B), E12.5 (C) E14.5 (D) and E16.5 (E). F) E16.5 embryo image detail. LacZ reporter expression in the perichondrium and growth plate of femur and ribs is marked by arrows. G) X-gal staining of tibiae section from E16.5 *Prdm5* mutant embryo. Juxtaposition of three pictures (separated by white lines) to represent the whole length of a tibia. Indicated are different compartments: PC = proliferative chondrocytes, HC = hypertrophic chondrocytes, OB = osteoblasts. Periosteum is marked by asterisks. H) Whole mount X-gal staining of *Prdm5^LacZ/LacZ^* newborn skull at P0. Pronounced staining in sutures is indicated with an arrow. Bars = 1 mm, except for (G) where bar = 200 µm.

To address a possible role in mammalian development for Prdm5, a gene-trap mouse model featuring the integration of a β-galactosidase-neomycin (*β-geo*) cassette in intron 2 of the *Prdm5* gene was generated (*Prdm5^LacZ^*). This cassette is preceded by a splice acceptor site to direct exon 2-β-geo splicing and ends with a poly-A site to terminate transcription (Figure 1A). In mutant cells, expression of the *Prdm5* locus results in the production of a fusion transcript between the first two exons of *Prdm5* (≈60 amino acids) and the *β-geo* cassette with a resulting fusion protein of approximately 135 kDa in size (Figure S1A). To validate the effectiveness of the gene-trap system, we quantified the levels of the wt *Prdm5* allele in *Prdm5^LacZ/LacZ^* embryonic fibroblasts and found it to reach a maximum of 10% relative to the expression in wt littermates (Figure S1A). In adult organs, except for brain, testis and lung, we observed Prdm5 levels reduction to be at least 85% in the tissues tested (Figure S1B). In contrast to the essential role in zebrafish [8], *Prdm5^LacZ/LacZ^* mice are viable and fertile and the mutant allele segregates according to Mendelian ratios (Figure S1C). Gross pathology analysis did not reveal obvious abnormalities and no notable differences in weight were detected between mutant mice and wild type littermates up to the age of 56 weeks (Figure S1D).

To characterize the expression pattern of *Prdm5*, we tracked the β-galactosidase expression driven by the endogenous *Prdm5* promoter by whole mount X-gal staining at various developmental stages. At E10.5–12.5 the LacZ reporter was expressed in a diffuse staining pattern along meso-endodermal derived regions with higher intensity in the heart (Figure 1B and 1C). At E14.5 LacZ staining accumulated in limbs and snout regions and in particular in cartilaginous templates (Figure 1D). From E16.5 a specific staining pattern was observed in skeletal elements, particularly in long bones and ribs (Figure 1E). In these tissues LacZ was highly expressed in the osteoblastic regions including the trabecular compartment and periosteum/perichondrium (arrows in Figure 1F). LacZ staining on sections from E16.5 embryo tibia revealed that the *Prdm5* promoter is active in a subpopulation of cells in the osteoblast region (periosteum and trabecular area) (Figure 1G), whereas no signal was detected in the hypertrophic or proliferative chondrocytes zones (Figure 1G). Moreover, LacZ staining of calvariae of *Prdm5^LacZ/LacZ^* mice at P0 indicated that *Prdm5* expression in osteoblasts is not restricted to long bones but can be detected also in skull sutures and weakly in calvariae (Figure 1H). Robust *PRDM5* expression in osteoblasts was further confirmed in primary human osteoblast cells isolated from healthy donors, compared to a panel of human immortalized cell lines of different origins (Figure S1E). Moreover we found comparable Prdm5 expression levels in mouse primary calvarial osteoblasts, the osteoblastic MC3T3 cell line and primary embryonic fibroblasts (Figure S1F). In summary, *in vivo* expression analyses confirmed Prdm5 as consistently expressed in osteoblastic compartments in the mouse.

### Acute Prdm5 deregulation affects osteogenic differentiation *in vitro*

**Figure 2:**
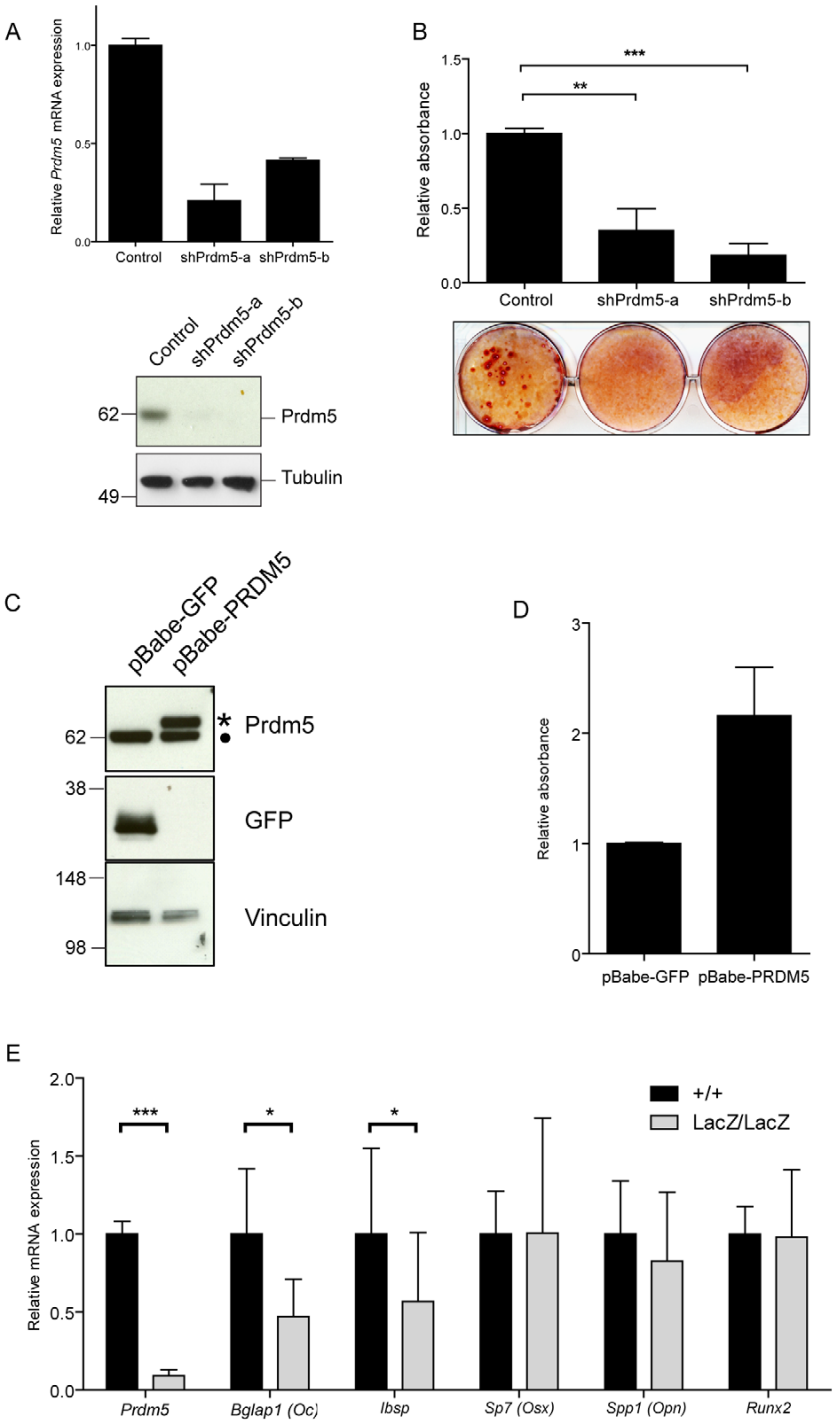
Prdm5 deregulation impairs osteogenic differentiation *in vitro*. A) Upper panel. qRT-PCR of *Prdm5* levels in MC3T3 cells transduced with lentiviral shRNA constructs against *Prdm5* (shPrdm5-a and shPrdm5-b) and control construct. Lower panel. Prdm5 western blot from the same experiment. Tubulin is included as loading control. B) Upper panel. Quantification of Alizarin red staining after 21 days of osteogenic differentiation in MC3T3 cells. Data are presented as mean of 3 independent experiments ± SEM. ** = p<0.01 and *** = p<0.005 (t-test). Lower panel. Representative image from osteogenic differentiation experiments. C) Western blot showing overexpression of human PRDM5 (marked with star) in MC3T3 cells (filled circle = endogenous Prdm5). GFP overexpression is used as a negative control and Vinculin western blot for equal protein loading. D) Quantification of Alizarin red staining from osteogenic differentiation experiments of MC3T3 cells overexpressing GFP or PRDM5. A representative experiment is shown and data are presented as average ± standard deviation. E) qRT-PCR analysis of WT and *Prdm5^LacZ/LacZ^* calvaria osteoblasts for osteogenic markers as indicated. Expression values were normalized to a panel of housekeeping genes (*Rps18, Ubc, Actb, Rpl0*) and indexed to the average expression value of wild type clones. * = p<0.05 and *** = p<0.001, by unpaired T-test, +/+ (n = 14), LacZ/LacZ (n = 19).

Since Prdm5 is highly expressed in osteoblastic cells, we chose the MC3T3 cell line model to investigate the roles of *Prdm5* during osteogenic differentiation. Cells were transduced with lentiviral shRNA constructs against *Prdm5*, which resulted in efficient reduction of both *Prdm5* transcript and protein levels (Figure 2A). *Prdm5* depletion did not significantly affect proliferation of MC3T3 cells as assessed by BrdU labeling (data not shown) but led to a significant reduction in matrix mineralization as measured by Alizarin red staining of calcium nodules upon induction of osteogenic differentiation (Figure 2B). In line with this, overexpression of PRDM5 induced the opposite phenotype resulting in increased nodule formation (Figure 2C and 2D). In parallel, the significance of *Prdm5* in chondrogenesis was evaluated by knocking down *Prdm5* in the ATDC5 chondrogenic cell line (Figure S2A). In this system, Prdm5 loss did not affect chondrogenic differentiation, as evaluated by measuring glycosaminoglycan deposition (Figure S2B).

To investigate transcriptional changes imposed by Prdm5 loss in primary osteoblasts, expression levels of a series of osteogenic markers in Prdm5 wild type and mutant calvarial osteoblasts were measured by qRT-PCR. Significant reduction was observed for transcripts encoding Osteocalcin (*Bglap1*) and Bone sialoprotein (*Ibsp*), both late osteoblast markers, while transcript levels for early markers such as Osteopontin (*Spp1*), Osterix (*Sp7*) and *Runx2* were unchanged (Figure 2E). When assayed for mineralization activity, no difference in matrix calcification was detected by Alizarin red staining in calvarial osteoblasts from cohorts of WT and mutant animals (Figure S2C and S2D). However, this phenotype may depend on cellular adaptation in culture, since treatment of wild type calvarial osteoblasts with a siRNA oligo which efficiently reduces Prdm5 levels, resulted in decreased matrix mineralization after 14 days of osteogenic stimulation (Figure S2E).

Collectively, the data indicate that Prdm5 exerts a cell-autonomous function in the osteogenic pathway.

### Prdm5 targets gene bodies of transcriptionally active genes via a consensus sequence

To unveil the molecular functions of Prdm5 in osteoblastic cells and identify direct target genes, chromatin immunoprecipitation followed by deep sequencing (ChIP-seq) was performed for Prdm5 in the MC3T3 cell line.

Two different Prdm5 polyclonal antibodies were generated and western blot analysis of Prdm5 wild type and mutant mouse embryonic fibroblasts revealed that these antibodies recognize distinct epitopes (Figure S3A), and both were confirmed as suitable for ChIP experiments (Figure S3B).

**Figure 3:**
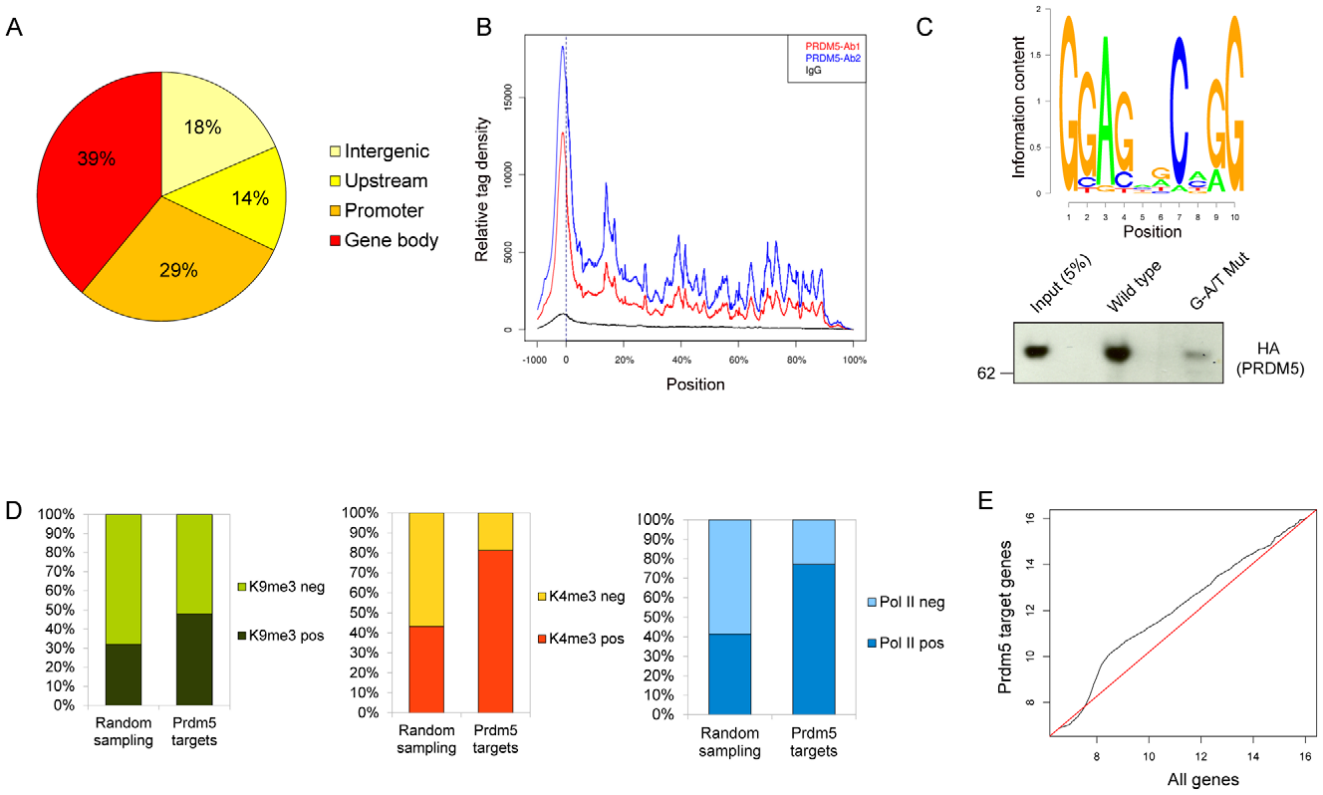
Analyses of Prdm5 chromatin interactions. A) Diagram illustrating the overall distribution of Prdm5 binding sites categorized according to the distance from the nearest TSS (see Text S1). B) The mean distribution of tags across gene bodies for Prdm5 ChIP-seq (Prdm5-ab1 in blue, Prdm5-ab2 in red and IgG in black). Vertical dashed line at x = 0 represents the TSS. Positions after the TSS are represented as % of the length of the gene. C) Upper panel. Slogos plot produced using the motifs detected by the Weeder program from Prdm5 “shrunk” peaks (see Text S1). Lower panel. DNA pulldown assay from nuclear extract of 293 cells overexpressing HA-PRDM5 using biotinylated oligos representing the *Col1a1* exon 33 (WT) and a mutated control sequence (G-A/T Mut). D) Histogram showing the percentage of H3K9me3 (left panel), H3K4me3 (middle panel) and RNA Polymerase II (right panel) positivity for “Random sampling” (mean value of 100 iterations for 1446 random genes sets) or for Prdm5 target genes. (E) Q-Q plot comparing the quantile distribution of Prdm5 target genes' expression (on Y axis) and all genes (on X axis). Red line is reference line representing equal quantile distribution.

Data from ChIP-seq analyses with the two antibodies were overlaid resulting in 1712 common loci we defined as high confidence Prdm5 target regions (Figure S3C and Table S1). Interestingly, 29% of Prdm5 peaks resided in promoter regions, while 39% of the peaks resided inside the body of genes (Figure 3A). Across all genes, Prdm5 binding was distributed throughout the length of target genes with the highest density around the TSSs (Figure 3B). A *de novo* motif finding algorithm for Prdm5 peak centers identified a putative consensus sequence for Prdm5 binding (Figure 3C). This sequence bears strong similarity to a Prdm5 consensus previously identified by *in vitro* random oligonucleotide selection experiments (Figure S3D) [4]. To confirm whether DNA fragments containing the identified sequence motif were directly recognized by Prdm5, *in vitro* pulldown assays using biotinylated DNA oligonucleotide probes were performed. Overexpressed HA-PRDM5 readily bound to DNA probes containing the consensus motif (a region of *Col1a1* exon 33, containing 3 motifs with p-score 0.96), whereas the binding was impaired by mutation of the first and last two guanines of the consensus motifs (Figure 3C).

Gene activity in MC3T3 cells was also estimated by performing ChIP-seq on the same chromatin preparation for total RNA polymerase II, histone H3 lysine 4 trimethylation (H3K4me3) and H3 lysine 9 trimethylation (H3K9me3), allowing for a correlation of Prdm5 binding to gene activity. Prdm5 bound genes were enriched either for the presence of H3K9me3 or H3K4me3, with a strong preference for Prdm5 target genes to present H3K4me3 peaks around their TSS, when compared to the average of 100 permutations of a size-matched set of random genes (Figure 3D). These results indicate that Prdm5 may act both as a transcriptional repressor and activator in a promoter-dependent fashion and that in MC3T3 cells the majority of Prdm5 target genes are actively transcribed. Indeed, Prdm5 target genes were also associated with RNA polymerase II occupancy (to a similar extent as H3K4me3) (Figure 3D) and expression analysis from microarray data of MC3T3 cells showed that genes bound by Prdm5 are characterized by a general increase in expression signal with respect to the total of the genes represented on the microarray (Figure 3E).

### Prdm5 targets genomic loci encoding collagen genes and sustains collagen I transcription in osteoblasts

**Figure 4:**
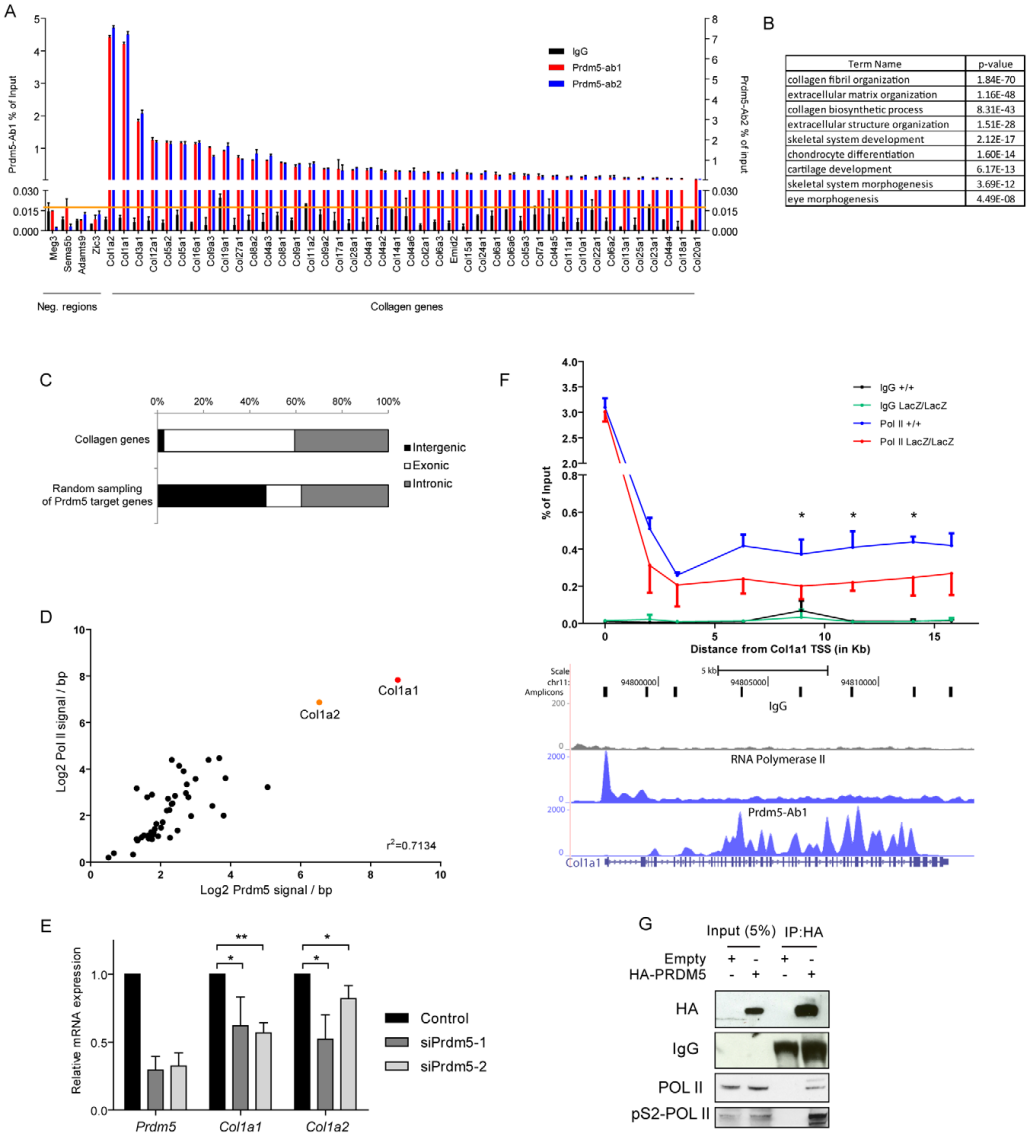
Prdm5 targets all collagen genes and regulates type I collagen expression. A) ChIP-qPCR validation of Prdm5 peaks in collagen genes or negative control regions from an independent chromatin preparation immunoprecipitated with IgG, Prdm5-Ab1 (in black and blue respectively, plotted on left Y axis) or Prdm5-Ab2 (in red, plotted on right Y axis). Orange horizontal line represents the highest “noise” value obtained by ChIP-qPCR on a set of negative regions. B) Biological processes enrichment from gene ontology annotation of Prdm5 target regions. C) Distribution of the “peak centre” position for collagen genes targeted by Prdm5 or random sampling of Prdm5 target genes according to genetic feature. D) Correlation between Prdm5-Ab1 coverage inside the gene body of all mouse collagen genes (X-axis) and Pol II coverage in the same regions (Y-axis) normalized by base pairs. E) qRT-PCR for *Prdm5*, *Col1a1* and *Col1a2* in MC3T3 cells treated for 72 hours with siRNA oligos against *Prdm5* (siPrdm5-1 and -2) or controls. Results are presented as average of four independent experiments ± SD; * = p<0.05, ** = p<0.01. F) Upper panel. ChIP-qPCR for RNA Pol II in WT and mutant (blue and red respectively) calvarial osteoblasts along the *Col1a1* gene. IgG control is represented by black and green lines respectively. X-axis = distance (in bp) from *Col1a1* TSS, * = p<0.05 (unpaired t-test). Lower panel. Genome browser snapshot of the corresponding *Col1a1* genomic region displaying MC3T3 tracks for: qPCR amplicons, IgG, RNA Pol II and Prdm5-Ab1 coverage. G) Western blot from co-immunoprecipitation experiment of HA-PRDM5 in HEK293 cells; endogenous interacting proteins or IgG are indicated.

Annotation of the Prdm5 bound loci and detailed analysis followed by ChIP-qPCR validation on independent samples with both Prdm5 antibodies strikingly revealed that 42 of 43 collagen genes in the mouse genome contained at least one Prdm5 peak (Figure 4A). Moreover, the results were validated in primary wt and LacZ/LacZ calvarial osteoblasts, where Prdm5 enrichment was strongly reduced in mutant cells (Figure S4A and S4B).

When genomic regions bound by Prdm5 were subsequently annotated according to biological processes, a strong enrichment was observed for genes associated with collagen fibril and extracellular matrix organization, as well as bone development (Figure 4B). Prdm5 binding to collagen genes occurred almost exclusively in the gene body with approx. 60% of peaks centered in exonic regions (Figure 4C). Importantly, Prdm5 occupancy in the body of collagen genes correlated with the amount of bound RNA polymerase II in the same regions (Figure 4D), suggesting a role for Prdm5 in sustaining transcriptional activity of the collagen gene family.

Very high enrichment for Prdm5 binding was observed in the two genomic loci corresponding to the collagen I genes *Col1a1* and *Col1a2* (Figure 4D and Figure S4C). Prdm5 knockdown by means of two siRNA oligos demonstrated that Prdm5 occupancy in *Col1a1* and *Col1a2* genes was functionally relevant, as reduction in Prdm5 levels led to a decrease in transcript and protein levels of both type I collagen genes (Figure 4E and Figure S4D). This effect was observed also in primary osteoblasts upon genetic ablation of Prdm5 (Figure S4E). Given the close correlation between Prdm5 occupancy and RNA polymerase II presence, we measured the occupancy of the latter along the whole length of the *Col1a1* gene in Prdm5 wild type and mutant osteoblasts. While RNA polymerase II levels were unchanged in LacZ/LacZ cells at the *Col1a1* TSS, we observed a significant drop in RNA polymerase II levels in mutant Prdm5 osteoblasts between +6.2 kb and the end of the *Col1a1* gene (Figure 4F). Towards understanding the mechanism, we hypothesized that Prdm5 could affect RNA polymerase II by direct interaction. Indeed, overexpressed HA-PRDM5 co-immunoprecipitated with endogenous RNA polymerase II and with higher affinity to the elongating form of RNA polymerase II as evident from analysis using a phospho-serine 2 specific RNA polymerase II antibody (Figure 4G). In summary, Prdm5 targets virtually all the collagen genes in the mouse genome and high Prdm5 occupancy inside the *Col1a1* gene body promotes RNA polymerase II occupancy and transcription.

### Prdm5 regulates *Decorin* via a distal element with an enhancer-like chromatin signature

**Figure 5:**
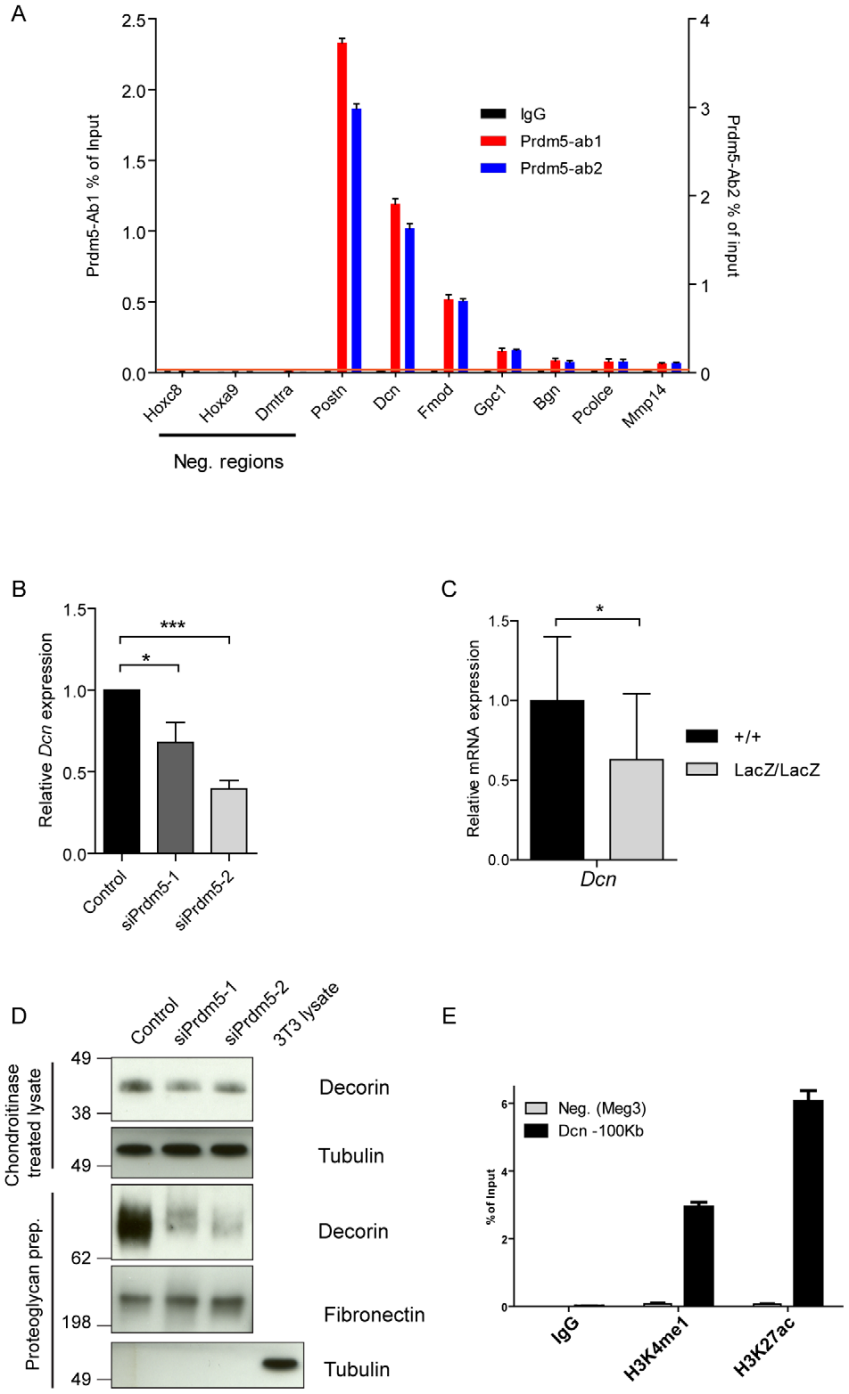
Prdm5 regulates *Decorin* through a distal enhancer. A) ChIP-qPCR validation of Prdm5 peaks on selected ECM genes or negative regions from an independent sample immunoprecipitated with IgG, Prdm5-Ab1 (in black and blue respectively, plotted on left Y axis) or Prdm5-Ab2 (in red, plotted on right Y axis). Orange horizontal line represents the highest “noise” value obtained by ChIP-qPCR on a set of negative regions. B) qRT-PCR analysis of *Decorin* transcript (*Dcn*) levels upon Prdm5 knockdown as in Figure 4E. Results are presented as average of four independent experiments +/− SD; * = p<0.05. (C) qPCR analysis of WT and *Prdm5^LacZ/LacZ^* calvarial osteoblasts for *Decorin*. Expression values were normalized to the control WT samples. *  = p<0.05; T-test, (+/+ n = 14, LacZ/LacZ n = 19 clones). D) Upper panels. Western blot analysis of Decorin levels upon Prdm5 knockdown in cell layers; Tubulin is used as loading control. Lower panels. Western blot analyses of purified proteoglycans from cell culture media from knockdown cells. Tubulin is used as purity control and Fibronectin for equal protein loading. E) ChIP-qPCR with indicated antibodies for Prdm5 binding site upstream of Dcn gene. Meg3 TSS region is used as negative control.

Analyses of the annotation of Prdm5 bound loci revealed that the Prdm5 target repertoire extends to genomic regions encoding other ECM genes involved in collagen fibrillogenesis, such as Periostin (*Postn*) and genes from the SLRP family, such as Decorin (*Dcn*), Fibromodulin (*Fmod*), Biglycan (*Bgn*) and Epiphycan (*Epc*). Also in this case, Prdm5 occupancy on selected targets was validated in independent samples from MC3T3 cells (Figure 5A), as well as primary calvarial osteoblasts (Figure S5A and S5B).

Since Decorin is well known to regulate collagen fibrillogenesis, we analyzed the influence of Prdm5 on Decorin expression. Indeed, we observed that Prdm5 knockdown resulted in decreased *Decorin* transcription (Figure 5B). This effect could be reproduced in Prdm5*^LacZ/LacZ^* calvarial osteoblasts (Figure 5C). While cell-associated Decorin protein levels were only mildly decreased upon Prdm5 knockdown (Figure 5D), the amount of secreted Decorin detected in cell culture media from Prdm5 siRNA treated cells was strongly reduced (Figure 5D). A closer inspection revealed that the Prdm5 binding site assigned to *Decorin* gene was 100 kilobases distant from its TSS, suggesting the binding to a distal enhancer (Figure S5C). Chromatin immunoprecipitation for H3K4me1 and H3K27ac confirmed that Prdm5 bound a site upstream of *Dcn* with a chromatin signature corresponding to an enhancer element (Figure 5E). Our data show that Prdm5 targets SLRP family members and likely regulates Decorin via a distal enhancer.

### Delayed ossification and impairment of collagen fibrillogenesis in *Prdm5^LacZ/LacZ^* embryos

**Figure 6:**
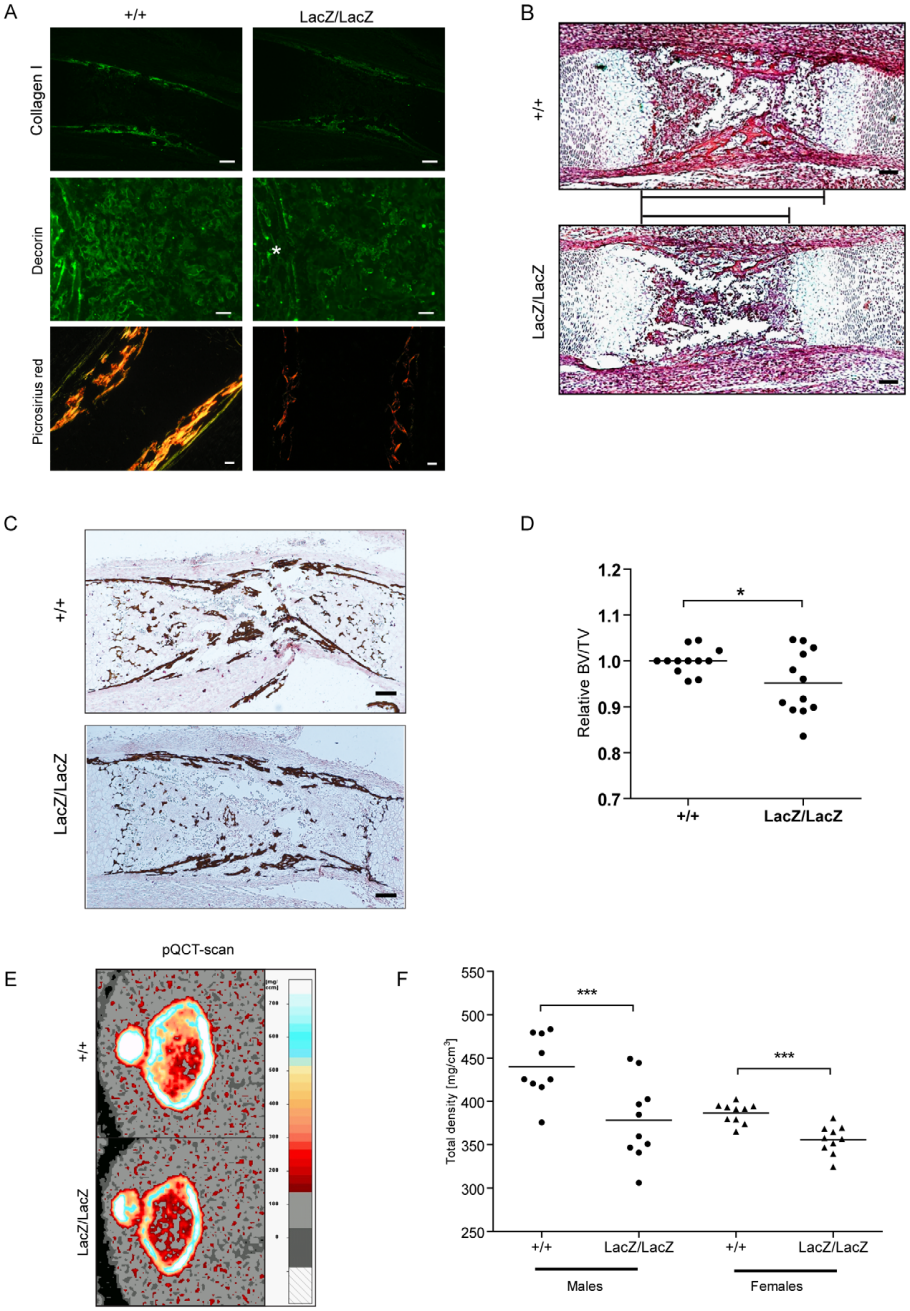
Prdm5 loss results in decreased Collagen I and Decorin levels and leads to reduced bone formation *in vivo*. A) Immunofluorescence staining of E16.5 tibiae for Collagen I (upper panel, bar = 200 µm), Decorin (middle panel, bar = 100 µm, asterisk indicates periosteum area) and Picrosirius red staining of WT and *Prdm5^LacZ/LacZ^* E16.5 tibiae under polarized light (lower panel, bar = 100 µm). B) H&E staining of *Prdm5* WT and *Prdm5^LacZ/LacZ^* E16.5 bones. The bars indicate the length of the osteoblast region (bar = 100 µm). C) Von Kossa staining of WT and *Prdm5^LacZ/LacZ^* E16.5 tibiae. D) μCT measurements of E18.5 embryos. BV/TV = Bone volume/Total embryo volume. Values for *Prdm5^LacZ/LacZ^* embryos are normalized to littermate controls. * = p<0.05; T-test with Welch correction. E) Representative pictures from pQCT scans of femoral metaphysis of a 5 weeks old *Prdm5^LacZ/LacZ^* mouse and a littermate control. Color bar represents the density scale. F) Quantification of total bone mineral density in femoral metaphysis from 5 weeks old WT and *Prdm5^LacZ/LacZ^* animals. Each dot represents the average of two measurements on each animal tested (n = 9/10 per group).

Collagen I is the main component of osteoblastic ECM [11] and Decorin is known to regulate collagen I fibrillogenesis [16]. The observed Prdm5 regulation of Collagen I and Decorin genes prompted us to characterize their deregulation *in vivo* and the resulting phenotype in *Prdm5^LacZ/LacZ^* mice. qRT-PCR analyses revealed that the *Col1a1* and *Col1a2* transcripts were significantly reduced upon Prdm5 loss in E16.5 limbs (Figure S6A). Moreover, decreased Collagen I was observed by *in situ* hybridization in the periosteum at E16.5 (data not shown), as well as by immunofluorescence microscopy (Figure 6A). While *Decorin* transcript levels were unchanged in whole E16.5 mutant limbs (Figure S6B), immunofluorescence staining displayed reduction of Decorin protein, particularly in the periosteum and invading osteoblasts region (Figure 6A), indicating an osteoblast-restricted Prdm5 regulation of Decorin. Given that both molecules are involved in collagen fiber formation, fibrillar collagen levels were evaluated in *Prdm5^LacZ/LacZ^* embryonic limbs by picrosirius red staining. Using bright field microscopy, a decreased staining of collagen could be appreciated in the mutants (Figure S6C). Moreover, using polarized light to visualize specifically assembled collagen fiber birefringency, a marked decrease in the presence and organization of collagen fibers was observed in *Prdm5^LacZ/LacZ^* embryonic limbs (Figure 6A).

Histological examination revealed a shorter osteoblast compartment in mutant animals (Figure 6B), suggesting a delay in the ossification process. Likewise, Von Kossa stainings revealed decreased calcification in mutant limbs further pointing to an impaired ossification process in *Prdm5* mutants (Figure 6C). To quantitate the delayed ossification, wild type and *Prdm5^LacZ/LacZ^* E18.5 embryos were analyzed by micro-CT (μCT) scanning. Quantification of total bone volume demonstrated a significant reduction in the ossification process in mutant embryos (Figure 6D). However, at E16.5, no overt differences in the expression of various bone formation markers were observed (Figure S6D). To measure the impact of the embryonic ossification defect in young mice hind limbs from cohorts of WT and *Prdm5^LacZ/LacZ^* mice of both genders were analyzed by peripheral Quantitative Computed Tomography (pQCT). Images of distal metaphyseal sections from CT-scans of femurs at 5 weeks of age demonstrated a decrease in bone mineral density (Figure 6E). Quantification of total bone mineral density, total mineral content and total bone area in large cohorts of mice demonstrated a statistically significant reduction in all three parameters in *Prdm5^LacZ/LacZ^* mice of both genders (Figure 6F and Table S2). Separate measurements of the cortical and trabecular compartments revealed a more robust reduction in all measured parameters in cortical regions than trabecular areas (Table S2), coinciding with the areas where downregulation of Collagen I and Decorin was predominantly observed in mutant embryo limbs.

In summary, *Prdm5^LacZ/LacZ^* animals display a significant reduction in collagen levels and fibrillogenesis, likely resulting in the observed osteopenic phenotype.

## Discussion

In this study, we uncover a novel function for Prdm5 in promoting the transcription of key extracellular matrix genes in osteoblastic cells. We find that Prdm5 is specifically expressed in the osteoblastic compartment of developing bones and exerts its function along the osteogenic lineage by promoting osteogenic differentiation in culture. Mechanistically, we demonstrate that Prdm5 targets ECM gene families such as collagens and small leucine-rich proteoglycans. By association with RNA polymerase II, Prdm5 sustains the transcription of collagen I genes, while the regulation of Decorin expression is mediated via binding to a distal enhancer-like element. Prdm5 regulation of these genes occurs *in vivo* in developing limbs resulting in the decreased bone mineral density observed in *Prdm5^LacZ/LacZ^* animals.

Members of the PRDM family display tissue specific patterns of expression [24], in agreement with a role in tissue specific differentiation. In line with this, Prdm1 is known to be a master regulator of lymphoid differentiation [2], Prdm16 to control brown fat development [3], Prdm14 to regulate embryonic stem cell pluripotency and germ cell differentiation [25], [26] and Prdm9 to determine meiotic recombination hotspots [27]. So far *Prdm5* has not been characterized in the context of mammalian development. Our study thus provides the first evidence of a role for Prdm5 in tissue specific differentiation extending the concept of PRDM proteins as regulators of specific tissues and in agreement with the idea of functional specialization of PRDM members during expansion of the family in vertebrates [28].

The identification of high Prdm5 expression in osteoblasts and its function in osteogenic differentiation *in vitro* permitted us to evaluate its molecular functions in a relevant cellular context. Indeed, it is noteworthy that Prdm5 silencing does not restrict chondrogenic differentiation *in vitro* suggesting cell type specificity in Prdm5 action.

By genome wide mapping of Prdm5 binding sites in a pre-osteoblastic cell line, we demonstrate a unique capacity of Prdm5 to bind the whole family of collagen genes and especially to bind these genes within the gene body. This is an unprecedented feature for a transcriptional regulator, i.e. binding the gene bodies of all the members of such a large gene family. Moreover, we observed Prdm5 occupancy in genomic regions encoding for non-collagen proteins with collagenous domains (e.g. C1q family), suggesting that either Prdm5 consensus sequence is overrepresented in genomic regions encoding for collagenous domains, or that simply Prdm5 binds common regulatory elements shared by specific gene families. Indeed we detected Prdm5 binding to several members of the SLRP family as well as a number of genes encoding for essential extracellular matrix components (complete list in Table S1), demonstrating that Prdm5 potentially regulates a wide but specific transcriptional program necessary for proper extracellular matrix formation.

In this study, we focus on fibrillar collagens, particularly Collagen I genes; these were highly enriched for Prdm5 binding and are essential constituent of osteoblastic matrix. A number of transcription factors have been shown to regulate Collagen I genes and the location of their binding sites on *Col1a1* and *Col1a2* promoters have been shown to determine the expression in different osteoblast subtypes [29], [30]. Very little is known concerning the regulation of *Col1a1* and *Col1a2* gene expression by factors binding downstream of their promoter, except for a repressor region located within intron 1 that can recruit GATA and IRF proteins to block enhancer stimulation of promoter activity [31], [32]. To our knowledge, Prdm5 is the first example of a transcriptional modulator able to bind a consensus sequence found inside collagen genes, particularly *Col1a1* and *Col1a2*.

Interestingly, upon Prdm5 loss in osteoblasts, we observe decreased RNA polymerase II occupancy throughout the gene body of the *Col1a1* gene, while at the TSS, RNA polymerase II occupancy remains unchanged. This suggests that Prdm5 is sustaining transcription of the *Col1a1* gene by affecting polymerase processivity. This hypothesis is further supported by the observed interaction between Prdm5 and RNA polymerase II. The higher enrichment for the processive, “elongating”, form of RNA polymerase II phosphorylated on Serine 2 of its C-terminal domain, argues in favor of Prdm5 directly sustaining RNA polymerase II processivity although further analyses are required to clearly define the underlying mechanism. Since Prdm5 binds predominantly exonic regions of collagen genes, and splicing is known to occur co-transcriptionally [33], there may be a role for Prdm5 in the coupling of splicing to transcription. However, we failed to detect an altered *Col1a1* splice pattern in Prdm5 mutant osteoblasts (data not shown), making this hypothesis less likely. The correlation between Prdm5 binding levels within collagen genes and RNA polymerase II occupancy suggests that Prdm5 in certain contexts may act as a transcriptional activator in contrast to the previously described role of PRDM5 acting predominantly as a transcriptional repressor [4]. Interestingly, we observe major enrichment for Prdm5 target genes to be transcriptionally active, presenting both H3K4me3 and RNA Polymerase II (Figure 3D), suggesting a predominant role for Prdm5 in transcriptional activation in osteoblastic cells.

The Prdm5 target repertoire extends also to SLRP genes, including Decorin (*Dcn*), Fibromodulin (*Fmod*), Biglycan (*Bgn*) and Epiphycan (*Epc*). Members of this family are involved in correct type I collagen fibers assembly [15]. Specifically, we found a Prdm5 binding site residing 100 kb upstream from the *Dcn* TSS. *Decorin* is the closest gene within a range of 760 kb and the Prdm5 binding site displays the chromatin signature of an enhancer element, suggesting a previously undiscovered role for Prdm5 in transcriptional enhancement or chromatin organization. Decorin has been shown to bind directly collagen I [34] and, in bone tissues, mice lacking Decorin display decreased collagen fiber diameter, although they do not show pronounced skeletal defects [35]. Our data show a marked decrease in fibrillar collagen staining, as evaluated by collagen fibers birefringency, indicating that downregulation of both Collagen I transcripts as well as *Decorin* might contribute to the skeletal phenotype observed.

Of direct relevance, mutations in *PRDM5* were recently detected in Brittle Cornea Syndrome [9]. Brittle Cornea Syndrome is a generalized connective tissue disease characterized by impairment of ECM formation and patients develop a number of other symptoms, aside from ocular defects, such as skin hyperelasticity, joint hypermobility and skeletal defects [10], [36]. While no studies so far have causally linked PRDM5 to this disease, our data demonstrating that Prdm5 regulates the expression of fibrillar collagens are in line with the defects in ECM production and assembly characteristically observed in BCS patients. The distinct expression pattern observed for *Prdm5* in developing skeleton prompted us to evaluate a bone phenotype but future studies will be needed to understand if *Prdm5^LacZ/LacZ^* mice develop also corneal, skin and joint defects resembling BCS or related diseases such as Ehlers-Danlos syndrome. In this regard, considering the capability of Prdm5 to bind genomic loci encoding multiple collagen molecules, it may be that Prdm5 affects the levels of specific collagen molecules in a context-specific fashion. These features support the role for Prdm5 as an important regulator of extracellular matrix genes transcription during the process of bone formation, and extend the need for studies characterizing this protein in different clinical settings.

## Materials and Methods

### Animal experiments

To generate the *Prdm5^LacZ^* strain, ES cell clone AV0702 from the Sanger Institute Gene Trap project was microinjected into C57BL/6 blastocysts. The integration site of the β-geo cassette was established using long range PCR with primers spanning different regions of intron 2 and a genotyping strategy was devised accordingly. All experiments were performed on mice backcrossed for at least 6 generations into the C57BL/6 strain and comparisons between WT and mutant animals always refer to littermates. All animal experimentation was performed with approval from Danish authorities (Dyreforsøgstilsynet, protocol number 2006/562-43) and the Regierung von Oberbayern (Government of Upper Bavaria).

### Histological staining and immunohistochemistry

For whole mount X-gal staining, embryos were deskinned (after E15) and fixed in 0.25% glutaraldehyde (Sigma). Stainings were performed by incubation in staining solution containing 0.02% NP40, 0.01% Sodium Deoxycholic acid, 2 mM MgCl_2_, 20 mM Tris-HCl (pH 7.4), 50 µM K-Ferrocyanide, 50 µM K-Ferricyanide, 1 mg/mL X-gal in PBS (chemicals purchased from Sigma). Stained embryos were post-fixed in 4% paraformaldehyde and cleared in increasing concentrations of glycerol in 1% KOH. Images were acquired with a Leica D-Lux 3 on Leica S6D stereoscope. Von Kossa staining was performed by staining dehydrated sections in 1% AgNO_3_ under UV light, followed by incubation with 5% Sodium thiosulfate. Picrosirius red stainings were performed as described [37]. Images were acquired under polarized light using a BX51 Olympus microscope. In-situ hybridization was performed as previously described [38] and protocols for immunofluorescence stainings are described in Text S1.

### Cell culture and transfections

Primary human osteoblasts (kindly provided by Bente Langdahl, Århus University, Denmark) were isolated from bone biopsies of healthy donors. MC3T3 cells were maintained in Alpha-MEM (Gibco) with 10% FBS (Hyclone) and 1% penicillin-streptomycin (Gibco). ATDC5 cells were maintained in DMEM∶F12 (Gibco) media supplemented with 5% FBS, 10 µg/ml human transferrin (Roche) and 30 nM sodium selenite (Sigma). Calvarial osteoblasts were derived as previously described [21]. Differentiation protocols and transfection/transduction techniques are detailed in Text S1.

### Co-immunoprecipitation, immunoblotting, and gene expression analyses

For co-immunoprecipitation experiments HEK293 cells were transfected with indicated plasmids and, 48 hours after, lysed in ELB buffer (150 mM NaCl, 50 mM HEPES, 0.1% Igepal) and incubated with HA-agarose beads (Sigma) overnight. After washes, proteins were recovered by boiling beads in SDS Laemmli buffer.

For whole cells lysate immunoblotting, cells were harvested and lysed in RIPA buffer and subjected to standard SDS-PAGE. Protocols and antibodies used are detailed in Text S1.

Total RNA was extracted from cell pellets using the RNeasy Plus Mini Kit (Qiagen) according to the manufacturer's instructions. cDNA was synthetized using TaqMan Reverse Trancription Reagents (Applied Biosystems). qRT-PCR was performed with the One Step plus Sequence Detection System (Applied Biosystems) using Fast SYBR green master mix reagent (Applied Biosystems). Gene expression levels were normalized to the average of at least two housekeeping genes. qPCR primers sequences are listed in Table S3. For MC3T3 microarray data, RNA was amplified and labeled using TotalPrep RNA Amplification Kit (Ambion) and hybridized to MouseWG-6 v2.0 Expression BeadChip array (Illumina) according to manufacturer's recommendation.

### Chromatin immunoprecipitation and Deep sequencing

Chromatin immunoprecipitation assay (ChIP) protocol and antibodies used are detailed in Text S1. Libraries for sequencing were obtained using the ChIP-seq DNA sample prep kit (Illumina) according to manufacturer recommendations and samples were sequenced on a Genome Analyzer II sequencer (Illumina). Primers for ChIP-qPCR experiments are listed in Table S3.

### Bioinformatic analysis

Microarray and ChIP-seq analyses are detailed in Text S1.

### μCT and Peripheral quantitative computed tomography

E18.5 embryos were scanned using a micro CT scanner (model 1172, Skyscan, Belgium) at 50 kV, 200 µA and a 0.5 mm aluminium filter. Adult mice at 5 weeks of age were analyzed by pQCT using Stratec XCT Research SA+ (Stratec Medizintechnik GmbH, Germany) at the German Mouse Clinic [39]. Analyses details are provided in Text S1.

## Supporting Information

Figure S1 *Prdm5^LacZ/LacZ^* mice do not display gross abnormalities and PRDM5 is expressed in osteoblasts. A) Left panel. Western blot of +/+ and LacZ/LacZ mouse embryo fibroblasts using serum from mice immunized with recombinant Prdm5^1-142^. Right panel. qPCR for *Prdm5* wt and *Prdm5-βgeo* fusion alleles in mouse embryo fibroblasts of the three genotypes. B) qPCR for *Prdm5* wild type transcript in a panel of tissues from wild type and *Prdm5^LacZ/LacZ^* mice. Results are shown as the average of 3 animals per group ± standard deviations. C) Number of mice obtained at weaning with different genotypes from *Prdm5^+/LacZ^* intercrosses. In parenthesis the expected numbers according to Mendelian ratios. Statistical differences were calculated by Chi-square test. D) Weight of wild type and *Prdm5^LacZ/LacZ^* mice up to 56 weeks of age. E) qPCR analysis for *PRDM5* in primary human osteoblasts from 6 different donors (black bars) and a panel of human immortalized cell lines of different origins (grey bars). F) Western blot analysis of Prdm5 protein expression in the indicated mouse cell lines. * = unspecific bands for equal loading.
(PDF)

Figure S2 Prdm5 knockdown affects osteogenic differentiation in vitro, but not chondrogenic differentiation. A) Left panel. qPCR of *Prdm5* levels in chondrogenic ATDC5 cells infected with lentiviral shRNA constructs against the *Prdm5* transcript (shPrdm5-a) and control construct. Right panel. Western blot measuring Prdm5 protein levels in the same cells. Actin is used as a control for equal protein loading. B) Upper panel. Quantification of glycosaminoglycans deposition from ATDC5 cells infected with control or shPrdm5-a constructs after 17 days of stimulation with chondrogenic media and stained with Alcian blue. Data are presented as the mean of 2 independent experiments ± standard deviation. Lower panel. Representative picture from the chondrogenic differentiation experiment. C) Representative pictures of alizarin red stained WT and *Prdm5^LacZ/LacZ^* calvarial osteoblasts after 14 days of osteogenic differentiation. D) Quantification of the alizarin red staining from osteogenic differentiation experiments in WT and *Prdm5^LacZ/LacZ^* calvarial osteoblasts. Data are presented as mean ± standard deviation for 10 different littermate primary cultures per group. E) Left panel. Western blot measuring Prdm5 protein levels in primary calvarial osteoblasts treated for 72 hours with control oligo or siRNA pool against *Prdm5* (siPrdm5). Vinculin is used as a control for equal protein loading. Right panel. Quantification of calcified matrix deposition from calvarial osteoblasts treated with control or siPrdm5 after 14 days of stimulation with osteogenic media and stained with Alizarin red. Data are presented as the mean of 4 independent experiments ± standard deviation. Lower panel. Representative picture from the osteogenic differentiation experiment.
(PDF)

Figure S3 Validation of Prdm5 ChIP-grade antibodies. A) Western blot of +/+ and LacZ/LacZ mouse embryo fibroblasts using Prdm5 rabbit polyclonal antibodies Prdm5-ab1 and Prdm5-ab2. B) Left panel. Transfection control of HEK293 cells containing a stably integrated GAL4TkLuc reporter with empty vector or vector expressing Prdm5 fused to Gal4 DNA binding domain (Gal4BD-Prdm5 OE). Tubulin is used for equal protein loading. Right panel. Chromatin immunoprecipitation from Gal4BD-Prdm5-OE sample with IgG (negative control), Gal4 (positive control) and a range of in house-generated or commercially available Prdm5 antibodies. C) Venn diagram of the overlay of peaks identified by ChIP-seq in MC3T3 in experiment either with Prdm5-ab1 or Prdm5-ab2. D) Slogo representation of re-analysis of sequences retrieved by previously published random oligonucleotide experiment [4].
(PDF)

Figure S4 Prdm5 binds inside *Col1a1* and *Col1a2* gene bodies and regulates their transcription. A) ChIP-qPCR in primary calvarial osteoblasts with IgG or Prdm5-ab1 (black and red bars respectively) for Prdm5 binding site in selected collagen genes. Genomic regions around *Meg3* and *Sema5b* are used as negative controls. Orange horizontal line represents the highest “noise” value obtained by ChIP-qPCR on a set of negative regions. B) ChIP-qPCR in wild type (red bars) and *Prdm5^LacZ/LacZ^* (blue bars) calvarial osteoblasts for a number of collagen genes. Genomic regions around *Meg3* and *Sema5b* are used as negative controls. Orange horizontal line represents the highest “noise” value obtained by ChIP-qPCR on a set of negative regions. C) Genome browser snapshot for *Col1a1* (upper panel) and *Col1a2* (lower panel) regions. Tracks represent IgG, Prdm5-Ab1 and Prdm5-Ab2 sequencing results as indicated. D) Western blot for Prdm5 and Collagen I levels from MC3T3 cells treated for 72 hours with control or Prdm5 siRNA oligos. Tubulin is used for equal protein loading. E) qPCR analysis of WT and *Prdm5^LacZ/LacZ^* calvarial osteoblasts for *Col1a1*and *Col1a2*. Expression values were normalized to WT controls. *** = p<0.001 according to T-test, (+/+ n = 14, LacZ/LacZ n = 19 clones).
(PDF)

Figure S5 Prdm5 binds a distal element from *Dcn* gene TSS in calvarial osteoblasts. A) ChIP-qPCR in primary calvarial osteoblasts with IgG or Prdm5-ab1 (black and red bars respectively) for Prdm5 binding site assigned to ECM genes identified from ChIP-seq. Genomic region around *AdamTS9* is used as negative control. Orange horizontal line represents the highest “noise” value obtained by ChIP-qPCR on a set of negative regions. B) ChIP-qPCR in wild type (red bars) and *Prdm5^LacZ/LacZ^* (blue bars) calvarial osteoblasts for the main peaks assigned to ECM genes identified from ChIP-seq. Genomic region around *AdamTS9* is used as negative control. Orange horizontal line represents the highest “noise” value obtained by ChIP-qPCR on a set of negative regions. C) Genome browser snapshot for Prdm5 target region assigned to *Dcn* gene. Tracks represent IgG, Prdm5-Ab1 and Prdm5-Ab2 sequencing results as indicated.
(PDF)

Figure S6 Characterization of Prdm5 target genes levels and osteoblasts markers in *Prdm5^LacZ/LacZ^* E16.5 limbs. A) qPCR analysis for *Col1a1* and *Col1a2* in E16.5 wild type and *Prdm5^LacZ/LacZ^* limbs (n = 4). * = p<0.05. B) qPCR analysis for *Dcn* in E16.5 WT and *Prdm5^LacZ/LacZ^* limbs (n = 4). C) Bright field image of picrosirius red staining in wild type and *Prdm5^LacZ/LacZ^* E16.5 tibia. D) In-situ hybridizations for *Spp1/Osteopontin* and *Ibsp* (bar = 400 um) and immunofluorescence micrographs of Mmp13 (bar = 100 µm), Vegfa and Osterix (bar = 200 µm) in WT and *Prdm5^LacZ/LacZ^* E16.5 embryo femurs or tibiae. White arrow indicates growth plate direction for orientation. All histological analyses are performed on parallel sections between littermate embryos of same sex.
(PDF)

Table S1 Genomic regions identified as high confidence Prdm5 targets from ChIP-seq experiment. Assigned ensembl gene IDs by annotation are indicated.
(PDF)

Table S2 pQCT parameters measured in wild type and *Prdm5^LacZ/LacZ^* animals at 5 weeks. P-value by T-test is indicated and cells color-coded according to: yellow = p<0.05, orange = p<0.01, red = p<0.005.
(PDF)

Table S3 Primers used for qPCR and ChIP-qPCR throughout the study.
(PDF)

Text S1 Extended description of experimental procedures including bioinformatics analyses and supplemental references.
(DOCX)

## References

[1] Fog CK, Galli GG, Lund AH (2011). PRDM proteins: Important players in differentiation and disease.. Bioessays.

[2] Turner CA, Mack DH, Davis MM (1994). Blimp-1, a novel zinc finger-containing protein that can drive the maturation of B lymphocytes into immunoglobulin-secreting cells.. Cell.

[3] Seale P, Bjork B, Yang W, Kajimura S, Chin S (2008). PRDM16 controls a brown fat/skeletal muscle switch.. Nature.

[4] Duan Z, Person RE, Lee HH, Huang S, Donadieu J (2007). Epigenetic regulation of protein-coding and microRNA genes by the Gfi1-interacting tumor suppressor PRDM5.. Mol Cell Biol.

[5] Deng Q, Huang S (2004). PRDM5 is silenced in human cancers and has growth suppressive activities.. Oncogene.

[6] Watanabe Y, Kim HS, Castoro RJ, Chung W, Estecio MR (2009). Sensitive and specific detection of early gastric cancer with DNA methylation analysis of gastric washes.. Gastroenterology.

[7] Watanabe Y, Toyota M, Kondo Y, Suzuki H, Imai T (2007). PRDM5 identified as a target of epigenetic silencing in colorectal and gastric cancer.. Clin Cancer Res.

[8] Meani N, Pezzimenti F, Deflorian G, Mione M, Alcalay M (2009). The tumor suppressor PRDM5 regulates Wnt signaling at early stages of zebrafish development.. PLoS ONE.

[9] Burkitt Wright EM, Spencer HL, Daly SB, Manson FD, Zeef LA (2011). Mutations in PRDM5 in brittle cornea syndrome identify a pathway regulating extracellular matrix development and maintenance.. Am J Hum Genet.

[10] Ticho U, Ivry M, Merin S (1980). Brittle cornea, blue sclera, and red hair syndrome (the brittle cornea syndrome).. Br J Ophthalmol.

[11] Schenk RK, Hofstetter W, Felix R (2003). Morphology and Chemical Composition of Connective Tissue: Bone.

[12] Olsen BR, Reginato AM, Wang W (2000). Bone development.. Annu Rev Cell Dev Biol.

[13] Vuorio E, de Crombrugghe B (1990). The family of collagen genes.. Annu Rev Biochem.

[14] Canty EG, Kadler KE (2005). Procollagen trafficking, processing and fibrillogenesis.. J Cell Sci.

[15] Kalamajski S, Oldberg A (2010). The role of small leucine-rich proteoglycans in collagen fibrillogenesis.. Matrix Biol.

[16] Danielson KG, Baribault H, Holmes DF, Graham H, Kadler KE (1997). Targeted disruption of decorin leads to abnormal collagen fibril morphology and skin fragility.. J Cell Biol.

[17] de Crombrugghe B, Vuorio T, Karsenty G, Maity S, Rutheshouser EC (1991). Transcriptional control mechanisms for the expression of type I collagen genes.. Ann Rheum Dis.

[18] Karsenty G, Park RW (1995). Regulation of type I collagen genes expression.. Int Rev Immunol.

[19] Inagaki Y, Truter S, Ramirez F (1994). Transforming growth factor-beta stimulates alpha 2(I) collagen gene expression through a cis-acting element that contains an Sp1-binding site.. J Biol Chem.

[20] Greenwel P, Tanaka S, Penkov D, Zhang W, Olive M (2000). Tumor necrosis factor alpha inhibits type I collagen synthesis through repressive CCAAT/enhancer-binding proteins.. Mol Cell Biol.

[21] Bozec A, Bakiri L, Jimenez M, Schinke T, Amling M (2010). Fra-2/AP-1 controls bone formation by regulating osteoblast differentiation and collagen production.. J Cell Biol.

[22] Komori T, Yagi H, Nomura S, Yamaguchi A, Sasaki K (1997). Targeted disruption of Cbfa1 results in a complete lack of bone formation owing to maturational arrest of osteoblasts.. Cell.

[23] Komori T (2010). Regulation of bone development and extracellular matrix protein genes by RUNX2.. Cell Tissue Res.

[24] Kinameri E, Inoue T, Aruga J, Imayoshi I, Kageyama R (2008). Prdm proto-oncogene transcription factor family expression and interaction with the Notch-Hes pathway in mouse neurogenesis.. PLoS ONE.

[25] Yamaji M, Seki Y, Kurimoto K, Yabuta Y, Yuasa M (2008). Critical function of Prdm14 for the establishment of the germ cell lineage in mice.. Nat Genet.

[26] Chia NY, Chan YS, Feng B, Lu X, Orlov YL (2010). A genome-wide RNAi screen reveals determinants of human embryonic stem cell identity.. Nature.

[27] Baudat F, Buard J, Grey C, Fledel-Alon A, Ober C (2010). PRDM9 is a major determinant of meiotic recombination hotspots in humans and mice.. Science.

[28] Fumasoni I, Meani N, Rambaldi D, Scafetta G, Alcalay M (2007). Family expansion and gene rearrangements contributed to the functional specialization of PRDM genes in vertebrates.. BMC Evol Biol.

[29] Maes C, Kobayashi T, Selig MK, Torrekens S, Roth SI (2011). Osteoblast precursors, but not mature osteoblasts, move into developing and fractured bones along with invading blood vessels.. Dev Cell.

[30] Ramirez F, Di Liberto M (1990). Complex and diversified regulatory programs control the expression of vertebrate collagen genes.. Faseb J.

[31] Antoniv TT, Tanaka S, Sudan B, De Val S, Liu K (2005). Identification of a repressor in the first intron of the human alpha2(I) collagen gene (COL1A2).. J Biol Chem.

[32] Tanaka S, Ramirez F (2007). The first intron of the human alpha2(I) collagen gene (COL1A2) contains a novel interferon-gamma responsive element.. Matrix Biol.

[33] Reed R (2003). Coupling transcription, splicing and mRNA export.. Curr Opin Cell Biol.

[34] Keene DR, San Antonio JD, Mayne R, McQuillan DJ, Sarris G (2000). Decorin binds near the C terminus of type I collagen.. J Biol Chem.

[35] Corsi A, Xu T, Chen XD, Boyde A, Liang J (2002). Phenotypic effects of biglycan deficiency are linked to collagen fibril abnormalities, are synergized by decorin deficiency, and mimic Ehlers-Danlos-like changes in bone and other connective tissues.. J Bone Miner Res.

[36] Al-Hussain H, Zeisberger SM, Huber PR, Giunta C, Steinmann B (2004). Brittle cornea syndrome and its delineation from the kyphoscoliotic type of Ehlers-Danlos syndrome (EDS VI): report on 23 patients and review of the literature.. Am J Med Genet A.

[37] Junqueira LC, Bignolas G, Brentani RR (1979). Picrosirius staining plus polarization microscopy, a specific method for collagen detection in tissue sections.. Histochem J.

[38] Wuelling M, Kaiser FJ, Buelens LA, Braunholz D, Shivdasani RA (2009). Trps1, a regulator of chondrocyte proliferation and differentiation, interacts with the activator form of Gli3.. Dev Biol.

[39] Gailus-Durner V, Fuchs H, Adler T, Aguilar Pimentel A, Becker L (2009). Systemic first-line phenotyping.. Methods Mol Biol.

