# Supplementary figures and images for: Prdm5 Regulates Collagen Gene Transcription by Association with RNA Polymerase II in Developing Bone
Source: PLoS Genet. 2012 May 10;8(5):e1002711. doi: 10.1371/journal.pgen.1002711 (PMC3349747; doi:10.1371/journal.pgen.1002711)

Galli\_Fig S1

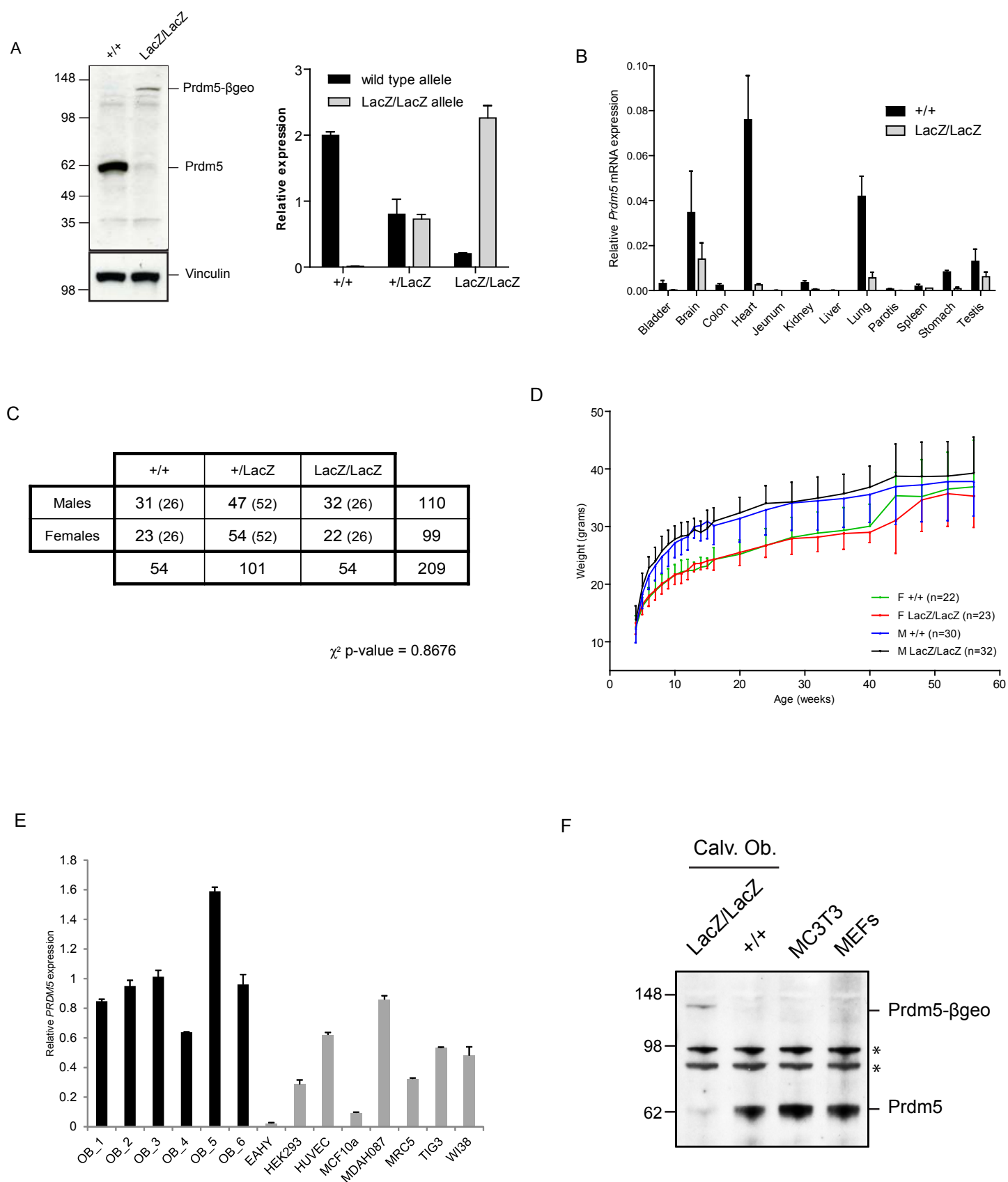

Supplement: Figure S1 — Prdm5LacZ/LacZ mice do not display gross abnormalities and PRDM5 is expressed in osteoblasts. A) Left panel. Western blot of +/+ and LacZ/LacZ mouse embryo fibroblasts using serum from mice immunized with recombinant Prdm51-142. Right panel. qPCR for Prdm5 wt and Prdm5-βgeo fusion alleles in mouse embryo fibroblasts of the three genotypes. B) qPCR for Prdm5 wild type transcript in a panel of tissues from wild type and Prdm5LacZ/LacZ mice. Results are shown as the average of 3 animals per group ± standard deviations. C) Number of mice obtained at weaning with different genotypes from Prdm5+/LacZ intercrosses. In parenthesis the expected numbers according to Mendelian ratios. Statistical differences were calculated by Chi-square test. D) Weight of wild type and Prdm5LacZ/LacZ mice up to 56 weeks of age. E) qPCR analysis for PRDM5 in primary human osteoblasts from 6 different donors (black bars) and a panel of human immortalized cell lines of different origins (grey bars). F) Western blot analysis of Prdm5 protein expression in the indicated mouse cell lines. * = unspecific bands for equal loading. (PDF) [file pgen.1002711.s001.pdf]

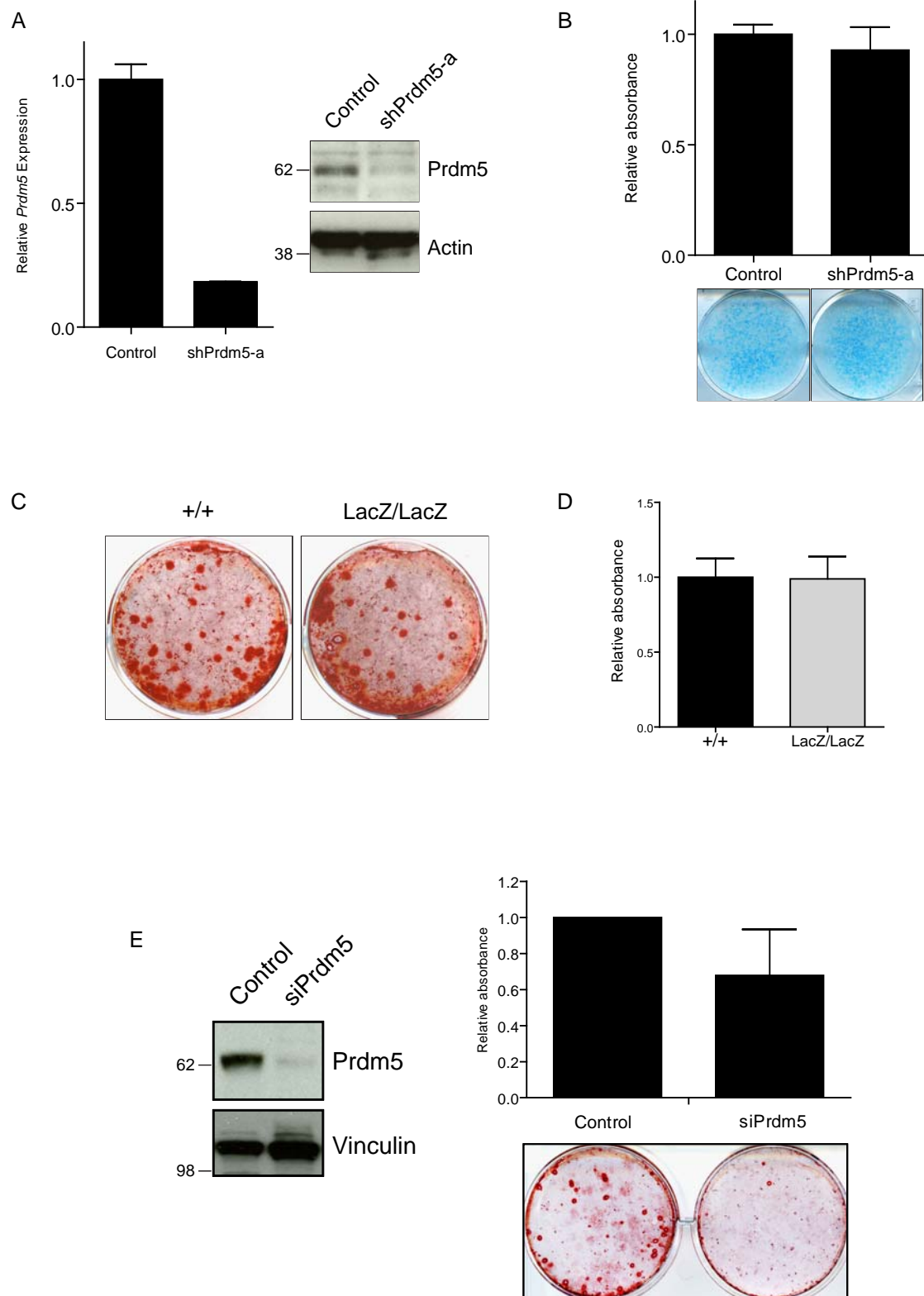

Supplement: Figure S2 — Prdm5 knockdown affects osteogenic differentiation in vitro, but not chondrogenic differentiation. A) Left panel. qPCR of Prdm5 levels in chondrogenic ATDC5 cells infected with lentiviral shRNA constructs against the Prdm5 transcript (shPrdm5-a) and control construct. Right panel. Western blot measuring Prdm5 protein levels in the same cells. Actin is used as a control for equal protein loading. B) Upper panel. Quantification of glycosaminoglycans deposition from ATDC5 cells infected with control or shPrdm5-a constructs after 17 days of stimulation with chondrogenic media and stained with Alcian blue. Data are presented as the mean of 2 independent experiments ± standard deviation. Lower panel. Representative picture from the chondrogenic differentiation experiment. C) Representative pictures of alizarin red stained WT and Prdm5LacZ/LacZ calvarial osteoblasts after 14 days of osteogenic differentiation. D) Quantification of the alizarin red staining from osteogenic differentiation experiments in WT and Prdm5LacZ/LacZ calvarial osteoblasts. Data are presented as mean ± standard deviation for 10 different littermate primary cultures per group. E) Left panel. Western blot measuring Prdm5 protein levels in primary calvarial osteoblasts treated for 72 hours with control oligo or siRNA pool against Prdm5 (siPrdm5). Vinculin is used as a control for equal protein loading. Right panel. Quantification of calcified matrix deposition from calvarial osteoblasts treated with control or siPrdm5 after 14 days of stimulation with osteogenic media and stained with Alizarin red. Data are presented as the mean of 4 independent experiments ± standard deviation. Lower panel. Representative picture from the osteogenic differentiation experiment. (PDF) [file pgen.1002711.s002.pdf]

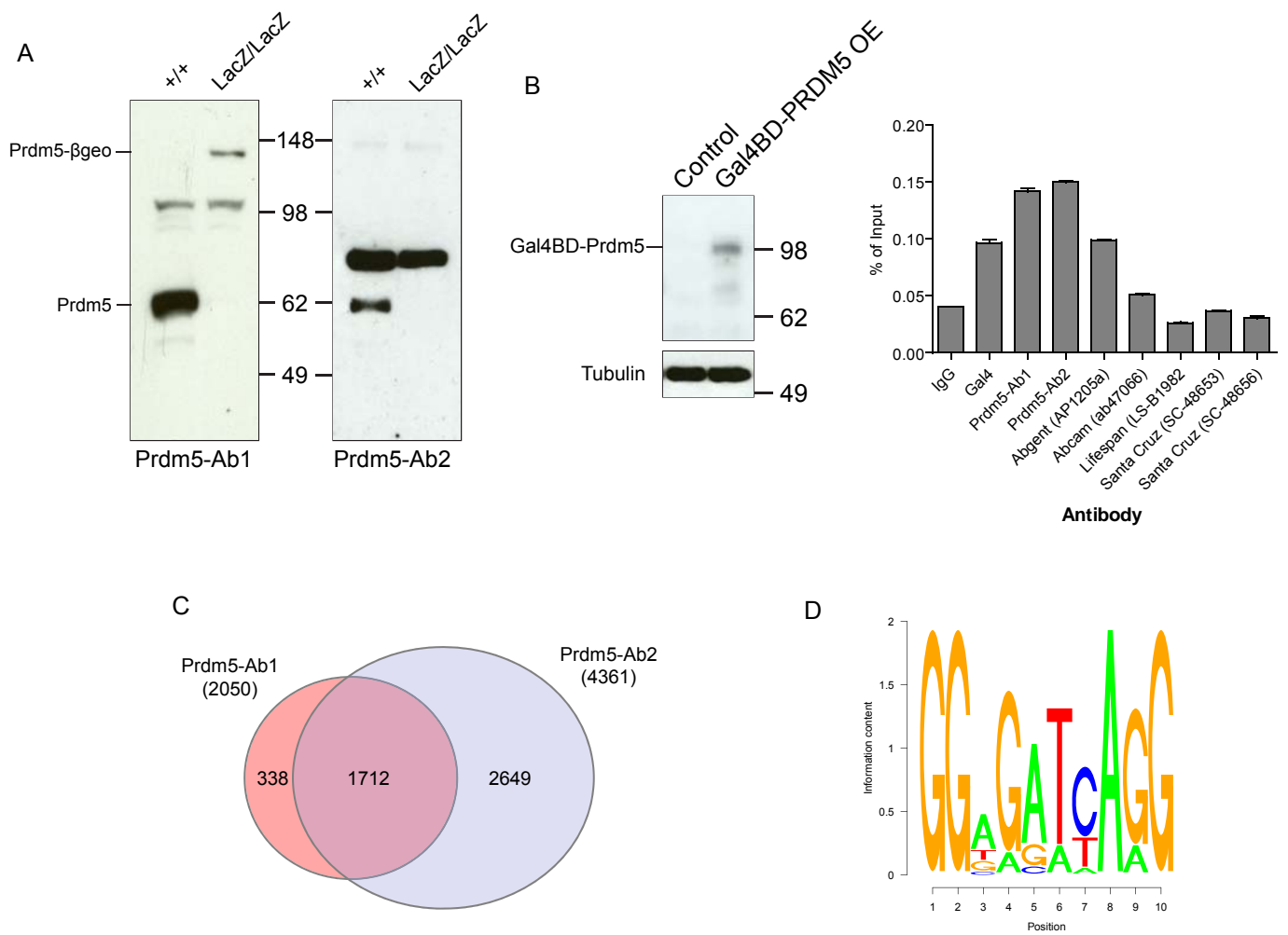

Supplement: Figure S3 — Validation of Prdm5 ChIP-grade antibodies. A) Western blot of +/+ and LacZ/LacZ mouse embryo fibroblasts using Prdm5 rabbit polyclonal antibodies Prdm5-ab1 and Prdm5-ab2. B) Left panel. Transfection control of HEK293 cells containing a stably integrated GAL4TkLuc reporter with empty vector or vector expressing Prdm5 fused to Gal4 DNA binding domain (Gal4BD-Prdm5 OE). Tubulin is used for equal protein loading. Right panel. Chromatin immunoprecipitation from Gal4BD-Prdm5-OE sample with IgG (negative control), Gal4 (positive control) and a range of in house-generated or commercially available Prdm5 antibodies. C) Venn diagram of the overlay of peaks identified by ChIP-seq in MC3T3 in experiment either with Prdm5-ab1 or Prdm5-ab2. D) Slogo representation of re-analysis of sequences retrieved by previously published random oligonucleotide experiment [4]. (PDF) [file pgen.1002711.s003.pdf]

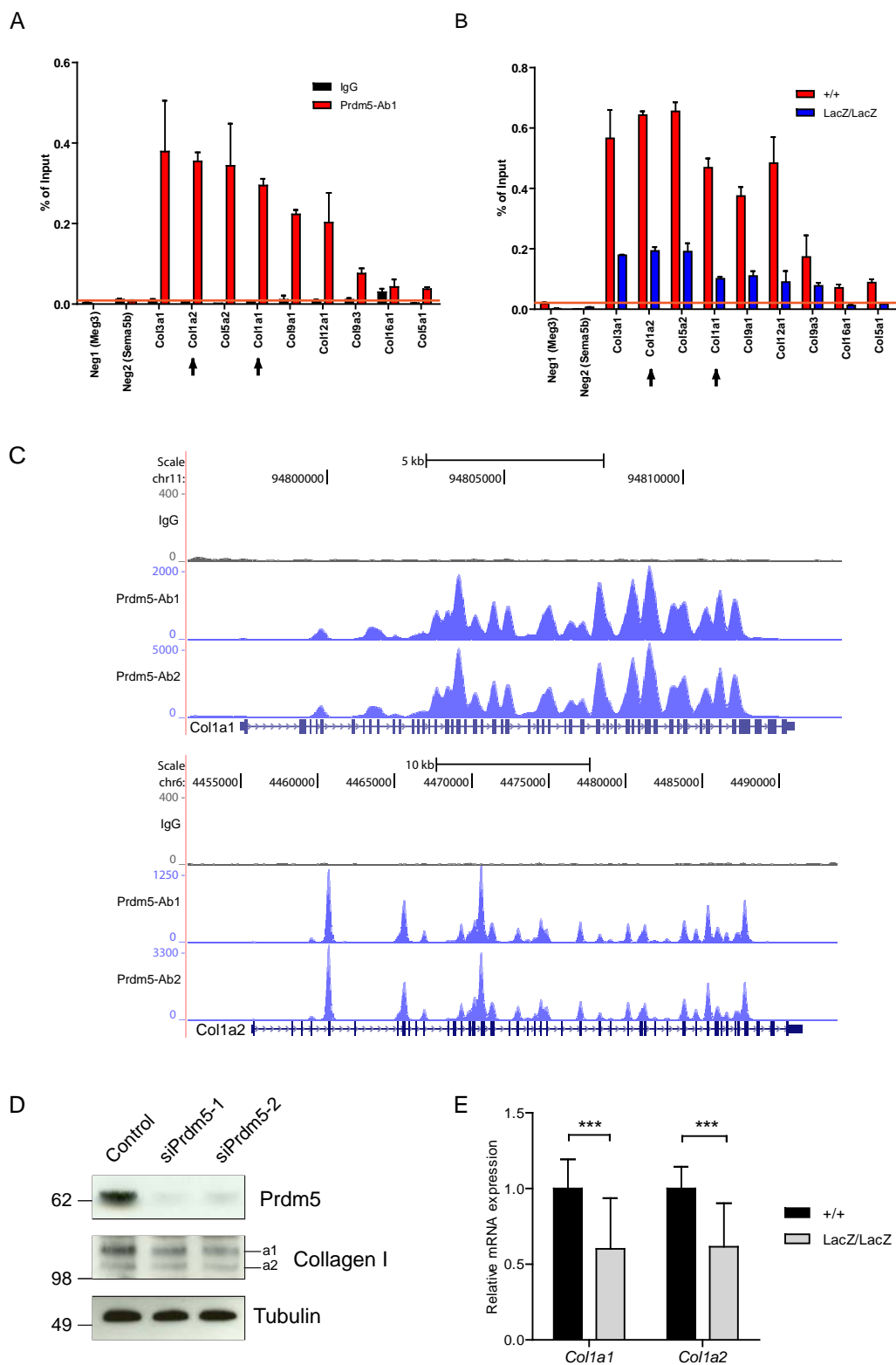

Supplement: Figure S4 — Prdm5 binds inside Col1a1 and Col1a2 gene bodies and regulates their transcription. A) ChIP-qPCR in primary calvarial osteoblasts with IgG or Prdm5-ab1 (black and red bars respectively) for Prdm5 binding site in selected collagen genes. Genomic regions around Meg3 and Sema5b are used as negative controls. Orange horizontal line represents the highest “noise” value obtained by ChIP-qPCR on a set of negative regions. B) ChIP-qPCR in wild type (red bars) and Prdm5LacZ/LacZ (blue bars) calvarial osteoblasts for a number of collagen genes. Genomic regions around Meg3 and Sema5b are used as negative controls. Orange horizontal line represents the highest “noise” value obtained by ChIP-qPCR on a set of negative regions. C) Genome browser snapshot for Col1a1 (upper panel) and Col1a2 (lower panel) regions. Tracks represent IgG, Prdm5-Ab1 and Prdm5-Ab2 sequencing results as indicated. D) Western blot for Prdm5 and Collagen I levels from MC3T3 cells treated for 72 hours with control or Prdm5 siRNA oligos. Tubulin is used for equal protein loading. E) qPCR analysis of WT and Prdm5LacZ/LacZ calvarial osteoblasts for Col1a1and Col1a2. Expression values were normalized to WT controls. *** = p<0.001 according to T-test, (+/+ n = 14, LacZ/LacZ n = 19 clones). (PDF) [file pgen.1002711.s004.pdf]

A

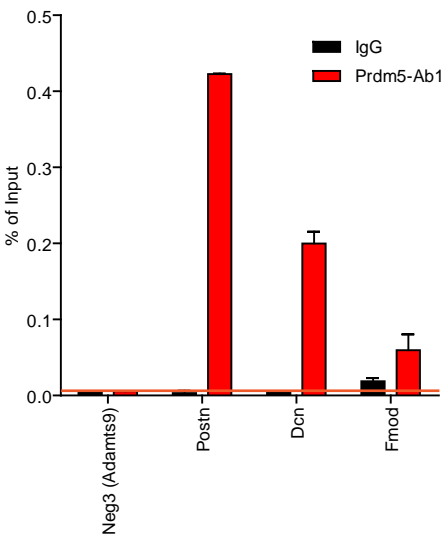

B

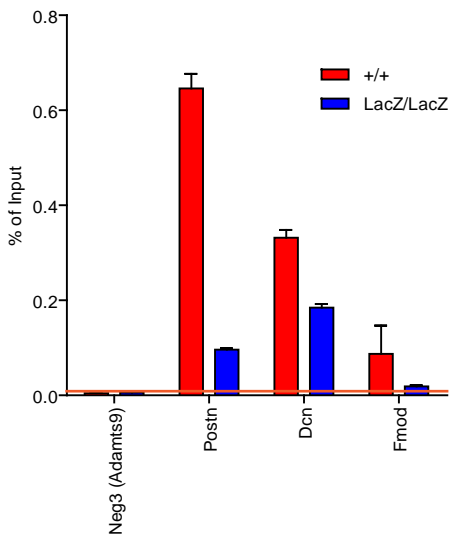

C

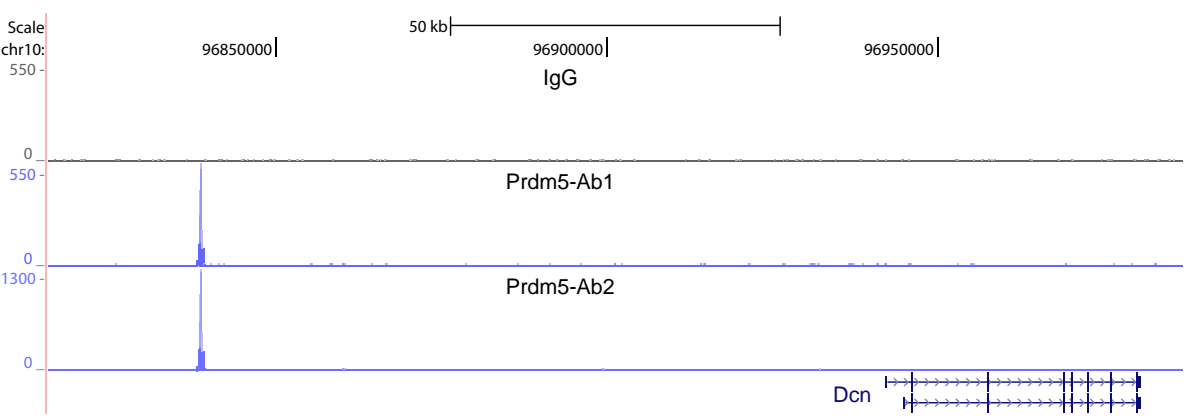

Supplement: Figure S5 — Prdm5 binds a distal element from Dcn gene TSS in calvarial osteoblasts. A) ChIP-qPCR in primary calvarial osteoblasts with IgG or Prdm5-ab1 (black and red bars respectively) for Prdm5 binding site assigned to ECM genes identified from ChIP-seq. Genomic region around AdamTS9 is used as negative control. Orange horizontal line represents the highest “noise” value obtained by ChIP-qPCR on a set of negative regions. B) ChIP-qPCR in wild type (red bars) and Prdm5LacZ/LacZ (blue bars) calvarial osteoblasts for the main peaks assigned to ECM genes identified from ChIP-seq. Genomic region around AdamTS9 is used as negative control. Orange horizontal line represents the highest “noise” value obtained by ChIP-qPCR on a set of negative regions. C) Genome browser snapshot for Prdm5 target region assigned to Dcn gene. Tracks represent IgG, Prdm5-Ab1 and Prdm5-Ab2 sequencing results as indicated. (PDF) [file pgen.1002711.s005.pdf]

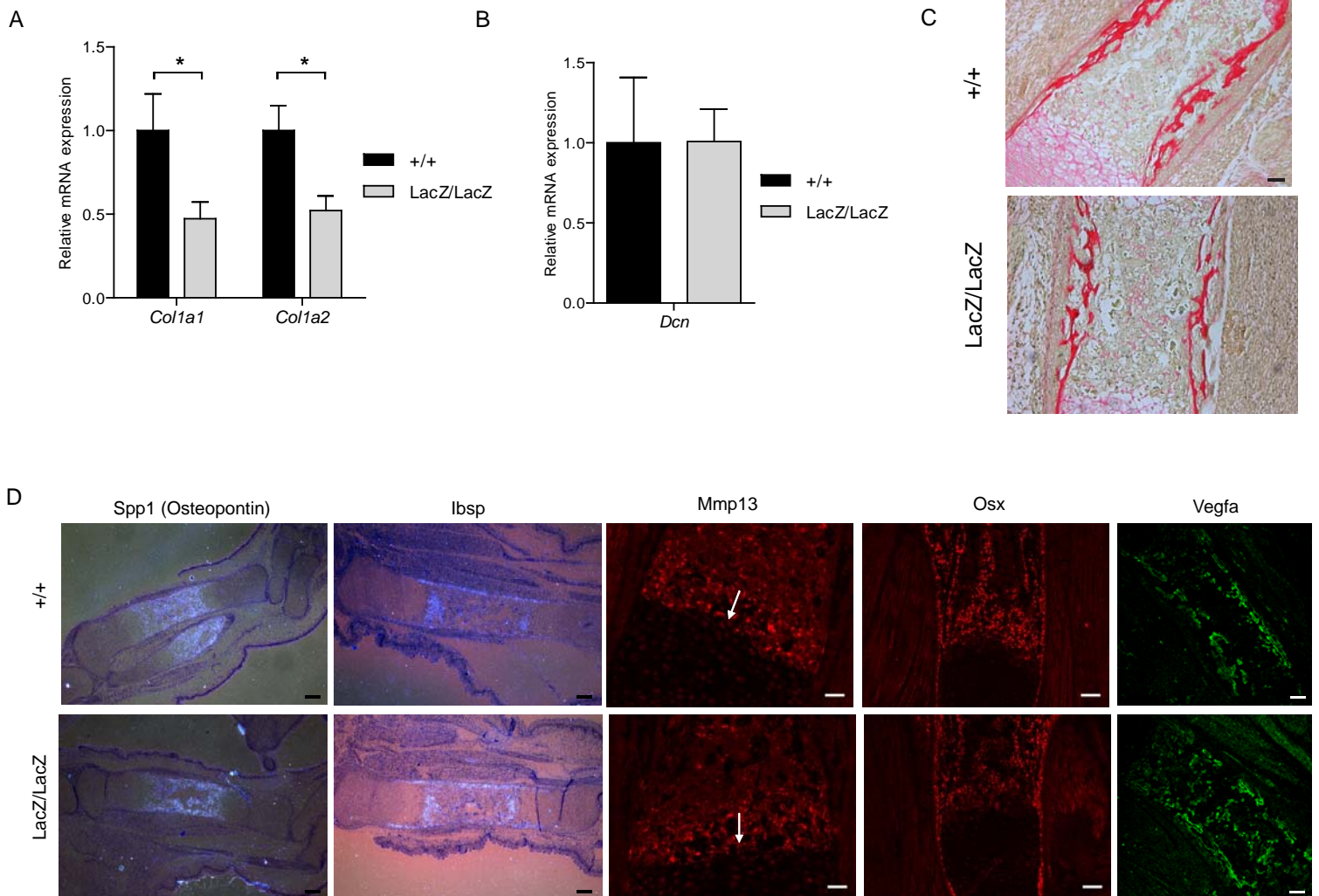

Supplement: Figure S6 — Characterization of Prdm5 target genes levels and osteoblasts markers in Prdm5LacZ/LacZ E16.5 limbs. A) qPCR analysis for Col1a1 and Col1a2 in E16.5 wild type and Prdm5LacZ/LacZ limbs (n = 4). * = p<0.05. B) qPCR analysis for Dcn in E16.5 WT and Prdm5LacZ/LacZ limbs (n = 4). C) Bright field image of picrosirius red staining in wild type and Prdm5LacZ/LacZ E16.5 tibia. D) In-situ hybridizations for Spp1/Osteopontin and Ibsp (bar = 400 um) and immunofluorescence micrographs of Mmp13 (bar = 100 µm), Vegfa and Osterix (bar = 200 µm) in WT and Prdm5LacZ/LacZ E16.5 embryo femurs or tibiae. White arrow indicates growth plate direction for orientation. All histological analyses are performed on parallel sections between littermate embryos of same sex. (PDF) [file pgen.1002711.s006.pdf]
