# Supplementary material for: Prdm5 Regulates Collagen Gene Transcription by Association with RNA Polymerase II in Developing Bone
Source: PLoS Genet. 2012 May 10;8(5):e1002711. doi: 10.1371/journal.pgen.1002711 (PMC3349747; doi:10.1371/journal.pgen.1002711)
Supplement: Table S1 — Genomic regions identified as high confidence Prdm5 targets from ChIP-seq experiment. Assigned ensembl gene IDs by annotation are indicated. (PDF) [file pgen.1002711.s007.pdf]

Galli_Table S1

Assigned_Ensembl_ID	Chromosome	start_position_(bp)	end_position_(bp)
ENSMUSG00000062588	chr1	4871837	4872254
ENSMUSG00000002459	chr1	5059967	5060278
ENSMUSG00000061024	chr1	9535068	9535495
ENSMUSG00000025932	chr1	14497004	14497547
ENSMUSG00000026158	chr1	23389277	23389827
ENSMUSG00000026147	chr1	24204058	24204570
ENSMUSG00000026147	chr1	24228155	24228950
ENSMUSG00000026147	chr1	24236796	24237333
ENSMUSG00000026147	chr1	24237646	24238796
ENSMUSG00000026147	chr1	24243848	24244404
ENSMUSG00000026147	chr1	24245953	24246389
ENSMUSG00000004768	chr1	33776277	33776942
ENSMUSG00000042215	chr1	33814124	33814767
ENSMUSG00000037470	chr1	36300828	36301242
ENSMUSG00000037408	chr1	36528590	36529028
ENSMUSG00000026113	chr1	37366832	37367441
ENSMUSG00000026110	chr1	37642521	37643007
ENSMUSG00000026083	chr1	38054735	38055178
ENSMUSG00000026082	chr1	38185841	38186368
ENSMUSG00000026082	chr1	38186864	38187155
ENSMUSG00000026082	chr1	38196242	38196671
ENSMUSG00000026074	chr1	39902860	39903449
ENSMUSG00000041763	chr1	43990511	43990948
ENSMUSG00000026048	chr1	44204686	44205229
ENSMUSG00000026043	chr1	45378425	45379172
ENSMUSG00000026043	chr1	45381918	45383126
ENSMUSG00000026043	chr1	45383338	45383694
ENSMUSG00000026043	chr1	45387156	45387639
ENSMUSG00000026043	chr1	45387707	45389309
ENSMUSG00000026043	chr1	45389671	45389967
ENSMUSG00000026043	chr1	45390167	45391773
ENSMUSG00000026043	chr1	45391838	45392187
ENSMUSG00000026043	chr1	45393298	45393787
ENSMUSG00000026043	chr1	45393948	45394490
ENSMUSG00000026043	chr1	45395060	45395840
ENSMUSG00000026043	chr1	45396506	45398375
ENSMUSG00000026043	chr1	45398664	45399493
ENSMUSG00000026043	chr1	45399960	45401137
ENSMUSG00000026043	chr1	45401198	45402076
ENSMUSG00000026043	chr1	45402536	45403047
ENSMUSG00000026042	chr1	45464278	45464894
ENSMUSG00000026042	chr1	45474262	45474677
ENSMUSG00000026042	chr1	45478576	45479603
ENSMUSG00000026042	chr1	45486462	45487030
ENSMUSG00000026042	chr1	45491409	45492066
ENSMUSG00000084531	chr1	50612043	50612359
ENSMUSG00000026107	chr1	51530247	51530575
ENSMUSG00000052331	chr1	54896280	54896869
ENSMUSG00000025980	chr1	55144552	55145126
ENSMUSG00000038331	chr1	57015688	57016196
ENSMUSG00000038331	chr1	57030039	57030511
ENSMUSG00000038331	chr1	57037885	57038491
ENSMUSG00000063558	chr1	58087061	58087393
ENSMUSG00000038242	chr1	58267004	58267638

ENSMUSG00000026036	chr1	58501911	58502396
ENSMUSG00000041075	chr1	59539725	59540142
ENSMUSG00000047361	chr1	59565323	59565820
ENSMUSG00000026018	chr1	60079626	60079915
ENSMUSG00000026019	chr1	60154692	60155564
ENSMUSG00000041003	chr1	60237101	60237446
ENSMUSG00000082160	chr1	60669822	60670281
ENSMUSG00000046526	chr1	62579235	62579532
ENSMUSG00000081617	chr1	62919990	62920649
ENSMUSG00000025959	chr1	64168518	64168874
ENSMUSG00000045005	chr1	64782836	64783223
ENSMUSG00000045005	chr1	64887471	64888062
ENSMUSG00000025945	chr1	65794067	65794561
ENSMUSG00000084332	chr1	69288442	69288852
ENSMUSG00000026176	chr1	74437858	74438336
ENSMUSG00000033021	chr1	75432283	75432941
ENSMUSG00000082528	chr1	75598077	75598683
ENSMUSG00000067158	chr1	82522376	82522793
ENSMUSG00000026150	chr1	82677162	82677497
ENSMUSG00000026238	chr1	88399527	88400067
ENSMUSG00000026240	chr1	88479216	88479555
ENSMUSG00000036500	chr1	89021738	89022118
ENSMUSG00000026255	chr1	89168980	89169542
ENSMUSG00000084195	chr1	90017099	90017613
ENSMUSG00000049866	chr1	90597710	90598135
ENSMUSG00000056128	chr1	92547723	92548168
ENSMUSG00000048126	chr1	92680674	92681043
ENSMUSG00000048126	chr1	92681445	92682082
ENSMUSG00000048126	chr1	92684583	92685085
ENSMUSG00000048126	chr1	92691347	92691843
ENSMUSG00000047443	chr1	93265741	93266206
ENSMUSG00000026313	chr1	93977506	93977967
ENSMUSG00000034220	chr1	94711289	94711891
ENSMUSG00000026274	chr1	95239392	95240113
ENSMUSG00000026277	chr1	95532394	95532999
ENSMUSG00000077413	chr1	96577611	96577969
ENSMUSG00000038866	chr1	107783654	107783982
ENSMUSG00000067001	chr1	109315402	109315772
ENSMUSG00000048402	chr1	120928436	120928936
ENSMUSG00000026390	chr1	122382156	122382718
ENSMUSG00000001674	chr1	123464210	123464758
ENSMUSG00000070689	chr1	128652189	128652719
ENSMUSG00000036086	chr1	129999121	129999618
ENSMUSG00000080357	chr1	130445562	130445990
ENSMUSG00000026427	chr1	133052148	133052555
ENSMUSG00000026437	chr1	134036094	134036642
ENSMUSG00000079330	chr1	134071514	134072119
ENSMUSG00000053024	chr1	134435253	134435693
ENSMUSG00000041577	chr1	135862090	135862589
ENSMUSG00000041559	chr1	135935901	135936607
ENSMUSG00000041559	chr1	135938382	135939189
ENSMUSG00000041559	chr1	135939872	135940460
ENSMUSG00000042305	chr1	136258496	136258895
ENSMUSG00000026456	chr1	136302373	136302900
ENSMUSG00000042207	chr1	136453363	136453689
ENSMUSG00000077846	chr1	136642042	136642640
ENSMUSG00000026424	chr1	137092432	137093003
ENSMUSG00000048096	chr1	137220802	137221635

ENSMUSG00000009418	chr1	137568652	137569432
ENSMUSG00000009418	chr1	137583835	137584205
ENSMUSG00000073552	chr1	138645540	138646211
ENSMUSG00000026398	chr1	138976222	138976774
ENSMUSG00000006005	chr1	152239600	152240112
ENSMUSG00000043019	chr1	153552908	153553648
ENSMUSG00000032666	chr1	153846561	153847165
ENSMUSG00000026473	chr1	155746990	155747320
ENSMUSG00000050565	chr1	157854909	157855184
ENSMUSG00000026592	chr1	159077618	159078089
ENSMUSG00000033488	chr1	159366263	159366823
ENSMUSG00000073530	chr1	160847139	160847783
ENSMUSG00000047661	chr1	160959740	160960198
ENSMUSG00000040782	chr1	161163016	161163436
ENSMUSG00000053332	chr1	162965189	162965703
ENSMUSG00000026586	chr1	165223915	165224427
ENSMUSG00000026586	chr1	165242190	165242986
ENSMUSG00000040124	chr1	165333611	165333996
ENSMUSG00000026585	chr1	165709295	165709708
ENSMUSG00000026566	chr1	167572854	167573325
ENSMUSG00000026560	chr1	168649366	168649907
ENSMUSG00000053664	chr1	169176968	169177466
ENSMUSG00000038463	chr1	172575891	172576594
ENSMUSG00000038463	chr1	172594879	172595727
ENSMUSG00000006403	chr1	173179800	173180178
ENSMUSG00000052423	chr1	173192829	173193435
ENSMUSG00000003464	chr1	174064184	174064575
ENSMUSG00000026500	chr1	180249040	180249698
ENSMUSG00000055067	chr1	181406642	181407024
ENSMUSG00000055067	chr1	181408040	181408582
ENSMUSG00000026491	chr1	181792993	181793523
ENSMUSG00000026489	chr1	182098095	182098562
ENSMUSG00000066652	chr1	182824792	182825149
ENSMUSG00000038776	chr1	182945796	182946447
ENSMUSG00000026509	chr1	184430912	184431496
ENSMUSG00000079164	chr1	184886693	184887114
ENSMUSG00000039384	chr1	185860051	185860665
ENSMUSG00000001305	chr1	188573050	188573424
ENSMUSG00000039210	chr1	189102805	189103087
ENSMUSG00000026604	chr1	191641010	191641432
ENSMUSG00000084434	chr1	192349481	192349857
ENSMUSG00000037568	chr1	192802147	192802938
ENSMUSG00000062510	chr1	192887676	192888115
ENSMUSG00000026627	chr1	193149591	193149894
ENSMUSG00000026622	chr1	193650443	193651007
ENSMUSG00000037375	chr1	194607439	194607858
ENSMUSG00000037375	chr1	194615241	194615670
ENSMUSG00000065533	chr1	195395432	195396008
ENSMUSG00000016481	chr1	196957031	196957644
ENSMUSG00000065792	chr10	6267091	6267469
ENSMUSG00000019796	chr10	7320203	7320656
ENSMUSG00000015755	chr10	7636609	7637186
ENSMUSG00000015755	chr10	7710260	7710491
ENSMUSG00000019832	chr10	10277701	10278242
ENSMUSG00000064531	chr10	13635179	13635686
ENSMUSG00000019850	chr10	18721763	18722290
ENSMUSG00000071369	chr10	19696831	19697440
ENSMUSG00000053219	chr10	21928563	21929182

ENSMUSG00000075295	chr10	22118695	22119381
ENSMUSG00000010461	chr10	23216119	23216513
ENSMUSG00000061983	chr10	23507068	23507470
ENSMUSG00000081259	chr10	24288090	24288575
ENSMUSG00000019791	chr10	30338122	30338596
ENSMUSG00000039462	chr10	34113822	34115536
ENSMUSG00000077576	chr10	37805280	37805663
ENSMUSG00000019846	chr10	38606322	38606813
ENSMUSG00000019843	chr10	39011537	39011879
ENSMUSG00000045555	chr10	40402849	40403474
ENSMUSG00000075216	chr10	41923042	41923423
ENSMUSG00000019802	chr10	42481907	42482399
ENSMUSG00000038151	chr10	44249020	44249524
ENSMUSG00000019857	chr10	53315586	53315988
ENSMUSG00000038122	chr10	55856562	55857018
ENSMUSG00000003226	chr10	57909328	57909786
ENSMUSG00000020109	chr10	59350366	59350729
ENSMUSG00000020108	chr10	59413372	59413812
ENSMUSG00000077843	chr10	59692962	59693434
ENSMUSG00000020101	chr10	59817380	59817995
ENSMUSG00000020100	chr10	60224949	60225321
ENSMUSG00000020097	chr10	60609899	60610352
ENSMUSG00000020092	chr10	60837593	60838127
ENSMUSG00000020086	chr10	61325578	61326025
ENSMUSG00000058806	chr10	61356522	61356814
ENSMUSG00000058806	chr10	61435538	61435893
ENSMUSG00000042846	chr10	63552695	63553265
ENSMUSG00000075004	chr10	66398527	66398945
ENSMUSG00000075004	chr10	66465064	66465543
ENSMUSG00000037846	chr10	67446546	67447055
ENSMUSG00000069601	chr10	68998140	68998630
ENSMUSG00000033444	chr10	74675036	74675424
ENSMUSG00000046807	chr10	75013742	75014252
ENSMUSG00000033208	chr10	75720535	75721044
ENSMUSG00000020241	chr10	76070284	76071143
ENSMUSG00000020241	chr10	76071757	76072371
ENSMUSG00000020241	chr10	76073071	76073643
ENSMUSG00000020241	chr10	76096580	76097176
ENSMUSG00000020241	chr10	76111627	76112019
ENSMUSG00000020241	chr10	76127789	76128224
ENSMUSG00000001119	chr10	76177674	76178318
ENSMUSG00000001119	chr10	76179137	76180948
ENSMUSG00000001119	chr10	76183617	76183958
ENSMUSG00000020262	chr10	76881021	76881589
ENSMUSG00000032788	chr10	77921316	77921838
ENSMUSG00000020317	chr10	79047233	79047819
ENSMUSG00000020308	chr10	79132189	79132786
ENSMUSG00000003068	chr10	79580244	79580654
ENSMUSG00000045193	chr10	79621285	79621897
ENSMUSG00000045193	chr10	79626403	79626936
ENSMUSG00000045193	chr10	79627666	79628066
ENSMUSG00000020135	chr10	79757459	79757948
ENSMUSG00000020190	chr10	80139220	80139725
ENSMUSG00000020219	chr10	80362961	80363671
ENSMUSG00000035011	chr10	80598457	80599034
ENSMUSG00000004934	chr10	80624641	80625211
ENSMUSG00000079936	chr10	80874257	80874850
ENSMUSG00000034771	chr10	81035163	81035852

ENSMUSG00000074860	chr10	81106414	81106797
ENSMUSG00000078437	chr10	81233581	81234170
ENSMUSG00000034674	chr10	82093225	82093778
ENSMUSG00000020263	chr10	83071362	83071832
ENSMUSG00000020048	chr10	86168165	86169101
ENSMUSG00000035459	chr10	86546927	86547405
ENSMUSG00000078109	chr10	89335184	89335575
ENSMUSG00000019961	chr10	90631332	90631683
ENSMUSG00000019961	chr10	90633256	90633695
ENSMUSG00000019961	chr10	90703949	90704425
ENSMUSG00000008398	chr10	92824219	92824911
ENSMUSG00000020019	chr10	93129810	93130169
ENSMUSG00000020023	chr10	93956875	93957496
ENSMUSG00000074785	chr10	94415149	94415377
ENSMUSG00000019929	chr10	96838326	96839043
ENSMUSG00000047025	chr10	97132799	97133340
ENSMUSG00000058799	chr10	110910709	110911027
ENSMUSG00000020137	chr10	114821427	114822054
ENSMUSG00000052681	chr10	117284374	117284813
ENSMUSG00000020114	chr10	118677273	118677714
ENSMUSG00000056758	chr10	120019484	120019889
ENSMUSG00000056758	chr10	120047179	120047586
ENSMUSG00000020105	chr10	125402846	125403394
ENSMUSG00000019467	chr10	126626059	126626534
ENSMUSG00000025417	chr10	126665987	126666439
ENSMUSG00000025409	chr10	126721851	126722327
ENSMUSG00000040249	chr10	127052421	127052925
ENSMUSG00000040043	chr10	127588045	127588347
ENSMUSG00000005683	chr10	127774816	127775159
ENSMUSG00000039810	chr10	127984646	127985265
ENSMUSG00000018166	chr10	128021907	128022336
ENSMUSG00000047090	chr10	128240853	128241456
ENSMUSG00000045559	chr10	129665366	129665644
ENSMUSG00000048807	chr11	3812847	3813486
ENSMUSG00000034412	chr11	4097645	4098316
ENSMUSG00000034164	chr11	5028676	5029313
ENSMUSG00000020393	chr11	5190149	5190710
ENSMUSG00000083108	chr11	5550562	5551022
ENSMUSG00000020473	chr11	5762747	5763296
ENSMUSG00000057897	chr11	5970839	5971475
ENSMUSG00000057897	chr11	5971612	5972156
ENSMUSG00000083553	chr11	8415332	8415643
ENSMUSG00000020422	chr11	8429849	8430346
ENSMUSG00000020422	chr11	8439647	8440688
ENSMUSG00000035455	chr11	11726920	11727358
ENSMUSG00000020173	chr11	12364121	12364619
ENSMUSG00000020122	chr11	16666371	16666879
ENSMUSG00000044966	chr11	16835612	16836074
ENSMUSG00000000581	chr11	17157361	17157888
ENSMUSG00000044066	chr11	20129252	20129733
ENSMUSG00000084252	chr11	20572331	20572981
ENSMUSG00000051650	chr11	22759739	22760262
ENSMUSG00000007739	chr11	22890337	22890755
ENSMUSG00000032673	chr11	29414508	29414940
ENSMUSG00000020287	chr11	32124516	32125017
ENSMUSG00000020272	chr11	32484998	32485466
ENSMUSG00000081254	chr11	32807358	32807811
ENSMUSG00000082587	chr11	37801903	37802138

ENSMUSG00000083806	chr11	39760820	39761293
ENSMUSG00000044847	chr11	45757757	45758164
ENSMUSG00000020411	chr11	45977226	45977674
ENSMUSG00000036644	chr11	49959516	49960100
ENSMUSG00000007850	chr11	50190428	50191009
ENSMUSG00000036545	chr11	50415728	50416584
ENSMUSG00000036545	chr11	50440791	50441159
ENSMUSG00000020359	chr11	51382775	51383232
ENSMUSG00000020359	chr11	51384410	51384890
ENSMUSG00000007777	chr11	51515576	51515855
ENSMUSG00000020387	chr11	51706692	51707143
ENSMUSG00000018899	chr11	53575239	53575764
ENSMUSG00000018899	chr11	53583523	53583945
ENSMUSG00000035992	chr11	54253287	54253834
ENSMUSG00000018340	chr11	54853085	54853647
ENSMUSG00000082257	chr11	55203027	55204471
ENSMUSG00000018593	chr11	55241547	55242054
ENSMUSG00000020519	chr11	57614205	57614979
ENSMUSG00000020515	chr11	57917980	57918374
ENSMUSG00000037243	chr11	58115221	58115693
ENSMUSG00000049755	chr11	58136685	58137371
ENSMUSG00000020496	chr11	58752108	58752700
ENSMUSG00000009900	chr11	59107059	59107591
ENSMUSG00000082148	chr11	60115336	60115779
ENSMUSG00000020536	chr11	60512718	60513421
ENSMUSG00000002814	chr11	60590584	60591081
ENSMUSG00000043284	chr11	60692219	60692849
ENSMUSG00000042506	chr11	60988940	60989226
ENSMUSG00000001036	chr11	61413713	61414192
ENSMUSG00000083212	chr11	61833992	61834785
ENSMUSG00000042200	chr11	62780337	62781111
ENSMUSG00000020542	chr11	65200031	65200507
ENSMUSG00000077908	chr11	68178055	68178578
ENSMUSG00000020897	chr11	68858971	68859458
ENSMUSG00000059278	chr11	69209131	69209935
ENSMUSG00000018476	chr11	69228704	69229039
ENSMUSG00000005237	chr11	69361707	69362244
ENSMUSG00000018752	chr11	69508948	69509302
ENSMUSG00000047284	chr11	69716003	69716380
ENSMUSG00000018293	chr11	70466696	70467078
ENSMUSG00000018446	chr11	70796218	70797099
ENSMUSG00000077456	chr11	71683350	71684037
ENSMUSG00000020794	chr11	72419928	72420357
ENSMUSG00000038351	chr11	74710741	74711128
ENSMUSG00000020850	chr11	75300290	75300780
ENSMUSG00000020847	chr11	75779852	75780228
ENSMUSG00000080763	chr11	76285966	76286426
ENSMUSG00000037857	chr11	77499247	77499591
ENSMUSG00000017453	chr11	77711917	77712413
ENSMUSG00000002058	chr11	78157394	78157732
ENSMUSG00000017686	chr11	80022645	80023199
ENSMUSG00000035441	chr11	80470151	80470760
ENSMUSG00000020702	chr11	82008438	82008874
ENSMUSG00000020696	chr11	82670399	82670918
ENSMUSG00000018068	chr11	86070735	86071306
ENSMUSG00000047126	chr11	86570923	86571301
ENSMUSG00000023723	chr11	87977704	87978791
ENSMUSG00000023723	chr11	87979085	87979444

ENSMUSG00000023723	chr11	87993783	87994139
ENSMUSG00000023723	chr11	87994176	87994572
ENSMUSG00000034031	chr11	88105291	88105595
ENSMUSG00000078718	chr11	90216290	90217281
ENSMUSG00000037573	chr11	94022625	94023112
ENSMUSG00000020864	chr11	94189391	94189936
ENSMUSG00000064512	chr11	94776955	94777507
ENSMUSG00000001506	chr11	94798834	94812998
ENSMUSG00000038994	chr11	94813180	94813960
ENSMUSG00000038994	chr11	94814632	94815147
ENSMUSG00000038994	chr11	94815592	94817189
ENSMUSG00000038994	chr11	94818806	94819245
ENSMUSG00000038976	chr11	94855156	94855795
ENSMUSG00000020871	chr11	95007357	95007780
ENSMUSG00000000120	chr11	95434314	95434699
ENSMUSG00000075595	chr11	95574680	95575111
ENSMUSG00000050860	chr11	95669603	95670099
ENSMUSG00000018381	chr11	95718606	95719123
ENSMUSG00000018381	chr11	95720301	95720763
ENSMUSG00000047653	chr11	95824491	95824951
ENSMUSG00000013415	chr11	95868559	95869006
ENSMUSG00000056648	chr11	96138911	96139306
ENSMUSG00000018678	chr11	96854378	96854948
ENSMUSG00000049807	chr11	97301831	97302278
ENSMUSG00000018537	chr11	97556959	97557448
ENSMUSG00000038366	chr11	97662103	97662564
ENSMUSG00000017195	chr11	98412139	98412563
ENSMUSG00000017221	chr11	98544127	98544757
ENSMUSG00000037992	chr11	98793958	98794334
ENSMUSG00000037992	chr11	98797868	98798374
ENSMUSG00000064491	chr11	98802138	98802482
ENSMUSG00000035592	chr11	99873033	99873637
ENSMUSG00000057723	chr11	99885975	99886480
ENSMUSG00000043485	chr11	99899352	99899936
ENSMUSG00000048981	chr11	99908697	99909319
ENSMUSG00000048013	chr11	99954044	99954600
ENSMUSG00000053654	chr11	100151659	100152002
ENSMUSG00000006931	chr11	100275517	100276243
ENSMUSG00000019173	chr11	100599435	100599997
ENSMUSG00000004044	chr11	100830183	100830523
ENSMUSG00000004044	chr11	100831209	100831670
ENSMUSG00000001751	chr11	100930908	100931630
ENSMUSG00000035198	chr11	100981908	100982470
ENSMUSG00000059995	chr11	102158370	102159037
ENSMUSG00000034664	chr11	102328496	102328933
ENSMUSG00000050288	chr11	102467309	102467676
ENSMUSG00000020936	chr11	102919883	102920427
ENSMUSG00000048878	chr11	102976586	102977021
ENSMUSG00000020681	chr11	105835769	105836326
ENSMUSG00000040481	chr11	106992620	106992878
ENSMUSG00000077836	chr11	113061892	113062239
ENSMUSG00000034706	chr11	114586127	114586808
ENSMUSG00000034586	chr11	115219699	115220271
ENSMUSG00000084277	chr11	115527994	115528425
ENSMUSG00000020773	chr11	115971238	115971686
ENSMUSG00000020806	chr11	116482470	116482931
ENSMUSG00000075410	chr11	116513531	116514026
ENSMUSG00000020810	chr11	116518938	116519661

ENSMUSG00000059248	chr11	117094292	117095241
ENSMUSG00000059248	chr11	117117237	117117912
ENSMUSG00000054418	chr11	117418535	117419063
ENSMUSG00000082562	chr11	117773939	117774396
ENSMUSG00000053113	chr11	117829324	117830087
ENSMUSG00000033909	chr11	118151154	118151747
ENSMUSG00000076433	chr11	118207269	118207691
ENSMUSG00000017466	chr11	118214665	118215153
ENSMUSG00000025577	chr11	118884412	118884868
ENSMUSG00000025578	chr11	118914527	118914878
ENSMUSG00000025372	chr11	119810111	119810564
ENSMUSG00000025379	chr11	120012409	120012760
ENSMUSG00000056649	chr11	120047259	120047796
ENSMUSG00000075389	chr11	120051280	120051788
ENSMUSG00000039741	chr11	120112151	120112743
ENSMUSG00000039741	chr11	120113730	120114315
ENSMUSG00000062825	chr11	120207900	120209267
ENSMUSG00000062825	chr11	120209310	120209853
ENSMUSG00000039450	chr11	120588121	120588609
ENSMUSG00000025153	chr11	120685676	120686086
ENSMUSG00000025173	chr11	121215498	121215908
ENSMUSG00000025173	chr11	121216175	121216640
ENSMUSG00000020635	chr12	4848260	4848851
ENSMUSG00000054459	chr12	11392222	11392542
ENSMUSG00000073228	chr12	13280784	13281106
ENSMUSG00000020580	chr12	16877752	16878238
ENSMUSG00000061458	chr12	17345197	17345666
ENSMUSG00000020649	chr12	25393126	25393499
ENSMUSG00000020674	chr12	30596538	30597020
ENSMUSG00000002900	chr12	31954430	31954864
ENSMUSG00000020659	chr12	32184282	32185026
ENSMUSG00000056899	chr12	41514879	41515334
ENSMUSG00000054302	chr12	55796272	55796767
ENSMUSG00000079104	chr12	58331383	58331890
ENSMUSG00000020993	chr12	60162163	60162626
ENSMUSG00000048285	chr12	71899374	71899845
ENSMUSG00000056359	chr12	74563014	74563362
ENSMUSG00000052609	chr12	77634024	77634574
ENSMUSG00000021114	chr12	79962168	79962790
ENSMUSG00000021127	chr12	81212616	81213027
ENSMUSG00000015143	chr12	81361234	81361610
ENSMUSG00000065115	chr12	81978997	81979346
ENSMUSG00000021136	chr12	82200180	82200601
ENSMUSG00000042700	chr12	83348784	83349101
ENSMUSG00000021245	chr12	86603455	86603795
ENSMUSG00000034168	chr12	88223732	88224300
ENSMUSG00000034157	chr12	88287921	88288324
ENSMUSG00000033713	chr12	100770388	100770879
ENSMUSG00000021070	chr12	106737357	106737912
ENSMUSG00000072840	chr12	109492899	109493187
ENSMUSG00000021265	chr12	110070454	110070894
ENSMUSG00000078977	chr12	111619143	111619596
ENSMUSG00000018707	chr12	111840975	111841463
ENSMUSG00000037896	chr12	112278202	112278476
ENSMUSG00000021277	chr12	112406052	112406401
ENSMUSG00000037679	chr12	113831357	113831770
ENSMUSG00000021144	chr12	114338713	114339337
ENSMUSG00000006356	chr12	114376781	114377242

ENSMUSG00000042050	chr12	117501009	117501523
ENSMUSG00000021171	chr12	117518950	117519449
ENSMUSG00000021147	chr13	8869809	8870358
ENSMUSG00000057069	chr13	12669214	12669686
ENSMUSG00000070172	chr13	16852838	16853136
ENSMUSG00000022228	chr13	21545161	21545918
ENSMUSG00000060832	chr13	21826900	21827449
ENSMUSG00000052565	chr13	23646975	23647544
ENSMUSG00000036181	chr13	23830778	23831294
ENSMUSG00000021337	chr13	24114254	24114767
ENSMUSG00000021357	chr13	31065318	31066153
ENSMUSG00000021400	chr13	32893415	32893869
ENSMUSG00000021411	chr13	34720602	34721071
ENSMUSG00000039087	chr13	37808159	37808555
ENSMUSG00000038982	chr13	38694870	38695362
ENSMUSG00000021359	chr13	40825050	40825421
ENSMUSG00000021364	chr13	41344687	41345301
ENSMUSG00000046876	chr13	45878617	45879113
ENSMUSG00000021379	chr13	48357289	48357853
ENSMUSG00000038025	chr13	48967509	48967957
ENSMUSG00000037960	chr13	49309007	49309569
ENSMUSG00000021448	chr13	51692476	51692782
ENSMUSG00000021464	chr13	53252561	53252954
ENSMUSG00000021464	chr13	53411740	53412336
ENSMUSG00000034928	chr13	54795624	54796089
ENSMUSG00000005320	chr13	55269105	55269605
ENSMUSG00000021488	chr13	55290881	55291335
ENSMUSG00000021485	chr13	55430565	55431033
ENSMUSG00000034675	chr13	55584628	55585268
ENSMUSG00000021493	chr13	55612159	55612831
ENSMUSG00000035367	chr13	58501748	58502363
ENSMUSG00000065599	chr13	63343000	63343337
ENSMUSG00000065599	chr13	63362203	63362712
ENSMUSG00000065599	chr13	63389380	63389835
ENSMUSG00000021466	chr13	63753442	63753880
ENSMUSG00000001504	chr13	72764989	72765456
ENSMUSG00000021608	chr13	73558195	73558648
ENSMUSG00000021608	chr13	73595806	73596303
ENSMUSG00000021579	chr13	74501082	74501527
ENSMUSG00000021591	chr13	75970987	75971362
ENSMUSG00000021619	chr13	91312822	91313311
ENSMUSG00000021619	chr13	91313319	91314090
ENSMUSG00000021619	chr13	91314554	91315073
ENSMUSG00000021619	chr13	91363574	91363838
ENSMUSG00000014850	chr13	93127423	93127767
ENSMUSG00000021684	chr13	95994371	95994820
ENSMUSG00000048376	chr13	96426558	96426987
ENSMUSG00000021670	chr13	97434883	97435334
ENSMUSG00000021662	chr13	98976396	98976727
ENSMUSG00000021709	chr13	104709227	104709589
ENSMUSG00000049233	chr13	108274330	108274765
ENSMUSG00000032846	chr13	108615109	108615677
ENSMUSG00000021699	chr13	109316757	109317247
ENSMUSG00000067122	chr13	112368142	112368658
ENSMUSG00000021754	chr13	112583109	112583476
ENSMUSG00000021754	chr13	112599325	112599644
ENSMUSG00000016018	chr13	113717439	113717853
ENSMUSG00000057445	chr14	6011829	6012202

ENSMUSG00000079370	chr14	6354730	6355134
ENSMUSG00000021785	chr14	17081605	17082128
ENSMUSG00000021809	chr14	21113530	21114137
ENSMUSG00000039357	chr14	21514401	21514723
ENSMUSG00000021819	chr14	21526233	21526684
ENSMUSG00000021820	chr14	21611011	21611441
ENSMUSG00000021767	chr14	22343149	22344094
ENSMUSG00000007817	chr14	26277479	26277974
ENSMUSG00000057293	chr14	26554480	26555061
ENSMUSG00000057293	chr14	26569090	26569595
ENSMUSG00000072678	chr14	26758336	26758893
ENSMUSG00000072676	chr14	26898211	26898661
ENSMUSG00000040760	chr14	27782959	27783314
ENSMUSG00000021962	chr14	31292796	31293159
ENSMUSG00000006522	chr14	31744110	31744666
ENSMUSG00000021892	chr14	32249110	32249626
ENSMUSG00000041408	chr14	35486660	35487048
ENSMUSG00000037833	chr14	41730959	41731654
ENSMUSG00000021795	chr14	41989653	41989909
ENSMUSG00000036339	chr14	49066264	49066761
ENSMUSG00000049295	chr14	52638890	52639584
ENSMUSG00000053754	chr14	52876881	52877633
ENSMUSG00000021982	chr14	60216533	60217138
ENSMUSG00000021928	chr14	61979000	61979399
ENSMUSG00000021929	chr14	62058569	62059021
ENSMUSG00000065459	chr14	62300899	62301225
ENSMUSG00000060012	chr14	65270931	65271351
ENSMUSG00000055404	chr14	66539602	66540202
ENSMUSG00000022040	chr14	66730557	66731099
ENSMUSG00000022043	chr14	66915863	66916531
ENSMUSG00000022052	chr14	67739100	67739698
ENSMUSG00000022091	chr14	70604806	70605288
ENSMUSG00000047911	chr14	71096215	71096508
ENSMUSG00000022105	chr14	73725921	73726362
ENSMUSG00000021996	chr14	75132101	75132636
ENSMUSG00000065799	chr14	76384803	76385479
ENSMUSG00000022010	chr14	76815964	76816450
ENSMUSG00000052584	chr14	77161705	77162065
ENSMUSG00000034795	chr14	77483412	77484017
ENSMUSG00000022025	chr14	80060763	80061516
ENSMUSG00000033166	chr14	99543240	99543822
ENSMUSG00000063410	chr14	121777453	121777682
ENSMUSG00000022148	chr15	6514983	6515537
ENSMUSG00000022148	chr15	6529892	6530356
ENSMUSG00000048424	chr15	8824827	8825337
ENSMUSG00000022253	chr15	8968544	8968986
ENSMUSG00000022246	chr15	10609388	10609999
ENSMUSG00000058914	chr15	10887609	10888042
ENSMUSG00000047497	chr15	11047856	11048315
ENSMUSG00000047497	chr15	11132749	11133250
ENSMUSG00000022200	chr15	12251364	12252074
ENSMUSG00000022261	chr15	32708374	32708866
ENSMUSG00000039007	chr15	33151398	33151964
ENSMUSG00000058600	chr15	34372586	34372992
ENSMUSG00000022283	chr15	36539163	36539426
ENSMUSG00000022312	chr15	51686574	51686813
ENSMUSG00000022371	chr15	55141908	55142468
ENSMUSG00000049155	chr15	55303083	55303438

ENSMUSG00000049155	chr15	55328762	55329365
ENSMUSG00000051225	chr15	57807810	57808401
ENSMUSG00000022360	chr15	57966413	57966865
ENSMUSG00000032501	chr15	59374847	59375329
ENSMUSG00000072566	chr15	61875316	61875667
ENSMUSG00000072566	chr15	61956632	61956955
ENSMUSG00000056590	chr15	62400520	62401173
ENSMUSG00000053469	chr15	66507128	66507550
ENSMUSG00000005124	chr15	66723402	66725267
ENSMUSG00000005124	chr15	66731009	66731540
ENSMUSG00000013846	chr15	67006775	67007340
ENSMUSG00000045567	chr15	71638214	71638661
ENSMUSG00000045567	chr15	71678690	71679168
ENSMUSG00000079022	chr15	71720745	71721241
ENSMUSG00000079022	chr15	71728107	71728637
ENSMUSG00000036661	chr15	73366085	73366552
ENSMUSG00000084493	chr15	73555533	73555971
ENSMUSG00000034730	chr15	74326141	74326562
ENSMUSG00000044276	chr15	74539366	74540255
ENSMUSG00000022579	chr15	75412162	75412797
ENSMUSG00000022574	chr15	75724442	75724787
ENSMUSG00000022570	chr15	75760164	75760522
ENSMUSG00000022568	chr15	75899332	75899859
ENSMUSG00000022568	chr15	75900002	75900510
ENSMUSG00000022565	chr15	76018985	76019402
ENSMUSG00000022565	chr15	76036397	76036813
ENSMUSG00000033565	chr15	77160139	77160587
ENSMUSG00000049521	chr15	78673650	78674082
ENSMUSG00000049521	chr15	78678942	78679436
ENSMUSG00000033088	chr15	78772994	78773479
ENSMUSG00000075558	chr15	78826366	78826851
ENSMUSG00000075558	chr15	78831830	78832272
ENSMUSG00000022428	chr15	79489482	79490081
ENSMUSG00000042428	chr15	79991159	79991701
ENSMUSG00000047888	chr15	80534721	80535165
ENSMUSG00000022407	chr15	80680339	80680690
ENSMUSG00000042292	chr15	80917150	80917777
ENSMUSG00000022390	chr15	81576280	81576777
ENSMUSG00000022389	chr15	81632416	81633439
ENSMUSG00000068086	chr15	82285159	82285577
ENSMUSG00000058099	chr15	82839093	82839626
ENSMUSG00000016664	chr15	83302013	83302522
ENSMUSG00000041736	chr15	83396451	83396980
ENSMUSG00000036106	chr15	84472296	84472952
ENSMUSG00000016624	chr15	84686704	84687124
ENSMUSG00000036046	chr15	84806061	84806691
ENSMUSG00000065608	chr15	85483885	85484404
ENSMUSG00000065608	chr15	85501104	85501622
ENSMUSG00000065608	chr15	85508654	85509452
ENSMUSG00000065608	chr15	85533755	85534189
ENSMUSG00000016028	chr15	85869135	85869702
ENSMUSG00000035900	chr15	85899755	85900159
ENSMUSG00000034333	chr15	88582570	88582987
ENSMUSG00000035757	chr15	88919073	88919547
ENSMUSG00000022615	chr15	89203766	89204214
ENSMUSG00000036298	chr15	91450102	91450475
ENSMUSG00000036167	chr15	93293062	93293325
ENSMUSG00000044250	chr15	97134435	97134985

ENSMUSG00000022479	chr15	97745176	97745704
ENSMUSG00000052369	chr15	97780073	97780566
ENSMUSG00000022483	chr15	97814599	97815451
ENSMUSG00000022483	chr15	97815462	97816055
ENSMUSG00000022483	chr15	97816971	97817499
ENSMUSG00000022483	chr15	97817669	97818880
ENSMUSG00000022483	chr15	97818886	97820093
ENSMUSG00000022483	chr15	97821092	97821653
ENSMUSG00000022483	chr15	97825093	97825615
ENSMUSG00000022483	chr15	97826329	97826767
ENSMUSG00000022483	chr15	97826784	97827284
ENSMUSG00000022483	chr15	97828474	97829275
ENSMUSG00000003360	chr15	98493122	98493638
ENSMUSG00000022996	chr15	98607668	98608151
ENSMUSG00000043091	chr15	98862845	98863365
ENSMUSG00000001076	chr15	98918703	98919267
ENSMUSG00000023011	chr15	99343785	99344361
ENSMUSG00000043144	chr15	99428191	99428738
ENSMUSG00000037438	chr15	99733612	99734044
ENSMUSG00000000531	chr15	101051758	101052333
ENSMUSG00000075408	chr15	101124163	101124678
ENSMUSG00000037003	chr15	101936337	101936845
ENSMUSG00000023045	chr15	101980830	101981429
ENSMUSG00000076009	chr15	102502714	102503190
ENSMUSG00000000555	chr15	103196319	103196611
ENSMUSG00000022516	chr16	4912980	4913507
ENSMUSG00000022710	chr16	8723125	8723666
ENSMUSG00000038037	chr16	10782967	10783434
ENSMUSG00000037991	chr16	10843819	10844324
ENSMUSG00000082653	chr16	11618400	11618817
ENSMUSG00000022681	chr16	13818989	13819306
ENSMUSG00000013539	chr16	18345375	18345882
ENSMUSG00000041215	chr16	20141192	20141787
ENSMUSG00000003233	chr16	20516869	20517330
ENSMUSG00000013089	chr16	22428930	22429514
ENSMUSG00000013089	chr16	22439198	22439671
ENSMUSG00000084511	chr16	22547870	22548533
ENSMUSG00000022861	chr16	22661138	22661600
ENSMUSG00000038148	chr16	26447824	26448271
ENSMUSG00000047434	chr16	31017543	31017945
ENSMUSG00000022770	chr16	31551853	31552205
ENSMUSG00000022791	chr16	32644716	32645365
ENSMUSG00000035629	chr16	32867768	32868657
ENSMUSG00000022814	chr16	34181629	34182141
ENSMUSG00000022814	chr16	34212992	34213616
ENSMUSG00000061751	chr16	34562712	34563159
ENSMUSG00000022832	chr16	34628740	34629025
ENSMUSG00000022820	chr16	37689818	37690373
ENSMUSG00000036279	chr16	43549398	43550037
ENSMUSG00000052459	chr16	44136655	44137229
ENSMUSG00000022687	chr16	44523195	44523757
ENSMUSG00000022748	chr16	57627437	57628785
ENSMUSG00000022724	chr16	59471651	59471993
ENSMUSG00000032932	chr16	75767837	75768303
ENSMUSG00000022889	chr16	84735747	84736177
ENSMUSG00000056706	chr16	89586423	89586886
ENSMUSG00000022978	chr16	90730384	90730651
ENSMUSG00000039680	chr16	92105551	92106032

ENSMUSG00000022952	chr16	92778273	92778774
ENSMUSG00000076457	chr16	93299984	93300389
ENSMUSG00000022948	chr16	93604108	93604764
ENSMUSG00000040732	chr16	95794693	95795083
ENSMUSG00000084679	chr16	96826672	96827211
ENSMUSG00000050272	chr16	97218938	97219234
ENSMUSG00000014039	chr16	98073302	98074013
ENSMUSG00000023916	chr17	3224422	3224873
ENSMUSG00000023800	chr17	3398328	3398992
ENSMUSG00000023805	chr17	5975303	5975746
ENSMUSG00000023809	chr17	7374314	7374991
ENSMUSG00000071322	chr17	7465602	7466159
ENSMUSG00000023827	chr17	12311444	12312259
ENSMUSG00000014426	chr17	12511601	12512148
ENSMUSG00000023886	chr17	14416100	14416504
ENSMUSG00000046390	chr17	14683133	14683611
ENSMUSG00000079707	chr17	15209272	15209894
ENSMUSG00000051341	chr17	21669507	21670015
ENSMUSG00000041319	chr17	23810163	23810846
ENSMUSG00000002496	chr17	24769535	24769878
ENSMUSG00000024163	chr17	25073138	25073879
ENSMUSG00000025731	chr17	26011200	26011545
ENSMUSG00000037326	chr17	26122511	26123363
ENSMUSG00000057789	chr17	27172065	27172696
ENSMUSG00000042644	chr17	27188757	27189288
ENSMUSG00000042644	chr17	27211356	27211874
ENSMUSG00000042644	chr17	27212022	27212581
ENSMUSG00000024210	chr17	27316587	27317282
ENSMUSG00000044857	chr17	27382881	27383384
ENSMUSG00000052146	chr17	27774568	27774902
ENSMUSG00000024219	chr17	28082523	28083066
ENSMUSG00000038677	chr17	28286199	28286724
ENSMUSG00000024220	chr17	28313974	28314618
ENSMUSG00000002250	chr17	28360539	28360981
ENSMUSG00000002250	chr17	28372913	28374336
ENSMUSG00000007570	chr17	28451839	28452131
ENSMUSG00000053436	chr17	28827607	28828103
ENSMUSG00000053436	chr17	28828611	28829344
ENSMUSG00000073428	chr17	29168702	29169262
ENSMUSG00000044477	chr17	30294029	30294438
ENSMUSG00000052388	chr17	30472748	30473132
ENSMUSG00000024026	chr17	30746480	30746957
ENSMUSG00000024026	chr17	30749237	30749853
ENSMUSG00000061613	chr17	31795605	31796360
ENSMUSG00000061126	chr17	32611607	32612166
ENSMUSG00000057254	chr17	33034026	33034376
ENSMUSG00000024299	chr17	33662188	33662449
ENSMUSG00000002307	chr17	34046844	34047375
ENSMUSG00000073422	chr17	34164848	34165405
ENSMUSG00000024330	chr17	34189257	34189653
ENSMUSG00000024330	chr17	34189934	34190512
ENSMUSG00000024330	chr17	34190907	34191491
ENSMUSG00000024330	chr17	34194054	34194400
ENSMUSG00000024330	chr17	34194615	34196923
ENSMUSG00000024330	chr17	34199869	34200328
ENSMUSG00000024330	chr17	34200432	34201106
ENSMUSG00000024335	chr17	34258129	34258949
ENSMUSG00000015468	chr17	34705384	34705797

ENSMUSG00000059714	chr17	35966709	35967157
ENSMUSG00000001525	chr17	35971847	35972858
ENSMUSG00000038500	chr17	36133926	36134274
ENSMUSG00000045409	chr17	36407616	36408164
ENSMUSG00000036214	chr17	37096147	37096659
ENSMUSG00000041293	chr17	43389548	43389963
ENSMUSG00000023912	chr17	43803693	43804075
ENSMUSG00000023935	chr17	45611651	45612208
ENSMUSG00000023947	chr17	45693619	45694273
ENSMUSG00000023942	chr17	45732522	45732995
ENSMUSG00000036026	chr17	45822490	45823142
ENSMUSG00000023951	chr17	46166004	46166530
ENSMUSG00000012296	chr17	46407344	46407885
ENSMUSG00000012296	chr17	46417698	46418112
ENSMUSG00000073390	chr17	46481299	46481777
ENSMUSG00000067093	chr17	53429918	53430459
ENSMUSG00000057835	chr17	56017899	56018459
ENSMUSG00000003198	chr17	56031202	56031838
ENSMUSG00000062101	chr17	56084194	56085083
ENSMUSG00000013236	chr17	56598527	56599390
ENSMUSG00000040828	chr17	56795537	56796074
ENSMUSG00000002372	chr17	56808093	56808627
ENSMUSG00000024206	chr17	56970612	56971264
ENSMUSG00000061950	chr17	66135534	66136098
ENSMUSG00000024096	chr17	66234789	66235392
ENSMUSG00000034647	chr17	66410654	66411187
ENSMUSG00000073376	chr17	66707010	66707763
ENSMUSG00000024043	chr17	68439987	68440500
ENSMUSG00000024044	chr17	69515104	69515784
ENSMUSG00000047407	chr17	71196397	71196767
ENSMUSG00000024048	chr17	71349540	71349992
ENSMUSG00000024052	chr17	71532525	71532948
ENSMUSG00000041057	chr17	71947230	71948395
ENSMUSG00000039770	chr17	73147179	73147713
ENSMUSG00000024067	chr17	74715665	74716118
ENSMUSG00000024078	chr17	75244784	75245402
ENSMUSG00000037064	chr17	78617497	78618115
ENSMUSG00000056121	chr17	78787036	78787464
ENSMUSG00000024077	chr17	79135721	79136395
ENSMUSG00000024077	chr17	79169488	79170077
ENSMUSG00000024070	chr17	79420195	79420809
ENSMUSG00000024087	chr17	80181117	80181588
ENSMUSG00000024242	chr17	81110482	81110902
ENSMUSG00000079333	chr17	81814313	81814875
ENSMUSG00000024247	chr17	83301496	83303182
ENSMUSG00000032624	chr17	83719417	83719840
ENSMUSG00000065732	chr17	84706030	84706523
ENSMUSG00000065156	chr17	84763953	84764461
ENSMUSG00000065319	chr17	84985100	84985946
ENSMUSG00000024253	chr17	85026164	85026729
ENSMUSG00000036918	chr17	87682356	87682929
ENSMUSG00000036918	chr17	87714198	87714844
ENSMUSG00000036438	chr17	87872588	87873573
ENSMUSG00000061013	chr18	7004416	7005159
ENSMUSG00000073638	chr18	7404073	7404902
ENSMUSG00000037769	chr18	8695365	8695782
ENSMUSG00000073635	chr18	9858388	9859160
ENSMUSG00000024290	chr18	10181106	10181679

ENSMUSG00000024404	chr18	12287288	12287611
ENSMUSG00000024430	chr18	12884662	12885134
ENSMUSG00000024420	chr18	14130812	14131210
ENSMUSG00000036225	chr18	15295489	15296000
ENSMUSG00000024380	chr18	32719464	32719825
ENSMUSG00000034300	chr18	34922043	34922477
ENSMUSG00000024360	chr18	35090920	35091313
ENSMUSG00000043991	chr18	36440541	36440910
ENSMUSG00000001379	chr18	36838788	36839334
ENSMUSG00000024456	chr18	38088403	38088930
ENSMUSG00000056742	chr18	39704232	39704637
ENSMUSG00000024477	chr18	46439989	46440522
ENSMUSG00000037416	chr18	49991797	49992433
ENSMUSG00000024507	chr18	50308974	50309478
ENSMUSG00000024600	chr18	58715937	58716276
ENSMUSG00000043079	chr18	60772873	60773266
ENSMUSG00000043079	chr18	60805930	60806412
ENSMUSG00000024622	chr18	61314764	61315322
ENSMUSG00000024516	chr18	65926630	65927312
ENSMUSG00000053477	chr18	69707153	69707698
ENSMUSG00000024513	chr18	70760702	70761332
ENSMUSG00000024556	chr18	73974817	73975660
ENSMUSG00000035765	chr18	75178329	75179061
ENSMUSG00000025880	chr18	75545692	75546095
ENSMUSG00000052928	chr18	75856150	75856629
ENSMUSG00000052928	chr18	75857261	75857728
ENSMUSG00000052928	chr18	75896727	75897176
ENSMUSG00000079589	chr18	76256721	76257175
ENSMUSG00000059336	chr18	78359808	78360239
ENSMUSG00000024552	chr18	78435178	78435806
ENSMUSG00000053950	chr18	80331673	80332104
ENSMUSG00000033323	chr18	80748731	80749248
ENSMUSG00000033016	chr18	80867261	80867725
ENSMUSG00000033016	chr18	80904953	80905360
ENSMUSG00000044356	chr18	84758855	84759231
ENSMUSG00000073517	chr18	84889697	84890128
ENSMUSG00000023066	chr18	89140951	89141393
ENSMUSG00000024831	chr19	3282614	3283176
ENSMUSG00000024908	chr19	3600531	3600934
ENSMUSG00000024913	chr19	3681397	3681959
ENSMUSG00000045098	chr19	3767326	3767790
ENSMUSG00000001750	chr19	3907203	3907790
ENSMUSG00000037916	chr19	4012642	4013171
ENSMUSG00000024845	chr19	4126688	4127110
ENSMUSG00000045826	chr19	4154084	4154597
ENSMUSG00000024830	chr19	4163198	4163596
ENSMUSG00000006464	chr19	4895259	4895633
ENSMUSG00000024891	chr19	5023637	5024051
ENSMUSG00000075264	chr19	5117287	5117772
ENSMUSG00000024912	chr19	5444413	5445799
ENSMUSG00000024909	chr19	5475347	5475932
ENSMUSG00000054874	chr19	5684490	5684990
ENSMUSG00000024941	chr19	5800727	5801521
ENSMUSG00000024941	chr19	5801953	5802329
ENSMUSG00000043488	chr19	5843477	5844673
ENSMUSG00000043488	chr19	5844752	5845641
ENSMUSG00000024807	chr19	6047448	6047847
ENSMUSG00000024790	chr19	6116671	6117153

ENSMUSG00000024786	chr19	6181733	6182065
ENSMUSG00000079467	chr19	6364461	6365134
ENSMUSG00000024952	chr19	6915111	6915577
ENSMUSG00000024952	chr19	6922206	6923016
ENSMUSG00000056612	chr19	7050217	7050692
ENSMUSG00000036278	chr19	7156069	7156671
ENSMUSG00000047787	chr19	7205710	7206165
ENSMUSG00000024767	chr19	7281194	7281644
ENSMUSG00000024759	chr19	7568575	7569027
ENSMUSG00000069833	chr19	9062907	9063666
ENSMUSG00000069833	chr19	9064792	9065258
ENSMUSG00000069833	chr19	9066881	9067561
ENSMUSG00000024740	chr19	10680440	10681014
ENSMUSG00000039982	chr19	12575926	12576290
ENSMUSG00000039982	chr19	12592827	12593080
ENSMUSG00000039126	chr19	17217774	17218309
ENSMUSG00000024750	chr19	21346280	21346824
ENSMUSG00000024754	chr19	21824870	21825233
ENSMUSG00000024754	chr19	21852991	21853643
ENSMUSG00000024754	chr19	21861671	21862176
ENSMUSG00000052942	chr19	28702529	28702961
ENSMUSG00000024785	chr19	29186855	29187453
ENSMUSG00000024887	chr19	32284952	32285712
ENSMUSG00000067297	chr19	34715193	34715735
ENSMUSG00000033519	chr19	34952384	34952898
ENSMUSG00000024803	chr19	36288297	36288671
ENSMUSG00000048612	chr19	38102353	38102884
ENSMUSG00000054200	chr19	38175037	38175531
ENSMUSG00000025001	chr19	39004545	39005372
ENSMUSG00000025008	chr19	40686372	40686972
ENSMUSG00000025016	chr19	41334997	41335654
ENSMUSG00000034321	chr19	42007551	42007985
ENSMUSG00000044345	chr19	42221641	42222092
ENSMUSG00000025185	chr19	42694689	42695186
ENSMUSG00000074852	chr19	43433176	43433720
ENSMUSG00000045472	chr19	46592427	46592808
ENSMUSG00000025036	chr19	46648246	46648621
ENSMUSG00000033033	chr19	47211287	47211776
ENSMUSG00000025064	chr19	47722324	47722881
ENSMUSG00000025064	chr19	47723304	47724092
ENSMUSG00000025064	chr19	47724170	47724999
ENSMUSG00000025064	chr19	47725107	47725591
ENSMUSG00000025064	chr19	47726447	47726950
ENSMUSG00000025064	chr19	47727549	47728156
ENSMUSG00000025064	chr19	47731259	47731777
ENSMUSG00000025064	chr19	47734169	47734674
ENSMUSG00000025064	chr19	47734996	47735553
ENSMUSG00000046585	chr19	48012316	48012908
ENSMUSG00000033717	chr19	54118846	54119603
ENSMUSG00000074769	chr19	56096801	56097654
ENSMUSG00000025075	chr19	56362782	56363314
ENSMUSG00000035283	chr19	56753861	56754837
ENSMUSG00000078103	chr19	57115547	57116352
ENSMUSG00000025085	chr19	57271932	57272358
ENSMUSG00000033478	chr19	57456015	57456575
ENSMUSG00000024993	chr19	60887285	60887640
ENSMUSG00000026662	chr2	4801890	4802379
ENSMUSG00000039145	chr2	5629260	5629730

ENSMUSG00000039145	chr2	5635154	5635545
ENSMUSG00000081202	chr2	9396048	9396401
ENSMUSG00000079603	chr2	9614787	9615158
ENSMUSG00000061186	chr2	10236305	10236793
ENSMUSG00000049630	chr2	12931869	12932468
ENSMUSG00000026728	chr2	13493771	13494392
ENSMUSG00000026728	chr2	13500200	13500564
ENSMUSG00000083281	chr2	19029598	19029978
ENSMUSG00000043415	chr2	19579825	19580167
ENSMUSG00000006476	chr2	24909949	24911080
ENSMUSG00000036352	chr2	25877174	25877534
ENSMUSG00000026930	chr2	26181180	26181914
ENSMUSG00000026837	chr2	27742650	27744488
ENSMUSG00000026837	chr2	27813836	27814342
ENSMUSG00000026837	chr2	27815683	27816205
ENSMUSG00000026837	chr2	27820642	27821450
ENSMUSG00000026837	chr2	27824999	27825597
ENSMUSG00000026837	chr2	27829711	27830554
ENSMUSG00000026837	chr2	27835805	27836147
ENSMUSG00000026837	chr2	27836181	27836955
ENSMUSG00000026835	chr2	27938289	27938766
ENSMUSG00000082788	chr2	28955875	28956904
ENSMUSG00000039844	chr2	29511329	29511880
ENSMUSG00000039826	chr2	29635755	29636282
ENSMUSG00000065485	chr2	29701489	29702003
ENSMUSG00000047363	chr2	30456497	30456875
ENSMUSG00000084143	chr2	30780663	30781116
ENSMUSG00000026848	chr2	30798086	30798729
ENSMUSG00000026851	chr2	30837142	30838004
ENSMUSG00000055632	chr2	31151613	31152239
ENSMUSG00000055632	chr2	31153871	31154197
ENSMUSG00000039254	chr2	32091691	32092338
ENSMUSG00000044627	chr2	32143150	32143596
ENSMUSG00000065554	chr2	32159955	32160736
ENSMUSG00000026819	chr2	32306370	32307009
ENSMUSG00000026796	chr2	32725788	32726330
ENSMUSG00000081244	chr2	33573268	33573685
ENSMUSG00000081244	chr2	33616684	33617164
ENSMUSG00000038740	chr2	33676958	33677638
ENSMUSG00000038718	chr2	34122061	34122581
ENSMUSG00000035778	chr2	35300904	35301364
ENSMUSG00000026889	chr2	35991805	35992303
ENSMUSG00000083849	chr2	44381879	44382372
ENSMUSG00000026872	chr2	44972852	44973161
ENSMUSG00000069495	chr2	49306254	49306675
ENSMUSG00000026765	chr2	49599909	49600616
ENSMUSG00000036249	chr2	51790223	51790763
ENSMUSG00000083287	chr2	53717784	53718158
ENSMUSG00000060988	chr2	54420141	54420584
ENSMUSG00000026827	chr2	57090234	57090616
ENSMUSG00000035168	chr2	59450291	59450704
ENSMUSG00000026971	chr2	60664343	60664901
ENSMUSG00000026970	chr2	60801086	60801617
ENSMUSG00000063145	chr2	69484712	69485310
ENSMUSG00000027086	chr2	69551362	69551920
ENSMUSG00000027010	chr2	71205410	71205844
ENSMUSG00000041911	chr2	71337955	71338319
ENSMUSG00000015839	chr2	75541343	75541856

ENSMUSG00000027079	chr2	84567538	84567983
ENSMUSG00000034059	chr2	84575215	84575890
ENSMUSG00000002105	chr2	90909932	90910630
ENSMUSG00000081248	chr2	91228401	91228786
ENSMUSG00000027230	chr2	91851942	91852596
ENSMUSG00000027230	chr2	91856277	91857673
ENSMUSG00000027220	chr2	92755041	92755525
ENSMUSG00000075025	chr2	93291270	93291749
ENSMUSG00000050587	chr2	97318240	97318772
ENSMUSG00000082972	chr2	100866389	100866757
ENSMUSG00000082328	chr2	101970656	101971007
ENSMUSG00000027188	chr2	102397139	102397468
ENSMUSG00000005087	chr2	102796088	102796583
ENSMUSG00000032724	chr2	103407109	103407699
ENSMUSG00000027185	chr2	103601162	103601660
ENSMUSG00000005973	chr2	105239048	105239570
ENSMUSG00000027130	chr2	112105366	112105862
ENSMUSG00000074934	chr2	113622012	113622394
ENSMUSG00000027347	chr2	117250202	117250759
ENSMUSG00000027347	chr2	117441924	117442263
ENSMUSG00000027324	chr2	118860376	118860830
ENSMUSG00000027315	chr2	119067869	119068255
ENSMUSG00000034216	chr2	119114107	119114531
ENSMUSG00000046110	chr2	121283094	121283733
ENSMUSG00000033411	chr2	121781344	121781716
ENSMUSG00000033268	chr2	122163814	122164259
ENSMUSG00000074876	chr2	122456119	122456460
ENSMUSG00000027395	chr2	128919633	128919971
ENSMUSG00000060029	chr2	130389606	130390354
ENSMUSG00000068264	chr2	131035824	131036337
ENSMUSG00000027339	chr2	131831859	131832347
ENSMUSG00000027276	chr2	137434190	137434600
ENSMUSG00000039033	chr2	139892225	139892602
ENSMUSG00000027422	chr2	143835329	143836248
ENSMUSG00000082480	chr2	143990718	143991056
ENSMUSG00000063873	chr2	145039526	145040083
ENSMUSG00000037110	chr2	146337969	146338338
ENSMUSG00000053545	chr2	147001307	147001609
ENSMUSG00000053916	chr2	150864975	150865602
ENSMUSG00000032802	chr2	151931414	151931775
ENSMUSG00000074698	chr2	152062048	152062411
ENSMUSG00000038467	chr2	154482478	154482915
ENSMUSG00000074656	chr2	154718306	154718769
ENSMUSG00000038383	chr2	155183078	155183403
ENSMUSG00000038324	chr2	155517945	155518497
ENSMUSG00000074647	chr2	155678547	155678864
ENSMUSG00000038116	chr2	156070724	156071134
ENSMUSG00000067818	chr2	156600370	156600940
ENSMUSG00000055485	chr2	156899560	156900005
ENSMUSG00000055485	chr2	156904665	156904951
ENSMUSG00000082329	chr2	157359364	157359842
ENSMUSG00000018322	chr2	163934257	163934772
ENSMUSG00000039873	chr2	164659014	164659561
ENSMUSG00000006800	chr2	166126782	166127145
ENSMUSG00000039621	chr2	166555318	166555683
ENSMUSG00000017969	chr2	167057609	167058069
ENSMUSG00000042821	chr2	167363678	167364282
ENSMUSG00000081511	chr2	167395547	167396085

ENSMUSG00000081511	chr2	167415833	167416302
ENSMUSG00000019755	chr2	167486251	167486762
ENSMUSG00000056501	chr2	167515289	167515688
ENSMUSG00000006462	chr2	167517361	167517930
ENSMUSG00000006462	chr2	167527509	167528041
ENSMUSG00000027540	chr2	167776715	167777199
ENSMUSG00000008999	chr2	172721175	172721465
ENSMUSG00000027569	chr2	180316189	180316687
ENSMUSG00000027570	chr2	180333368	180333886
ENSMUSG00000027570	chr2	180335401	180335778
ENSMUSG00000027570	chr2	180335972	180336863
ENSMUSG00000027570	chr2	180344446	180345165
ENSMUSG00000027570	chr2	180345759	180346359
ENSMUSG00000027570	chr2	180347369	180348038
ENSMUSG00000074728	chr3	5210247	5210730
ENSMUSG00000040374	chr3	5576282	5576691
ENSMUSG00000027555	chr3	14629058	14629423
ENSMUSG00000039286	chr3	27607835	27608404
ENSMUSG00000043164	chr3	27766378	27766637
ENSMUSG00000027695	chr3	28006242	28007127
ENSMUSG00000037730	chr3	30501032	30501420
ENSMUSG00000037643	chr3	30922116	30922642
ENSMUSG00000027665	chr3	32296416	32296954
ENSMUSG00000027708	chr3	35824852	35825285
ENSMUSG00000054779	chr3	37269211	37269805
ENSMUSG00000046743	chr3	38642336	38642824
ENSMUSG00000023087	chr3	51028263	51028785
ENSMUSG00000070490	chr3	52288060	52288500
ENSMUSG00000048332	chr3	53008037	53008558
ENSMUSG00000048332	chr3	53014470	53015103
ENSMUSG00000027750	chr3	54162086	54162991
ENSMUSG00000036580	chr3	54902867	54903238
ENSMUSG00000027806	chr3	58221037	58221396
ENSMUSG00000027822	chr3	63768240	63768840
ENSMUSG00000074580	chr3	65333050	65333493
ENSMUSG00000027832	chr3	65966973	65967368
ENSMUSG00000027832	chr3	66024750	66025209
ENSMUSG00000047557	chr3	67267331	67267749
ENSMUSG00000027788	chr3	69830387	69830851
ENSMUSG00000027995	chr3	83645114	83645569
ENSMUSG00000027995	chr3	83649403	83649902
ENSMUSG00000027993	chr3	84122983	84123538
ENSMUSG00000041842	chr3	84283417	84283877
ENSMUSG00000031286	chr3	85676478	85676988
ENSMUSG00000049013	chr3	85798672	85799113
ENSMUSG00000028073	chr3	87568439	87568816
ENSMUSG00000028063	chr3	88299421	88299971
ENSMUSG00000028059	chr3	88423644	88424012
ENSMUSG00000068921	chr3	88754818	88755110
ENSMUSG00000027937	chr3	90035015	90035633
ENSMUSG00000001018	chr3	90299653	90300307
ENSMUSG00000001021	chr3	90404629	90405114
ENSMUSG00000001025	chr3	90416664	90417132
ENSMUSG00000015943	chr3	96000786	96001213
ENSMUSG00000038170	chr3	97671851	97672305
ENSMUSG00000027864	chr3	100882005	100882425
ENSMUSG00000027859	chr3	102165999	102166526
ENSMUSG00000000562	chr3	105697150	105697578

ENSMUSG00000027901	chr3	106297541	106298003
ENSMUSG00000014599	chr3	107546892	107547534
ENSMUSG00000027966	chr3	113734720	113735207
ENSMUSG00000027966	chr3	113736823	113737164
ENSMUSG00000027966	chr3	113826963	113827431
ENSMUSG00000027966	chr3	113860927	113861210
ENSMUSG00000027966	chr3	113867209	113867514
ENSMUSG00000027966	chr3	113884084	113884448
ENSMUSG00000027966	chr3	113888736	113888964
ENSMUSG00000027966	chr3	113896676	113897243
ENSMUSG00000028125	chr3	121793973	121794679
ENSMUSG00000028121	chr3	122212450	122212853
ENSMUSG00000050315	chr3	122842924	122843455
ENSMUSG00000050315	chr3	122938922	122939498
ENSMUSG00000039234	chr3	122999445	122999913
ENSMUSG00000053819	chr3	126422805	126423239
ENSMUSG00000078587	chr3	126627215	126627572
ENSMUSG00000019232	chr3	130266955	130267337
ENSMUSG00000004127	chr3	137806334	137806922
ENSMUSG00000028149	chr3	138737683	138738069
ENSMUSG00000028266	chr3	143927485	143927783
ENSMUSG00000028197	chr3	145024638	145025058
ENSMUSG00000074182	chr3	145104252	145104534
ENSMUSG00000074182	chr3	145136916	145137555
ENSMUSG00000074182	chr3	145149118	145149406
ENSMUSG00000074182	chr3	145170398	145170951
ENSMUSG00000028194	chr3	145453076	145453681
ENSMUSG00000054942	chr3	151971577	151971924
ENSMUSG00000028201	chr3	154077423	154079190
ENSMUSG00000028182	chr3	154756407	154756792
ENSMUSG00000049119	chr4	5571367	5571891
ENSMUSG00000064698	chr4	9672669	9673148
ENSMUSG00000028218	chr4	12098940	12099387
ENSMUSG00000040550	chr4	14753639	14753923
ENSMUSG00000028261	chr4	24825162	24825526
ENSMUSG00000028284	chr4	32050562	32051411
ENSMUSG00000028277	chr4	33118237	33118599
ENSMUSG00000039967	chr4	34833698	34834152
ENSMUSG00000081720	chr4	40573352	40573731
ENSMUSG00000028427	chr4	40987277	40987826
ENSMUSG00000036002	chr4	43051558	43052635
ENSMUSG00000028464	chr4	43535181	43535732
ENSMUSG00000028464	chr4	43537438	43537722
ENSMUSG00000039813	chr4	46674268	46674773
ENSMUSG00000039809	chr4	46857877	46858104
ENSMUSG00000028339	chr4	47231680	47232259
ENSMUSG00000007613	chr4	47299651	47300277
ENSMUSG00000007613	chr4	47305880	47306374
ENSMUSG00000028343	chr4	48292235	48293206
ENSMUSG00000028347	chr4	48598979	48599473
ENSMUSG00000028412	chr4	53453966	53454378
ENSMUSG00000038764	chr4	57307528	57308226
ENSMUSG00000028367	chr4	57968449	57969260
ENSMUSG00000028389	chr4	61869355	61870088
ENSMUSG00000066149	chr4	62069242	62069923
ENSMUSG00000028394	chr4	62185641	62186476
ENSMUSG00000077161	chr4	62396834	62397423
ENSMUSG00000045672	chr4	62886682	62887308

ENSMUSG00000070102	chr4	62914388	62915078
ENSMUSG00000039196	chr4	62961534	62962035
ENSMUSG00000039196	chr4	62962057	62963188
ENSMUSG00000039196	chr4	62966012	62966438
ENSMUSG00000039196	chr4	62970548	62970916
ENSMUSG00000039196	chr4	62977946	62979331
ENSMUSG00000039196	chr4	62979806	62980860
ENSMUSG00000039158	chr4	63071261	63071724
ENSMUSG00000039105	chr4	63205996	63206687
ENSMUSG00000039105	chr4	63208753	63209256
ENSMUSG00000048706	chr4	80564571	80565116
ENSMUSG00000052407	chr4	83170634	83171067
ENSMUSG00000052407	chr4	83171421	83171981
ENSMUSG00000028497	chr4	88125917	88126367
ENSMUSG00000028572	chr4	95601341	95601726
ENSMUSG00000035305	chr4	99900808	99901328
ENSMUSG00000082928	chr4	100906955	100907561
ENSMUSG00000028601	chr4	107838088	107838503
ENSMUSG00000028559	chr4	108820108	108820711
ENSMUSG00000084673	chr4	111187678	111188076
ENSMUSG00000070867	chr4	114092785	114093186
ENSMUSG00000055210	chr4	114582030	114582343
ENSMUSG00000063882	chr4	115745312	115745821
ENSMUSG00000028693	chr4	116299512	116299845
ENSMUSG00000028540	chr4	117564477	117564879
ENSMUSG00000028635	chr4	119721617	119722090
ENSMUSG00000028626	chr4	120713331	120713976
ENSMUSG00000028626	chr4	120717214	120717573
ENSMUSG00000028626	chr4	120724810	120725305
ENSMUSG00000028626	chr4	120726246	120726729
ENSMUSG00000082310	chr4	123496384	123496782
ENSMUSG00000032643	chr4	124377510	124377854
ENSMUSG00000028871	chr4	124664156	124664564
ENSMUSG00000028869	chr4	124694653	124695129
ENSMUSG00000050212	chr4	125809375	125809916
ENSMUSG00000040690	chr4	129734827	129735988
ENSMUSG00000040690	chr4	129737418	129738013
ENSMUSG00000040690	chr4	129738335	129738838
ENSMUSG00000040690	chr4	129744035	129744582
ENSMUSG00000040690	chr4	129745369	129745918
ENSMUSG00000028779	chr4	129756732	129757224
ENSMUSG00000028779	chr4	129763151	129763500
ENSMUSG00000028779	chr4	129773391	129774029
ENSMUSG00000028779	chr4	129774823	129775279
ENSMUSG00000028779	chr4	129777815	129778285
ENSMUSG00000028776	chr4	129861561	129861957
ENSMUSG00000028581	chr4	130452184	130452487
ENSMUSG00000028911	chr4	131429859	131430290
ENSMUSG00000070717	chr4	131586849	131587387
ENSMUSG00000028886	chr4	132208777	132209207
ENSMUSG00000028854	chr4	132927329	132927589
ENSMUSG00000056596	chr4	133053800	133054275
ENSMUSG00000037188	chr4	135100841	135101290
ENSMUSG00000007872	chr4	135673261	135673587
ENSMUSG00000036896	chr4	136447555	136448769
ENSMUSG00000070667	chr4	136943898	136944463
ENSMUSG00000028763	chr4	137025504	137026000
ENSMUSG00000006445	chr4	140842789	140843181

ENSMUSG00000057637	chr4	142807196	142807671
ENSMUSG00000029020	chr4	147292448	147293496
ENSMUSG00000028979	chr4	147978816	147979105
ENSMUSG00000042804	chr4	151636424	151636959
ENSMUSG00000039577	chr4	151865403	151865872
ENSMUSG00000039410	chr4	153896033	153896303
ENSMUSG00000051276	chr4	154128621	154128936
ENSMUSG00000029050	chr4	154529208	154529764
ENSMUSG00000029050	chr4	154610008	154610405
ENSMUSG00000042116	chr4	155144000	155144379
ENSMUSG00000040302	chr5	3603195	3603484
ENSMUSG00000046798	chr5	5514739	5515088
ENSMUSG00000062038	chr5	13229790	13230288
ENSMUSG00000080293	chr5	14823640	14823954
ENSMUSG00000028780	chr5	17195459	17196140
ENSMUSG00000040003	chr5	19097108	19097401
ENSMUSG00000028771	chr5	20560664	20561254
ENSMUSG00000029004	chr5	22963034	22963590
ENSMUSG00000023353	chr5	23958178	23958804
ENSMUSG00000076387	chr5	24096963	24097671
ENSMUSG00000028949	chr5	24128359	24128818
ENSMUSG00000028938	chr5	24690893	24691427
ENSMUSG00000038828	chr5	31152349	31152918
ENSMUSG00000029162	chr5	31221434	31221990
ENSMUSG00000029145	chr5	31495302	31495828
ENSMUSG00000078181	chr5	31939133	31939587
ENSMUSG00000073058	chr5	32435091	32435561
ENSMUSG00000082305	chr5	33015750	33016382
ENSMUSG00000037373	chr5	33606807	33607293
ENSMUSG00000037373	chr5	33616947	33617360
ENSMUSG00000037339	chr5	33970733	33971138
ENSMUSG00000019295	chr5	33999828	34000332
ENSMUSG00000059866	chr5	34855388	34855908
ENSMUSG00000029094	chr5	36240720	36241102
ENSMUSG00000029190	chr5	37086574	37086906
ENSMUSG00000029190	chr5	37103906	37104253
ENSMUSG00000029092	chr5	45503296	45503732
ENSMUSG00000015880	chr5	46060485	46061019
ENSMUSG00000029178	chr5	64970884	64971227
ENSMUSG00000029178	chr5	65194581	65195124
ENSMUSG00000037822	chr5	65883315	65883953
ENSMUSG00000054598	chr5	66396277	66396818
ENSMUSG00000070733	chr5	73646961	73647575
ENSMUSG00000029228	chr5	75050461	75051247
ENSMUSG00000029231	chr5	75548720	75549491
ENSMUSG00000029231	chr5	75552661	75553127
ENSMUSG00000006262	chr5	89128527	89128937
ENSMUSG00000029366	chr5	89193842	89194295
ENSMUSG00000035505	chr5	90652957	90653314
ENSMUSG00000029413	chr5	92717529	92718014
ENSMUSG00000029415	chr5	92738500	92739288
ENSMUSG00000047963	chr5	93021075	93021477
ENSMUSG00000058013	chr5	93521818	93522291
ENSMUSG00000058013	chr5	93522732	93523084
ENSMUSG00000029484	chr5	97215243	97215705
ENSMUSG00000084126	chr5	104008570	104009106
ENSMUSG00000064629	chr5	104267609	104268162
ENSMUSG00000034528	chr5	104410225	104410567

ENSMUSG00000029310	chr5	104484025	104484482
ENSMUSG00000049606	chr5	107125807	107126158
ENSMUSG00000084218	chr5	107569560	107569866
ENSMUSG00000062234	chr5	109058634	109059203
ENSMUSG00000033316	chr5	110992506	110992896
ENSMUSG00000072734	chr5	111586739	111587144
ENSMUSG00000070576	chr5	111846180	111846497
ENSMUSG00000070576	chr5	111849480	111850485
ENSMUSG00000052848	chr5	111960201	111960707
ENSMUSG00000029352	chr5	113528670	113529113
ENSMUSG00000051339	chr5	113588711	113589179
ENSMUSG00000051339	chr5	113594562	113594935
ENSMUSG00000053334	chr5	114188903	114189318
ENSMUSG00000025825	chr5	114222543	114222871
ENSMUSG00000065320	chr5	114380972	114381425
ENSMUSG00000044339	chr5	114577938	114578402
ENSMUSG00000011884	chr5	115141207	115141644
ENSMUSG00000046562	chr5	115583602	115584096
ENSMUSG00000046562	chr5	115586179	115587027
ENSMUSG00000048578	chr5	115606878	115607334
ENSMUSG00000067274	chr5	116010582	116011307
ENSMUSG00000018604	chr5	120069785	120070203
ENSMUSG00000029601	chr5	121039086	121039381
ENSMUSG00000001166	chr5	121262425	121263023
ENSMUSG00000062438	chr5	121956735	121957307
ENSMUSG00000042594	chr5	122280557	122280980
ENSMUSG00000083909	chr5	122751843	122752343
ENSMUSG00000056735	chr5	123400422	123400870
ENSMUSG00000054434	chr5	123526092	123526621
ENSMUSG00000029449	chr5	123583735	123584178
ENSMUSG00000062946	chr5	123588426	123589026
ENSMUSG00000049550	chr5	124133980	124134325
ENSMUSG00000000915	chr5	124423232	124423595
ENSMUSG00000066278	chr5	124457116	124457723
ENSMUSG00000029394	chr5	124803260	124803723
ENSMUSG00000029478	chr5	125725877	125726424
ENSMUSG00000008348	chr5	125885561	125885941
ENSMUSG00000029439	chr5	130007172	130007490
ENSMUSG00000025534	chr5	130465180	130465716
ENSMUSG00000025534	chr5	130475016	130475483
ENSMUSG00000060371	chr5	130813476	130813883
ENSMUSG00000076320	chr5	134913268	134913896
ENSMUSG00000023104	chr5	135058453	135058907
ENSMUSG00000029675	chr5	135244243	135244913
ENSMUSG00000053293	chr5	135941588	135942137
ENSMUSG00000039886	chr5	136211561	136212024
ENSMUSG00000039860	chr5	136276750	136277309
ENSMUSG00000051391	chr5	136410631	136411239
ENSMUSG00000039771	chr5	136591986	136592444
ENSMUSG00000029719	chr5	138069506	138069864
ENSMUSG00000029725	chr5	138220793	138221189
ENSMUSG00000056014	chr5	139063699	139064136
ENSMUSG00000056493	chr5	142878660	142879253
ENSMUSG00000039477	chr5	143582562	143583450
ENSMUSG00000029580	chr5	143667926	143668445
ENSMUSG00000029580	chr5	143682398	143682982
ENSMUSG00000029586	chr5	143956387	143956901
ENSMUSG00000066621	chr5	144968853	144969443

ENSMUSG00000038780	chr5	145659333	145659841
ENSMUSG00000041313	chr5	149210350	149210917
ENSMUSG00000079069	chr5	149761611	149761943
ENSMUSG00000079069	chr5	149762012	149762595
ENSMUSG00000029657	chr5	150439186	150439549
ENSMUSG00000016128	chr5	151967120	151967412
ENSMUSG00000047735	chr6	3335684	3336037
ENSMUSG00000029661	chr6	4455625	4456148
ENSMUSG00000029661	chr6	4459413	4459943
ENSMUSG00000029661	chr6	4460067	4461452
ENSMUSG00000029661	chr6	4461578	4461891
ENSMUSG00000029661	chr6	4464800	4466594
ENSMUSG00000029661	chr6	4466604	4467267
ENSMUSG00000029661	chr6	4468418	4471901
ENSMUSG00000029661	chr6	4472330	4475649
ENSMUSG00000029661	chr6	4476575	4477502
ENSMUSG00000029661	chr6	4477908	4480595
ENSMUSG00000029661	chr6	4480650	4483037
ENSMUSG00000029661	chr6	4483282	4488500
ENSMUSG00000029661	chr6	4489030	4489906
ENSMUSG00000048365	chr6	7972001	7972495
ENSMUSG00000048365	chr6	8036730	8037199
ENSMUSG00000079679	chr6	13322190	13322625
ENSMUSG00000052419	chr6	13821036	13821906
ENSMUSG00000007655	chr6	17290630	17291021
ENSMUSG00000029672	chr6	22450297	22450902
ENSMUSG00000029701	chr6	29120931	29121417
ENSMUSG00000079654	chr6	29128933	29129241
ENSMUSG00000073209	chr6	30930225	30930769
ENSMUSG00000065604	chr6	31021112	31021626
ENSMUSG00000065604	chr6	31032594	31033069
ENSMUSG00000065604	chr6	31035860	31036374
ENSMUSG00000065604	chr6	31037495	31038077
ENSMUSG00000025609	chr6	31271929	31272397
ENSMUSG00000025609	chr6	31282017	31282747
ENSMUSG00000025609	chr6	31286433	31287006
ENSMUSG00000038759	chr6	35127452	35127980
ENSMUSG00000019689	chr6	38482897	38484054
ENSMUSG00000002413	chr6	39669489	39669802
ENSMUSG00000029860	chr6	42303272	42304304
ENSMUSG00000029860	chr6	42305787	42306097
ENSMUSG00000029851	chr6	42626058	42626581
ENSMUSG00000064945	chr6	47689432	47690397
ENSMUSG00000044867	chr6	48692169	48692608
ENSMUSG00000029814	chr6	49168116	49168439
ENSMUSG00000029821	chr6	50211681	50212111
ENSMUSG00000029776	chr6	52589863	52590266
ENSMUSG00000029781	chr6	56827503	56827990
ENSMUSG00000049232	chr6	59159139	59159731
ENSMUSG00000018341	chr6	67380843	67381149
ENSMUSG00000037621	chr6	72185294	72185704
ENSMUSG00000053907	chr6	72389282	72389722
ENSMUSG00000056698	chr6	72548143	72548726
ENSMUSG00000055799	chr6	72738189	72738571
ENSMUSG00000065072	chr6	73102127	73102543
ENSMUSG00000068329	chr6	83004219	83004807
ENSMUSG00000034777	chr6	83652186	83652528
ENSMUSG00000014748	chr6	83725617	83726496

ENSMUSG00000033788	chr6	84046052	84046539
ENSMUSG00000071345	chr6	85081608	85082015
ENSMUSG00000033420	chr6	87281286	87281860
ENSMUSG00000033420	chr6	87283728	87284295
ENSMUSG00000015053	chr6	88154213	88154862
ENSMUSG00000055403	chr6	89014991	89015577
ENSMUSG00000056643	chr6	90265673	90266210
ENSMUSG00000034312	chr6	90656121	90656434
ENSMUSG00000064080	chr6	91151598	91152123
ENSMUSG00000034203	chr6	91423317	91423808
ENSMUSG00000030096	chr6	91567034	91567645
ENSMUSG00000030096	chr6	91596895	91597605
ENSMUSG00000030059	chr6	97128664	97129134
ENSMUSG00000044086	chr6	97297974	97298306
ENSMUSG00000030067	chr6	99166579	99166928
ENSMUSG00000072872	chr6	100236344	100236868
ENSMUSG00000079446	chr6	100655032	100655540
ENSMUSG00000043024	chr6	108520653	108521192
ENSMUSG00000078282	chr6	112773254	112773698
ENSMUSG00000030257	chr6	112856009	112856465
ENSMUSG00000030286	chr6	113481420	113481872
ENSMUSG00000030314	chr6	114592866	114593472
ENSMUSG00000030315	chr6	114861345	114861931
ENSMUSG00000030316	chr6	114992643	114993021
ENSMUSG00000054488	chr6	117410895	117411524
ENSMUSG00000042087	chr6	117866945	117867277
ENSMUSG00000072623	chr6	118427790	118428290
ENSMUSG00000041477	chr6	118852905	118853340
ENSMUSG00000041477	chr6	119057864	119058369
ENSMUSG00000030019	chr6	119430069	119431092
ENSMUSG00000030170	chr6	119471706	119472423
ENSMUSG00000040669	chr6	122290170	122290598
ENSMUSG00000072772	chr6	124690512	124690866
ENSMUSG00000030122	chr6	124870148	124870658
ENSMUSG00000038271	chr6	125096756	125097182
ENSMUSG00000067702	chr6	125241249	125241694
ENSMUSG00000038167	chr6	125327453	125328244
ENSMUSG00000001930	chr6	125570731	125571233
ENSMUSG00000030352	chr6	128093711	128094303
ENSMUSG00000030199	chr6	133997186	133997681
ENSMUSG00000042992	chr6	134646028	134646320
ENSMUSG00000030204	chr6	134933517	134933984
ENSMUSG00000030204	chr6	134961302	134962007
ENSMUSG00000079274	chr6	137467973	137468530
ENSMUSG00000015766	chr6	137508666	137509152
ENSMUSG00000084477	chr6	140439591	140439980
ENSMUSG00000030232	chr6	140591453	140591989
ENSMUSG00000079262	chr6	142168289	142168706
ENSMUSG00000080637	chr6	144589077	144589707
ENSMUSG00000072677	chr6	144735458	144735932
ENSMUSG00000030268	chr6	145024238	145024783
ENSMUSG00000043541	chr6	145135726	145136276
ENSMUSG00000040250	chr6	146526361	146526972
ENSMUSG00000040234	chr6	146582671	146583738
ENSMUSG00000040187	chr6	146716829	146717219
ENSMUSG00000040187	chr6	146725077	146725539
ENSMUSG00000030292	chr6	146773478	146774073
ENSMUSG00000040163	chr6	146934972	146935488

ENSMUSG00000032712	chr6	149258193	149258570
ENSMUSG00000054753	chr7	3170204	3170585
ENSMUSG00000019254	chr7	4453568	4454101
ENSMUSG00000030386	chr7	13063811	13064267
ENSMUSG00000054715	chr7	13482833	13483242
ENSMUSG00000033916	chr7	13619783	13620441
ENSMUSG00000041560	chr7	16533772	16534135
ENSMUSG00000006024	chr7	16683894	16684202
ENSMUSG00000006019	chr7	16791327	16791854
ENSMUSG00000044030	chr7	19589211	19589447
ENSMUSG00000044030	chr7	19589624	19590313
ENSMUSG00000030410	chr7	19660866	19661253
ENSMUSG00000040857	chr7	26033481	26033783
ENSMUSG00000040390	chr7	28459688	28460158
ENSMUSG00000047586	chr7	29355161	29355689
ENSMUSG00000053522	chr7	29634931	29635371
ENSMUSG00000037166	chr7	30077635	30077951
ENSMUSG00000001794	chr7	30983787	30984131
ENSMUSG00000036882	chr7	31317895	31318818
ENSMUSG00000074166	chr7	48754974	48755481
ENSMUSG00000014418	chr7	54050693	54051079
ENSMUSG00000052512	chr7	56467998	56468568
ENSMUSG00000065585	chr7	71337594	71338165
ENSMUSG00000033458	chr7	71525282	71526165
ENSMUSG00000030516	chr7	72515785	72516125
ENSMUSG00000078681	chr7	72844400	72845175
ENSMUSG00000042659	chr7	75892500	75892948
ENSMUSG00000062383	chr7	77504714	77505118
ENSMUSG00000078671	chr7	80722591	80723006
ENSMUSG00000025790	chr7	81569455	81569732
ENSMUSG00000030547	chr7	86867178	86867591
ENSMUSG00000039062	chr7	87025719	87026372
ENSMUSG00000030638	chr7	89413176	89413585
ENSMUSG00000070469	chr7	89570924	89571386
ENSMUSG00000070469	chr7	89683611	89684028
ENSMUSG00000001741	chr7	90847964	90848549
ENSMUSG00000052353	chr7	91166657	91167162
ENSMUSG00000052353	chr7	91176875	91177238
ENSMUSG00000039405	chr7	96665805	96666279
ENSMUSG00000039391	chr7	97037660	97038000
ENSMUSG00000062797	chr7	97089597	97090014
ENSMUSG00000030643	chr7	99925617	99925990
ENSMUSG00000030760	chr7	105474927	105475618
ENSMUSG00000070436	chr7	106500431	106501104
ENSMUSG00000070436	chr7	106501446	106502364
ENSMUSG00000070436	chr7	106508768	106509032
ENSMUSG00000047767	chr7	108449646	108451045
ENSMUSG00000037049	chr7	112702685	112703272
ENSMUSG00000073867	chr7	117204657	117205337
ENSMUSG00000030788	chr7	117967070	117967954
ENSMUSG00000030759	chr7	120657726	120658166
ENSMUSG00000084492	chr7	123874752	123875342
ENSMUSG00000033917	chr7	125849154	125849748
ENSMUSG00000046096	chr7	127820758	127821085
ENSMUSG00000030750	chr7	132613406	132613886
ENSMUSG00000030722	chr7	133540387	133540898
ENSMUSG00000042502	chr7	134339494	134340023
ENSMUSG00000045639	chr7	134758465	134758889

ENSMUSG00000030811	chr7	134889262	134889883
ENSMUSG00000030802	chr7	135047327	135047796
ENSMUSG00000030780	chr7	135492051	135492569
ENSMUSG00000055319	chr7	135888255	135888690
ENSMUSG00000030849	chr7	137406432	137407137
ENSMUSG00000030852	chr7	137792624	137793194
ENSMUSG00000040268	chr7	137977870	137978283
ENSMUSG00000066979	chr7	138703130	138703921
ENSMUSG00000073814	chr7	139507141	139507728
ENSMUSG00000030986	chr7	141047956	141048302
ENSMUSG00000041836	chr7	142802855	142803356
ENSMUSG00000025477	chr7	146575469	146575911
ENSMUSG00000025492	chr7	148195969	148196498
ENSMUSG00000031098	chr7	149621199	149622155
ENSMUSG00000052281	chr7	150199684	150200202
ENSMUSG00000052281	chr7	150201733	150202112
ENSMUSG00000009545	chr7	150324813	150325178
ENSMUSG00000059119	chr7	150734705	150735180
ENSMUSG00000048965	chr7	150983773	150984109
ENSMUSG00000031073	chr7	152082426	152083049
ENSMUSG00000070348	chr7	152139881	152140238
ENSMUSG00000070348	chr7	152226734	152227502
ENSMUSG00000004567	chr8	3500017	3500579
ENSMUSG00000019470	chr8	3620996	3621631
ENSMUSG00000040396	chr8	9977382	9978188
ENSMUSG00000039057	chr8	10130940	10131464
ENSMUSG00000031502	chr8	11210498	11211047
ENSMUSG00000031502	chr8	11212340	11212875
ENSMUSG00000031502	chr8	11213334	11213875
ENSMUSG00000031502	chr8	11214437	11214817
ENSMUSG00000031502	chr8	11216488	11217010
ENSMUSG00000031502	chr8	11217743	11218189
ENSMUSG00000031502	chr8	11218404	11219398
ENSMUSG00000031502	chr8	11219963	11220422
ENSMUSG00000031502	chr8	11220717	11221588
ENSMUSG00000031502	chr8	11231650	11232196
ENSMUSG00000031502	chr8	11236161	11236785
ENSMUSG00000031502	chr8	11242439	11243142
ENSMUSG00000031502	chr8	11245485	11246068
ENSMUSG00000031505	chr8	11510003	11510451
ENSMUSG00000074473	chr8	12247892	12248188
ENSMUSG00000038416	chr8	13757395	13757833
ENSMUSG00000071176	chr8	14920538	14921219
ENSMUSG00000059078	chr8	19784282	19784932
ENSMUSG00000031482	chr8	23508462	23508910
ENSMUSG00000008892	chr8	23703984	23704461
ENSMUSG00000031557	chr8	26181480	26181852
ENSMUSG00000054823	chr8	26737558	26738067
ENSMUSG00000031483	chr8	28134828	28135323
ENSMUSG00000039720	chr8	28323562	28323984
ENSMUSG00000083588	chr8	32540054	32540414
ENSMUSG00000046794	chr8	36281301	36281808
ENSMUSG00000031596	chr8	41977425	41977900
ENSMUSG00000031592	chr8	42336077	42336577
ENSMUSG00000031562	chr8	49182580	49183292
ENSMUSG00000031604	chr8	67212545	67212877
ENSMUSG00000074292	chr8	69903980	69904643
ENSMUSG00000051147	chr8	70018191	70018761

ENSMUSG00000030465	chr8	70420674	70421137
ENSMUSG00000044006	chr8	72408073	72408544
ENSMUSG00000002342	chr8	72695958	72696588
ENSMUSG00000003573	chr8	72786515	72786889
ENSMUSG00000070002	chr8	73085866	73086226
ENSMUSG00000000792	chr8	73417339	73418487
ENSMUSG00000074252	chr8	73445721	73446329
ENSMUSG00000002396	chr8	73893004	73893377
ENSMUSG00000034880	chr8	73988361	73988856
ENSMUSG00000034829	chr8	74086617	74087016
ENSMUSG00000052794	chr8	74967764	74968135
ENSMUSG00000037148	chr8	80041509	80041884
ENSMUSG00000019464	chr8	86190592	86191050
ENSMUSG00000065483	chr8	86713295	86713848
ENSMUSG00000001911	chr8	87298905	87299290
ENSMUSG00000001911	chr8	87305484	87305910
ENSMUSG00000001911	chr8	87308723	87309219
ENSMUSG00000033751	chr8	87356323	87356661
ENSMUSG00000041203	chr8	87550355	87550855
ENSMUSG00000031700	chr8	88016660	88017196
ENSMUSG00000036879	chr8	88461663	88461977
ENSMUSG00000045333	chr8	90317723	90318225
ENSMUSG00000056608	chr8	93337515	93337941
ENSMUSG00000056608	chr8	93352889	93353353
ENSMUSG00000031667	chr8	93716675	93717219
ENSMUSG00000033282	chr8	93837095	93837904
ENSMUSG00000055932	chr8	93905492	93906053
ENSMUSG00000031738	chr8	95137275	95137747
ENSMUSG00000031751	chr8	96499874	96500237
ENSMUSG00000033009	chr8	96560953	96561444
ENSMUSG00000031673	chr8	105307746	105308180
ENSMUSG00000063696	chr8	105389267	105389723
ENSMUSG00000031871	chr8	106435676	106436277
ENSMUSG00000031871	chr8	106552500	106553094
ENSMUSG00000041308	chr8	109480928	109481332
ENSMUSG00000065439	chr8	110070504	110071108
ENSMUSG00000031955	chr8	114239936	114240434
ENSMUSG00000031758	chr8	119197659	119198076
ENSMUSG00000034390	chr8	119868577	119869001
ENSMUSG00000034330	chr8	119978517	119978953
ENSMUSG00000034330	chr8	120053883	120054313
ENSMUSG00000034112	chr8	122210145	122210614
ENSMUSG00000042812	chr8	123608367	123609091
ENSMUSG00000064642	chr8	124192737	124193557
ENSMUSG00000040010	chr8	124427380	124427764
ENSMUSG00000025316	chr8	124473684	124474277
ENSMUSG00000006585	chr8	125080254	125080726
ENSMUSG00000006585	chr8	125092625	125093805
ENSMUSG00000015016	chr8	125299072	125299626
ENSMUSG00000065915	chr8	125528753	125529088
ENSMUSG00000031967	chr8	126001645	126002160
ENSMUSG00000039509	chr8	126472914	126473429
ENSMUSG00000031977	chr8	126814878	126815340
ENSMUSG00000074026	chr8	129037527	129038194
ENSMUSG00000074026	chr8	129049491	129049994
ENSMUSG00000051495	chr8	129189550	129189915
ENSMUSG00000074025	chr8	129306177	129306705
ENSMUSG00000072481	chr8	129510283	129510864

ENSMUSG00000025812	chr8	129587766	129588283
ENSMUSG00000084523	chr8	130743410	130743817
ENSMUSG00000041124	chr9	4375891	4376343
ENSMUSG00000047619	chr9	6270041	6270421
ENSMUSG00000050912	chr9	7764469	7764941
ENSMUSG00000032009	chr9	14045592	14045990
ENSMUSG00000032172	chr9	20555909	20556391
ENSMUSG00000004098	chr9	20589542	20590200
ENSMUSG00000004098	chr9	20591184	20591770
ENSMUSG00000004098	chr9	20592886	20593203
ENSMUSG00000004098	chr9	20597788	20598233
ENSMUSG00000032177	chr9	20986946	20987204
ENSMUSG00000032194	chr9	21591370	21591715
ENSMUSG00000031963	chr9	22914397	22914622
ENSMUSG00000008429	chr9	24956166	24956714
ENSMUSG00000032035	chr9	32522150	32522516
ENSMUSG00000032036	chr9	34205504	34205927
ENSMUSG00000032040	chr9	34970418	34971152
ENSMUSG00000038119	chr9	35167249	35167781
ENSMUSG00000032122	chr9	37062762	37063101
ENSMUSG00000001948	chr9	37418294	37418643
ENSMUSG00000040111	chr9	40345318	40345760
ENSMUSG00000032024	chr9	40494472	40495076
ENSMUSG00000032024	chr9	40551429	40551666
ENSMUSG00000032020	chr9	40996274	40996630
ENSMUSG00000032112	chr9	44214684	44215184
ENSMUSG00000063382	chr9	44301815	44302345
ENSMUSG00000063382	chr9	44315949	44316375
ENSMUSG00000048537	chr9	44529146	44529474
ENSMUSG00000039438	chr9	44610756	44611133
ENSMUSG00000002028	chr9	44689255	44690011
ENSMUSG00000034135	chr9	45821812	45822391
ENSMUSG00000037971	chr9	50576109	50576714
ENSMUSG00000061559	chr9	54590239	54590799
ENSMUSG00000032733	chr9	56774146	56774551
ENSMUSG00000032334	chr9	58146773	58147594
ENSMUSG00000032294	chr9	59507060	59507526
ENSMUSG00000032292	chr9	60055565	60055865
ENSMUSG00000032280	chr9	61221494	61221914
ENSMUSG00000053641	chr9	64659675	64660016
ENSMUSG00000050721	chr9	65426043	65426833
ENSMUSG00000065387	chr9	65674607	65675089
ENSMUSG00000050503	chr9	66359553	66360035
ENSMUSG00000032376	chr9	66500044	66500427
ENSMUSG00000079486	chr9	66965765	66966094
ENSMUSG00000034910	chr9	72773416	72773948
ENSMUSG00000007656	chr9	74885065	74885626
ENSMUSG00000058587	chr9	75375031	75375714
ENSMUSG00000032355	chr9	77087093	77087586
ENSMUSG00000065037	chr9	78023728	78024271
ENSMUSG00000074179	chr9	78143950	78144441
ENSMUSG00000032332	chr9	79449810	79450221
ENSMUSG00000032332	chr9	79451994	79452539
ENSMUSG00000032332	chr9	79455974	79456507
ENSMUSG00000032332	chr9	79568708	79569275
ENSMUSG00000032251	chr9	82723191	82723772
ENSMUSG00000066456	chr9	83039936	83040443
ENSMUSG00000062358	chr9	85698691	85699046

ENSMUSG00000056031	chr9	85841943	85842714
ENSMUSG00000033419	chr9	86775654	86776079
ENSMUSG00000037410	chr9	90150924	90151159
ENSMUSG00000032369	chr9	92120546	92120852
ENSMUSG00000050397	chr9	98855534	98855861
ENSMUSG00000056267	chr9	99142616	99143241
ENSMUSG00000044244	chr9	100371288	100371748
ENSMUSG00000074116	chr9	102631620	102632613
ENSMUSG00000074116	chr9	102637015	102637632
ENSMUSG00000084659	chr9	105624980	105625383
ENSMUSG00000043719	chr9	105660452	105660938
ENSMUSG00000064517	chr9	106898763	106899134
ENSMUSG00000037190	chr9	107443919	107444432
ENSMUSG00000010054	chr9	107465119	107465728
ENSMUSG00000070285	chr9	107504525	107504789
ENSMUSG00000034837	chr9	107582661	107583062
ENSMUSG00000039952	chr9	108142293	108142641
ENSMUSG00000025651	chr9	108838904	108839248
ENSMUSG00000049699	chr9	108879699	108880798
ENSMUSG00000032492	chr9	110632389	110632742
ENSMUSG00000045594	chr9	114337339	114337812
ENSMUSG00000039607	chr9	117160751	117161297
ENSMUSG00000061536	chr9	121614277	121614769
ENSMUSG00000046269	chrX	6953053	6953614
ENSMUSG00000082090	chrX	12806851	12807432
ENSMUSG00000079641	chrX	34625032	34625397
ENSMUSG00000031371	chrX	70706324	70706657
ENSMUSG00000031375	chrX	70729350	70729843
ENSMUSG00000031376	chrX	70741904	70742390
ENSMUSG00000046032	chrX	98417695	98418359
ENSMUSG00000073037	chrX	99232874	99233213
ENSMUSG00000051323	chrX	130269392	130269942
ENSMUSG00000083646	chrX	137328238	137328546
ENSMUSG00000081217	chrX	138034532	138034895
