# Supplementary material for: Prdm5 Regulates Collagen Gene Transcription by Association with RNA Polymerase II in Developing Bone
Source: PLoS Genet. 2012 May 10;8(5):e1002711. doi: 10.1371/journal.pgen.1002711 (PMC3349747; doi:10.1371/journal.pgen.1002711)
Supplement: Table S2 — pQCT parameters measured in wild type and Prdm5LacZ/LacZ animals at 5 weeks. P-value by T-test is indicated and cells color-coded according to: yellow = p<0.05, orange = p<0.01, red = p<0.005. (PDF) [file pgen.1002711.s008.pdf]

Galli_Table S2

Total

Total density (mg/mm^2)		Males (n=10)	Females (n=10)
Average	+/+	**440.08**	**386.49**
Average	LacZ/LacZ	**378.20**	**355.92**
Stdev	+/+	**35.49**	**11.34**
Stdev	LacZ/LacZ	**44.12**	**15.76**
pvalue		0.00467	0.00018

Total content (mg)		Males (n=10)	Females (n=10)
Average	+/+	**1.80**	**1.28**
Average	LacZ/LacZ	**1.32**	**1.10**
Stdev	+/+	**0.24**	**0.09**
Stdev	LacZ/LacZ	**0.24**	**0.09**
pvalue		0.00082	0.00037

Total area (mm^2)		Males (n=10)	Females (n=10)
Average	+/+	**4.07**	**3.30**
Average	LacZ/LacZ	**3.48**	**3.09**
Stdev	+/+	**0.34**	**0.15**
Stdev	LacZ/LacZ	**0.27**	**0.20**
pvalue		0.00129	0.01902

Cortical

Cortical density (mg/mm^2)		Males (n=10)	Females (n=10)
Average	+/+	**643.29**	**656.55**
Average	LacZ/LacZ	**605.20**	**623.02**
Stdev	+/+	**21.97**	**16.16**
Stdev	LacZ/LacZ	**37.82**	**14.46**
pvalue		0.01361	0.00003

Cortical content (mg)		Males (n=10)	Females (n=10)
Average	+/+	**1.22**	**0.93**
Average	LacZ/LacZ	**0.85**	**0.77**
Stdev	+/+	**0.22**	**0.07**
Stdev	LacZ/LacZ	**0.17**	**0.06**
pvalue		0.00140	0.00007

Cortical area (mm^2)		Males (n=10)	Females (n=10)
Average	+/+	**1.89**	**1.42**
Average	LacZ/LacZ	**1.40**	**1.23**
Stdev	+/+	**0.32**	**0.09**
Stdev	LacZ/LacZ	**0.23**	**0.08**
pvalue		0.00208	0.00016

Periosteal circumference (mm)		Males (n=10)	Females (n=10)
Average	+/+	**7.15**	**6.44**
Average	LacZ/LacZ	**6.60**	**6.23**
Stdev	+/+	**0.31**	**0.17**
Stdev	LacZ/LacZ	**0.26**	**0.21**
pvalue		0.00124	0.01962

Trabecular

Trabecular density (mg/mm^2)		Males (n=10)	Females (n=10)
Average	+/+	**266.23**	**182.49**
Average	LacZ/LacZ	**227.03**	**177.80**
Stdev	+/+	**20.03**	**9.63**
Stdev	LacZ/LacZ	**32.58**	**9.26**
pvalue		0.00845	0.30614

Trabecular content (mg)		Males (n=10)	Females (n=10)
Average	+/+	**0.58**	**0.34**
Average	LacZ/LacZ	**0.47**	**0.33**
Stdev	+/+	**0.09**	**0.03**
Stdev	LacZ/LacZ	**0.08**	**0.04**
pvalue		0.01218	0.34852

Trabecular area (mm^2)		Males (n=10)	Females (n=10)
Average	+/+	**2.19**	**1.88**
Average	LacZ/LacZ	**2.08**	**1.86**
Stdev	+/+	**0.26**	**0.09**
Stdev	LacZ/LacZ	**0.13**	**0.15**
pvalue		0.30595	0.63877

Endosteal circumference (mm)		Males (n=10)	Females (n=10)
Average	+/+	**6.58**	**5.92**
Average	LacZ/LacZ	**6.24**	**5.87**
Stdev	+/+	**0.32**	**0.15**
Stdev	LacZ/LacZ	**0.18**	**0.21**
pvalue		0.01944	0.50937
