# Supplementary material for: Prdm5 Regulates Collagen Gene Transcription by Association with RNA Polymerase II in Developing Bone
Source: PLoS Genet. 2012 May 10;8(5):e1002711. doi: 10.1371/journal.pgen.1002711 (PMC3349747; doi:10.1371/journal.pgen.1002711)
Supplement: Table S3 — Primers used for qPCR and ChIP-qPCR throughout the study. (PDF) [file pgen.1002711.s009.pdf]

Galli_Table S3

ChIP-qPCR primers

Gene associated	Approx position (relative to TSS in Kb)	Forward sequence	Reverse sequence
actb	TSS	CCCAACACACCTAGCAAATTAGAACCAC	CCTGGATTGAATGGACAGAGAGTCACT
adamts9	TSS	CCTTTTCTGGATGCCTTAACAC	CCTTCACAGTGTTTCCTCTCCT
bgn	+0.5	CCCAGTTGCTAAGCTCACCTAT	CCATGAAGGGATAGGACTATGG
col10a1	+5.1	AGGGTTACCAGGACAAAAAGGT	AGGCACTCCTTTAGCTCCTACA
col11a1	+3.5	TGCCTCAGAAGTGCTGTTTAGA	AGAGCATGCCTGCTGTTTTAAT
col11a2	+19	CATTACCTCTTCCCTTGTTTCG	GCAGTCTCTAGTGGGGAGAAGA
col12a1	-2.2	TGTTTCTTAACTCCCCTTGGAA	ACAGGAGTAAGACCCACAGGAA
col13a1	+6.2	CATGATCAATAACAAGCCTGGA	CCTCCTCTGCATAACCGTTAAA
col14a1	+2.7	GGTATCCTCTCCTTCTGCTTCA	AAACGACTCCAGGAACACTGAC
col15a1	+79	CCAGGGCTAAAAGGAGAACAG	GGGAATCCTTTAAACGTCAATG
col16a1	+10	CACGGGTAAGTACCCTAACCAA	AAAGACCCAGGTACCCCTAGAA
col17a1	+41	ATGAGAGGTCCTCCACACTGAT	CCCAGGGTCTGACTACAGAAAC
col18a1	+33	TCCATGCTCTAATCCCCTTCTA	CCGGTAAGTACTGGTCTCTCCTT
col19a1	+60	CGTACCTGAATACCTGGCTCTC	GAAATGGTGTGTAGGGGTTTTC
col1a1	TSS	GCTCTCCATCAAGATGGTATAAAAG	GACCCTAGACATGTAGACTCTTTGC
col1a1	+1.5	GGAAGAATACGGTACGGTTCTG	AGTTTGGTGATACTGGGGAGAA
col1a1	+3.2	ACTTTGCTTCCCAGATGTCCTA	GACATCATTGGCCTTTCTCTTC
col1a1	+6.2	AGGTCCCAAGGGTAACAGTGTA	CTCGCAAGTGTATGTTTCTCCA
col1a1	+8.9	ATTAAACGCAAAGGGAGGACTT	GCCAGAGTTATTCCAGGAGTGT
col1a1	+11	ACTTCTGACCACCCAATGTTCT	ATACATGTCTCACCCCATCCTC
col1a1	+14	ACCTGGAAACCTTGATCTCCTT	AGGCAGGAAGCTGAAGTCATAA
col1a1	+15.5	GGTCCTCTTGCTTGAAGACCTA	GACTTAAATTTGGGGAGCAATG
col1a2	+4.6	TGGTTCCACAAGATTGCTCTAA	GGGACCATCAACACCATCTCT
col20a1	+14	GACTTTCAGGCTTGCAGGTAAT	CCCTGTGAGGGAACAGAGATAG
col22a1	+135	CAGTGTCCAGGGTGTGTGTACT	ACCCTCTAACATTGGCTGACAT
col23a1	+278	CAAGGTCAGTGTTGCTGAACTC	AGGATGAAGTTCGGGGTACATA
col24a1	+180	TGTCATCCACAGGGTGATAAAG	CCAGTGAGTCAGTGCTGAAAAG
col25a1	+382	AGCACAGCTTCTTAGCAATGGT	CCTTGAAAATTACCTGTTCTCCA
col27a1	+85	GTCAAGGGAGAAGCATAATGGA	GCCAACAGTCCCTGCTATAATC
col28a1	+105	TCTTACAGACACTGTTCCCTGGT	GGCCTCTGGAAAGAAGGTAATC
col2a1	+6.5	CACCCTTGACAGGAAGAGAAAA	GGTGAGCAGACAGCAACAAATA
col3a1	+31	AAATCACCCTCCAATCTGGTAA	CTTACATCCCTTCCAGGTTCAC
col4a1	+70	GGTGACATCACCATCACCATAA	CCCAGATATGGCACTACCTGTT
col4a2	+113	TATGAAGGAGTTGTCCCCTTGT	TGGTCACATGTGCATGAATCTA
col4a3	+82	TCCAGGATGTCCAGGTAAAGAT	CCATGCTCCTATGCCTGAGTAT
col4a4	+75	CAAACAAGCACAGAGAGGCTTA	CTCAGTGAGTGTGTGCTGTTCA
col4a5	+176	GGTATGAATGGTCCTCCTGGTA	ATGAGCACCCTGTTTCTTCTTC
col4a6	+290	GCATTTGTGAGGTATGGACAAA	AAACACTTGGCATTTCTGGAAG
col5a1	+1.1	GCTCTCCTTAGCTTAGCTGGTG	GCCAGAAAGCAGGAGAAGAATA
col5a2	+81	GCCATGGGCTGACATACTTATT	TATGTTGCTTTCCTTTGCTTCA
col5a3	+27	CCCACGTCACCCTAGAACATA	TGACCAAGGGTCCTTGTCTATC
col6a1	+9	ATGGAGAAGCAAATTGGAGCTA	GAGCACTACGTGGAGTCATTTG
col6a2	-10	CCGTAAGTGGCAGAGTTTCATT	AGAAGCACAAGGTCTTTCCTGA
col6a3	+18	GAACTTACCCTTCGACCTCTGA	TGATGTGTCACTTCTTGGCTCT
col6a6	+29	AAAATGGAAAGGACAGCAGTGT	CAAAGGGCCTAAGGTAAGGACT
col7a1	+24	CTGGACTCAAAGGAGACAAGGT	TCCCTAGAGTTCAACCGGAATA
col8a1	+3.4	GGGACCTACTTCACCAAGAAAA	AGAAGGTGGAGTTGTAGGACCA
col8a2	+24	GAGGAGACCAAGGGCCTAAT	CCCAAGTCACCTTTCTGTCCTA
col9a1	+53	ATCCATCAGCTCATTCAAGGAT	ACCTGTTCACCCTTCCACTAAC
col9a2	+1.5	ATGGCATCGACGTGAGTATCTA	GGGGTTTTCTCTGAACAAACAG
col9a3	+3.5	GAGTCTGTGGCTCATTCTTCCT	TAACCTTGGGACAGCAGCTATT
dcn	-103	TCTTTACCAGGGAGCAGAGAAC	ATCCTAGGAGCTTGTTGAGCAC
dmrt3	+0.4	ACAAGCGCTACTGTCGCTTCAA	GCTCGATGATCAGGATGCACTT
emid2	+123	AAGCAAACTCCTGTCTCCACTC	CTATGGCATCTGAGTCCACAGT
fmod	+4.4	CGACCTTTCAGCCTCTTTCTAA	AGTGGGAGGGAAATTAAATGCT
gpc1	-16.8	TGAGGAGTCTTGTTGTCCTCCT	GCTGAGAGCCTTTAGATGTTCA
hoxa9	TSS	ATGTTGACTGGCGATTTTCC	AGAGAACTGCCAACCCTCAA
hoxc8	+0.9	CTGGGAGATTGAAAGGCTTG	ATCTCCCCAACTTTGGCTTT
meg3	TSS	AAACAACGCTCTCCTTTCCTAAG	AAATAACCCCAACTGGTGATTG
mmp14	+1.7	TCTGCTCTAAGTGGGTCATGGT	CTCTCCAGCTCAACATCAAGTCT
pcolce	+0.16	TGTGGAAGCCATCTGAAAGATA	GCTGCCCTAACCTCCTTTCT
postn	-2.5	CATCCTGCTTATGGTTTGTGTG	CATTACACTGCCACGCTTACTC
sema5b	+132	AAACCTCAAAGCCCAGGACACA	ACTTGGTCCCGAGTTGATGGAA
zic3	-2.9	CGCAAGTCTTTCCCAGAGTC	TTGGTATGGGTGGGAGTTTC

Real-time PCR primers

Gene Name	Forward primer	Reverse primer
ActB	CTCCCCGGGCTGTATTCC	CCTCTCTTGCTCTGGGCCTC
Bglap1 (osteocalcin)	AGGAGGGCAATAAGGTAGTGAAC	ACAAGCAGGGTTAAGCTCACA
Col1a1	CGGCTCCTGCTCCTCTTAG	GACCCTTAGGCCATTGTGTATG
Col1a2	GCAGGTTCACCTACTCTGTCCT	AGGCGAGATGGCTTATTTGTT
Dcn	GCTAGACCTGCAAAACAACAAA	GTTTGAATGCCTCTGGACTGAT
Ibsp	AAATGGAGACGGCGATAGTTC	GTCTTCATTCCCCTCAGAATCTT
Prdm5	TCAGAAAAGCGGCCTTATAACTG	CCACTTGATCGAGCCTCTTGAAG
Rpl0	TTCATTGTGGGAGCAGAC	CAGCAGTTTCTCCAGAGC
Rrn18s	TTGACGGAAGGGCACCACCAG	GCACCACCACCCACGGAATCG
Runx2	CGGGAATGATGAGAACTACTCC	CCACAAATCTCAGATCGTTGAA
Sp7 (Osterix)	CTACCCAGCTCCCTTCTCAAG	GGGTGGGTAGTCATTTGCATAG
Ubc	AGCCCAGTGTTACCACCAAG	ACCCAAGAACAAGCACAAGG
