# Supplementary material for: Prdm5 Regulates Collagen Gene Transcription by Association with RNA Polymerase II in Developing Bone
Source: PLoS Genet. 2012 May 10;8(5):e1002711. doi: 10.1371/journal.pgen.1002711 (PMC3349747; doi:10.1371/journal.pgen.1002711)
Supplement: Text S1 — Extended description of experimental procedures including bioinformatics analyses and supplemental references. (DOCX) [file pgen.1002711.s010.docx]

**Supplementary Information S1**

**Prdm5 regulates collagen gene transcription via association with RNA polymerase II in developing bones**

Giorgio Giacomo Galli, Kristian Honnens de Lichtenberg, Matteo Carrara, Wolfgang Hans, Manuela Wuelling, Bettina Mentz, Hinke Arnolda Multhaupt, Cathrine Kolster Fog, Klaus Thorleif Jensen, Juri Rappsilber, Andrea Vortkamp, Les Coulton, Helmut Fuchs, Valérie Gailus-Durner, Martin Hrabě de Angelis, Raffaele Adolfo Calogero, John Robert Couchman and Anders Henrik Lund

**Extended experimental procedures**

Cell culture, differentiation protocols and transfections

293FT, HEK293 and HEK293 cells containing an integrated GAL4TKLuc reporter (kind gift of Klaus Hansen, BRIC, Denmark) were maintained in DMEM with 10% FBS (Hyclone) and 1% penicillin-streptomycin (Gibco). Mouse embryo fibroblasts were isolated from E13.5 embryos from *Prdm5^+/LacZ^* intercrosses and maintained in DMEM with 10% FBS (Gibco) and 1% penicillin-streptomycin (Gibco). MC3T3 cells (kindly provided by Finn Skou Pedersen, Aarhus University, Denmark) were maintained in Alpha-MEM (Gibco) with 10% FBS (Hyclone) and 1% penicillin-streptomycin (Gibco). ATDC5 cells (kindly provided by Vladimir Zachar, Aalborg University, Denmark) were maintained in DMEM:F12 media (Gibco) supplemented with 5% FBS, 10 μg/ml human transferrin (Roche) and 30 nM sodium selenite (Sigma). Calvarial osteoblasts were derived as previously described [21]. Briefly skulls from new-born mice (P0) were dissected in Alpha-MEM+1%P/S and treated first with 0.05% Trypsin (Gibco) and Dispase I (sigma) by shaking at 37ºC. To release osteoblastic cells, 3 sequential treatments with 2.5mg/mL Collagenase A (Roche) were performed followed by serum inactivation and culturing in Alpha-MEM with 10%FBS (Gibco) and 1%P/S. For osteogenic differentiation, media was supplemented with 50 μg/mL ascorbic acid (Sigma) and 10mM beta-glycerol phosphate (Sigma) and replaced every 48 hours. For chondrogenic differentiation, media was supplemented with 10 μg/mL Insulin (Gibco) and replaced every 48 hours. To evaluate calcium nodule formation cells were fixed with 96% Ethanol, stained with 1% Alizarin red (Sigma) and, after washes, Alizarin red was extracted with 10% cetydilpiridinium chloride (Sigma) and absorbance at 562 nm measured using a GloMax ELISA Reader (Promega). To evaluate glycosaminoglycan nodule formation cells were fixed with ice cold methanol and stained with 1% Alcian Blue (Sigma) and, following washes, the staining was extracted with guadinium-hydrochloride (Sigma) and absorbance at 620 nm measured using a GloMax ELISA Reader (Promega). Lentivirus-mediated knockdowns were performed by packaging pLKO.1 constructs (Sigma negative control SHC001, shPrdm5-a TRCN0000081590 and shPrdm5-b TRCN0000081591) into viral particles by cotransfection with pAX8 and VSV plasmids (Addgene) in 293FT cells. Overexpression was performed by transduction with retroviruses produced by transfection of Phoenix packaging cells with pBabe-GFP or pBabe-FLAG-PRDM5 vectors (kind gift of Myriam Alcalay, IFOM, Italy). Transient Prdm5 knockdown was achieved by transfecting 50nM of siRNAs with DharmaFECT1 reagent (Dharmacon) according to manufacturer’s instructions. siRNA sequences against *Prdm5* were obtained from Dharmacon (siPrdm5-1, M-056266) and from Sigma (siPrdm5-2, SASI_Mm01_00157634). Negative control oligo was purchased from Qiagen (“All-star negative”, 1027280).

Micro Computed tomography (micro-CT) and peripheral quantitative computed tomography (pQCT).

Micro-CT scanning (scanner model 1172, Skyscan, Belgium) was performed at 50 kV, 200 µA and a 0.5 mm aluminium filter. The pixel size was 12 µm. Two images were captured every 0.7° through 360° rotation of the sample, the exposure time per image was 295 ms. The x-ray images were reconstructed using Skyscan NRecon software set with a dynamic range of 0 to 0.025 and analyzed using Skyscan CT analysis software. Bone volume was determined for the entire embryo and normalized to total embryo volume. Littermates’ comparisons were analyzed by unpaired T-test with Welch correction.

pQCT analysis was carried out using Stratec XCT Research SA+ (Stratec Medizintechnik GmbH, Germany). The spatial resolution was set to 70 µm, and the distal femoral metaphysis of the left femur from each mouse was examined to obtain volumetric bone mineral density, content and area of the trabecular, cortical, and total bone. The reference line for the CT scans was set at the most distal point of the femur (knee joint space). At 3.0 mm proximal from the reference line, two slices were taken at 0.20 mm intervals and at 6.0 mm proximal from the reference line one slice was taken to give characteristic cross sections of the femoral metaphysis. The CT slices were analyzed using contour mode 1, cortmode 1, and peelmode 2 to evaluate trabecular and cortical parameters. For detection of the outer contour of bone the threshold was set at 350 mg/cm^3^ and the trabecular bone region was defined by setting an inner threshold of 450 mg/cm^3^. Data were analysed by student´s T-test.

Prdm5 antibodies generation

Prdm5 rabbit polyclonal antibodies were generated by immunizing rabbits with a recombinant fragment encompassing amino acids 1-142 of human PRDM5 fused to GST. The serum was affinity purified using columns with immobilized GST-PRDM5 (1-142), while GST-specific antibodies were removed on columns with immobilized GST. The antibodies were concentrated on Hi-trap Protein-G columns (GE-healthcare, cat. 17-0404-03). Mouse serum anti-PRDM5 was obtained by immunizing BALB/c mice with the same immunogen PRDM5 (1-142).

Immunoblotting

Cells were harvested and lysed in RIPA buffer supplemented with protease inhibitor cocktail (Roche). For Decorin core protein detection, cells were pre-treated with 100 μU/μl Chondroitinase ABC (Sigma, EC 4.2.2.4, see (Couchman and Tapanadechopone 2001)). Total proteoglycans from culture media were purified using DEAE-Sephacel (GE Healthcare Life Sciences) upon addition of Urea. Protein samples were resolved on SDS-page, transferred onto nitrocellulose membranes and probed with antibodies against Prdm5 (this study), Vinculin (Sigma, V-9131), GFP (Santa cruz, sc-8334), HA (Biosite, MMS-101P), Collagen I (Abcam, ab34710), GAL4 (Santa cruz, SC-577), Tubulin (Abcam, ab11304), Decorin (R&D systems AF1060), Fibronectin (Yoneda et al. 2007), RNA PolII (Santa Cruz, sc-899), pS2 RNA PolII (Abcam, ab5095) .

Immunofluorescence microscopy

For immunofluorescence staining, limbs from WT and mutant E16.5 embryo littermates were formalin fixed, paraffin embedded in the same block and sectioned at 2 µm. Sections were deparaffinized with xylene and rehydrated through graded alcohols into distilled water. Heat induced antigen retrieval was performed in 0.01 M citrate buffer pH 6.0. For Decorin detection, instead of antigen retrieval, pre-digestion with 100 μU/μl Chondroitinase ABC in PBS pH 7.4, containing 30 mM sodium acetate for 30 minutes at 37°C was applied. After several washes in PBS, the sections were incubated with 5% (v/v) normal serum (NGS or NMS, normal goat or normal mouse serum; Chemicon) in PBS for 20 minutes, followed by primary antibody incubation (diluted in 2% (v/v) NGS or NMS in PBS) for 2 hours. Antibodies used were Osterix (Abcam ab22552), Mmp13 (Millipore AB8120), Vegfa (Abcam ab46154), Collagen I (Abcam ab34710), Decorin (R&D systems AF1060). Sections were thoroughly washed three times in PBS before incubation with Alexa Fluor 647 conjugated IgG (H+L) of appropriate secondary antibodies (Molecular Probes, Invitrogen, diluted in 2% (v/v) NGS in PBS or NMS in PBS) for 1 hour. After final washes in PBS, sections were mounted with Prolong Gold Anti-Fade containing DAPI (Molecular Probes, Invitrogen). Fluorescent images were captured and analyzed on a Zeiss Axioplan-2 microscope and images were processed using Metamorph and Adobe Photoshop CS4.

DNA pull-down

Nuclei from HEK293 cells transfected with empty or HA-PRDM5 overexpressing vector were isolated using the Nuclei EZ prep isolation kit (Sigma) and lysed in HKMG buffer containing 10mM Hepes pH 7.9, 150mM KCl, 5mM MgCl_2_, 10% glycerol, 0.5% Igepal, 1mM DTT (chemicals from Sigma) and protease inhibitor cocktail (Roche). Lysates were pre-cleared with Streptavidin Sepharose beads (GE healthcare) and subsequently incubated overnight with 10 ug polydIdC (Sigma) and 0.2 ug double stranded biotinylated oligos (wild type sense sequence is 5’-GGGTGATACTGGTGCCCCCGGAGCTCCCGGT-3’ and mutated sequence is 5’-GGGTGATACTATTGCCTTCATAGCTTTCTGT-3’). Nucleoprotein complexes were recovered by incubation with Streptavidin Sepharose beads and, after washes, proteins were resuspended in sample buffer for SDS-PAGE analysis.

Chromatin immunoprecipitation

Cells growing in monolayer were cross-linked in 1% formaldehyde for 10 minutes at room temperature after which the reaction was stopped by addition of 0.125M glycine. Cells were lysed and harvested in ChIP buffer (100 mM Tris at pH 8.6, 0.3% SDS, 1.7% Triton X-100, and 5 mM EDTA) and the chromatin disrupted by sonication using a Diagenode Bioruptor sonicator UCD-300 to obtain fragments of 200-500 bp in size. Suitable amounts of chromatin were incubated with specific antibodies overnight. Antibodies used were IgG (Sigma, I8140), Gal4 (Santa cruz, sc-577), commercial Prdm5 (Abgent AP1205A, Abcam ab47066, Lifespan LS-B1982, Santa cruz SC-48653 and SC-48656), Prdm5-Ab1 and Prdm5-Ab2 (described above), H3K4me3 (Cell signaling, 9751), H3K9me3 (Cell signaling, 9754) and RNA PolII (Santa Cruz, sc-899), H3K4me1 (Abcam, ab8895), H3K27ac (Abcam, ab4729). Immunoprecipitated complexes were recovered on Protein-G agarose beads (Pierce) and, after extensive washes, DNA was recovered by reverse crosslinking and purification using QIAquick PCR purification kit (Qiagen). Primer sequences used for ChIP-qPCR experiments are listed in Table S3.

Bioinformatic analyses

ChIP-seq data were mapped over the mouse reference genome (mm9) using SHRIMP software (Rumble et al. 2009) keeping only the first best alignment. Aligned data were filtered to keep only alignments without sequencing errors, with a single unique mapping position and with no more than one mismatch: 39.4 x 10^6^ reads (97.8%) were mapped for the IgG sample, 33.8 x 10^6^ reads (89.2%) for Prdm5-Ab1 and 40.2 x 10^6^ reads (97.2%) for Prdm5-Ab2. Peak segmentation was done using MACS version 1.3.7.1 (Zhang et al. 2008) using as background the IgG data (parameters: FDR<0.1, bw=250, mfold=1). Peaks detected by Prdm5-Ab1 (n=2606) and Prdm5-Ab2 (n=5528) were mapped with respect to TSS using the Bioconductor package ChIPpeakAnno (Zhu et al. 2010) and only those sharing overlaps between Prdm5-Ab1 and Prdm5-Ab2 peaks were retained (n=1712). Subsequent analyses were performed considering peaks associated to Prdm5-Ab1, as peaks generated with this antibody are sharper than the ones with Prdm5-Ab2 probably due to different affinity of Prdm5-Ab1 to Prdm5 protein (data not shown). Prdm5 target regions were classified into four categories, according to their distance from the transcription start site of the closest gene: intergenic (>-20 kb, 316 peaks), upstream (between -20kb and -2 kb, 233 peaks), promoter (between -2kb and +0.5 kb, 496 peaks) and gene body (from +0.5kb to the transcription termination site, 667 peaks). ChIP-seq tracks were generated using Bowtie software (Langmead 2010).

To determine Prdm5 consensus sequence, “high quality” peaks were determined by manual curation. A manual score ranging from 0 to 2 was constructed on the basis of two parameters: 1) the presence of asymmetry between peaks on the plus and the minus strand; 2) maximum peak coverage of at least 20 tags. Score 0 refers to peaks characterized by lack of asymmetry on plus and minus strand. Score 1 refers to peaks characterized by clear asymmetry between plus and minus strand but the maximum coverage was < 20. Score 2 refers to peaks characterized by clear asymmetry between plus and minus strand and a maximum coverage of at least 20.

The Prdm5 consensus sequence was defined using Weeder tool (Pavesi et al. 2004) using only the subset of peaks with a quality score of 2. The sequences defined by the central peak region, located between the plus and minus strand maximum coverage (defined as “shrunk peaks” in the main text), were applied to Weeder. Motifs detected by Weeder were used to generate a Slogos representation of Prdm5 consensus sequence as in (Schneider et al. 1986), using the seqLogo Bioconductor package. The same Slogos representation was made also for the consensus motif identified from the 41 sequences retrieved by [4]. Prdm5 target regions were annotated to gene ontology via the GREAT software (http://great.stanford.edu/public/html/index.php).

Microarray data analysis. Raw intensity data were obtained from Genome Studio 2011.1 (Illumina). Raw data were loaded on oneChannelGUI (Sanges et al. 2007), Log_2_ transformed and normalized using the Loess method (Schmid et al. 2010). Intensity distributions of Prdm5 target genes and all genes in the Illumina beadchip array were compared to each other using a Q-Q plot which is a graphical method for comparing two probability distributions by plotting their quantiles against each other. If the two distributions being compared are similar, the points in the Q-Q plot will approximately lie on the diagonal (represented by a red line).

**Supplemental references**

Couchman JR, Tapanadechopone P (2001) Detection of proteoglycan core proteins with glycosaminoglycan lyases and antibodies. Methods Mol Biol 171: 329-333.

Langmead B (2010) Aligning short sequencing reads with Bowtie. Curr Protoc Bioinformatics Chapter 11: Unit 11 17.

Pavesi G, Mereghetti P, Mauri G, Pesole G (2004) Weeder Web: discovery of transcription factor binding sites in a set of sequences from co-regulated genes. Nucleic Acids Res 32(Web Server issue): W199-203.

Rumble SM, Lacroute P, Dalca AV, Fiume M, Sidow A et al. (2009) SHRiMP: accurate mapping of short color-space reads. PLoS Comput Biol 5(5): e1000386.

Sanges R, Cordero F, Calogero RA (2007) oneChannelGUI: a graphical interface to Bioconductor tools, designed for life scientists who are not familiar with R language. Bioinformatics 23(24): 3406-3408.

Schmid R, Baum P, Ittrich C, Fundel-Clemens K, Huber W et al. (2010) Comparison of normalization methods for Illumina BeadChip HumanHT-12 v3. BMC Genomics 11: 349.

Schneider TD, Stormo GD, Gold L, Ehrenfeucht A (1986) Information content of binding sites on nucleotide sequences. J Mol Biol 188(3): 415-431.

Yoneda A, Ushakov D, Multhaupt HA, Couchman JR (2007) Fibronectin matrix assembly requires distinct contributions from Rho kinases I and -II. Mol Biol Cell 18(1): 66-75.

Zhang Y, Liu T, Meyer CA, Eeckhoute J, Johnson DS et al. (2008) Model-based analysis of ChIP-Seq (MACS). Genome Biol 9(9): R137.

Zhu LJ, Gazin C, Lawson ND, Pages H, Lin SM et al. (2010) ChIPpeakAnno: a Bioconductor package to annotate ChIP-seq and ChIP-chip data. BMC Bioinformatics 11: 237.
